# Comparative efficacy of glioma treatment strategies: an umbrella review of meta-analyses

**DOI:** 10.1080/07853890.2025.2525394

**Published:** 2025-07-01

**Authors:** Erming Yang, Yao Yang, Yixuan Gao, Xinru Liu, Xuanyu Liu, Xiaowen Zong, Jun Li

**Affiliations:** ^a^ Department of Neurosurgery, First Affiliated Hospital of Dalian Medical University, Dalian Loning, China; ^b^ Clinical Medicine, The Second Clinical Medical College of Lanzhou University, Lanzhou, Gansu, China

**Keywords:** Glioma, targeted therapy, immunotherapy, chemotherapy, chemoradiotherapy

## Abstract

**Objective:**

To assess the quality of evidence, identify potential biases, and verify the validity of existing studies on glioma treatment outcomes.

**Methods:**

We extracted and analyzed data from systematic reviews and meta-analyses assessing therapies in glioma patients. Our search included PubMed, Embase, Web of Science, and the Cochrane Database up to July 2024. Two authors independently evaluated each meta-analysis for methodological quality using the AMSTAR tool, following established evidence classification guidelines.

**Results:**

This study evaluated 68 meta-analyses and 389 outcomes from 4,243 articles, classifying 193 outcomes as ‘very low’ quality, 96 as ‘low’ quality, 28 (7.2%) as ‘high’ quality, and 72 (18.5%) as ‘moderate’ quality, 156 as NS, 231 (59.4%) as Class IV, and 2 (0.005%) as Class III, respectively. High-quality evidence demonstrated that combining Traditional Chinese and Western medicine, adding bevacizumab (BVZ) to chemoradiotherapy (CRT), and other specific treatments improved overall survival (OS). Molecularly targeted drugs combined with temozolomide (TMZ) and radiotherapy (RT), as well as standard therapy augmented with anti-vascular endothelial growth factor or BVZ, prolonged progression-free survival (PFS). Additionally, treatments involving RT with TMZ or TMZ alone increased adverse events, whereas high-quality evidence showed that integrated Traditional Chinese and Western medicine, compared to Western medicine alone, reduced bone marrow suppression and gastrointestinal issues, enhancing treatment efficacy.

**Conclusions:**

High-quality evidence indicates that targeted therapy, immunotherapy, CRT, integrated Chinese and Western medicine, gene therapy, and combinations of targeted therapies with CRT or chemotherapy (CT) can extend OS and PFS in gliomas. However, CT, CRT, and targeted therapies, with or without additional CT or CRT, may increase adverse events. Integrated Chinese and Western medicine alone may reduce adverse effects.

## 1. Introduction

Gliomas constitute the most common type of primary malignant brain tumors, accounting for approximately 27% of all central nervous system (CNS) tumors [1]. Data from the Central Brain Tumor Registry of the United States (CBTRUS) for 2016–2020 indicate an average annual age-adjusted incidence rate of CNS tumors in the U.S. at 24.83 per 100,000 individuals [2]. Females exhibit a higher incidence of CNS tumors than males, with higher rates also observed in non-Hispanic populations compared to Hispanic and Black groups. Glioblastoma (GBM), the predominant malignant CNS tumor, comprises 14.2% of all CNS tumors and 50.9% of all malignant brain tumors, showing a greater incidence in males. The estimated average annual mortality rate for CNS tumors is 4.42 per 100,000, equating to about 17,206 deaths annually in the United States [2].

Gliomas, classified by their histopathological features and specific molecular markers as per the 2021 World Health Organization (WHO) classification of CNS tumors [3], represent a diverse group of tumors. These tumors are typically identified during the evaluation of individuals who previously exhibited no health issues but present with focal neurological symptoms, such as muscle weakness or new-onset seizures. The current standard of care involves a combination of maximal tumor resection, RT, and CT with TMZ, with an emphasis on preserving neurological function [4]. However, due to gliomas’ infiltrative growth and common resistance to CT and RT, complete resection is challenging, often resulting in high recurrence rates, mortality, and poor prognosis [5]. Despite numerous meta-analyses of observational studies and RCTs on various glioma therapies published in recent years, inconsistent findings exist across studies due to varying conclusions regarding the effects of treatments such as BVZ, TMZ, RT and immunotherapy. Diverse populations, outcome definitions, study design flaws, and low evidence quality also contributed to these inconsistencies. Apart from that, limited statistical power in conventional systematic reviews or meta-analyses also contributes to the inconsistencies due to their inability to make crosswise comparisons between different treatment modalities. Given these limitations, we designed this umbrella review to provide an opportunity to bring together findings from existing meta-analyses and systematic reviews, offering a broader perspective on the overall quality and consistency of evidence. This approach allows for a more structured comparison of different treatment modalities and helps clarify which strategies are supported by the most reliable data to enable more informed clinical decision-making and guideline development.

Currently, numerous meta-analyses have been conducted to determine the most effective treatment for gliomas, yet no consensus has been reached. A 2016 meta-analysis by Su et al. [6] which included six RCTs involving 1,558 patients, found that combining BVZ with TMZ and RT significantly improved OS. Conversely, a 2022 meta-analysis by Lan et al. [7,8] which included seven RCTs with 3,521 patients, reported that BVZ combined with CRT, including TMZ, did not significantly enhance OS. In addition, despite increasing enthusiasm for immunotherapeutic approaches in glioblastoma, clinical outcomes remain inconsistent, and the therapeutic efficacy of immune checkpoint inhibitors is far from conclusive. For instance, a 2018 study by Omuro et al. [9] including 40 patients demonstrated that while nivolumab monotherapy showed a relatively favorable safety profile, the combination with ipilimumab led to higher toxicity without clear improvements in efficacy. These findings underscore the growing clinical ambiguity surrounding immunotherapy protocols in glioma and the need for more definitive guidance through systematic evidence synthesis. In addition, issues remain regarding the uniformity of treatment across glioma subtypes. Although the 2016 WHO classification by Wen et al. [1], introduced significant refinements in glioma taxonomy by incorporating molecular features into diagnostic criteria, clinical treatment strategies have not kept pace with this molecular heterogeneity. Therapeutic protocols remain largely standardized across histologically and molecularly distinct glioma subtypes, raising concerns about the uniformity and appropriateness of care. This gap further justifies the need for a comprehensive synthesis of evidence to align classification with individualized treatment. Furthermore, the effectiveness of various surgical approaches continues to be debated. Consequently, systematic reviews of the literature, data extraction, and reanalysis of existing meta-analyses on glioma therapies are essential for assessing the reliability of evidence through umbrella reviews, ultimately supporting improved and comprehensive clinical therapies based on the latest evidence-based medicine.

## 2. Methods

### 2.1. Umbrella review methods

We conducted a comprehensive search, extraction, and analysis of data from published meta-analyses investigating the relationships between treatment outcomes and various glioma therapies. Typically, glioma therapies are combined in meta-analyses; thus, we excluded simple systematic reviews that did not include meta-analyses from our umbrella review. This evidence-based analysis followed the Preferred Reporting Items for Systematic Reviews and Meta-Analyses (PRISMA) 2020 Statement. This review was prospectively registered with PROSPERO (CRD42024599416) at https://www.crd.york.ac.uk/PROSPERO/.

### 2.2. Literature search

We searched PubMed, Embase, Web of Science, and the Cochrane Database from inception to 20 July 2024, for systematic reviews and meta-analyses of RCTs and observational studies, using MeSH terms, keywords, and text variations (see Supplementary Table 1). Authors EMY, YY, XRL and XWZ independently screened titles, abstracts, and full texts, with a fifth author, XYG, resolving any disagreements. Reference lists of included articles were also manually checked for missed studies.

### 2.3. Eligibility criteria

We included meta-analyses of randomized controlled trials, cohort, case-control, and cross-sectional studies that assessed therapies for different types of glioma. Eligible meta-analyses had to compare the effects of at least two treatments on the same outcomes using metrics such as RR, OR, HR, WMD, or SMD. For studies reporting multiple outcomes, data were extracted separately for each outcome. When treatments were studied more than once with gaps exceeding three years, only the most recent study-typically the one with the largest sample size-was selected. For studies conducted within the same three-year span, we prioritized the meta-analysis with the greatest number of prospective cohort studies and RCTs, or, if similar, the one with a higher AMSTAR score.

Exclusion criteria for these umbrella reviews included meta-analyses of single therapy treatments; meta-analyses not focused on glioma patients; meta-analyses evaluating therapies for secondary diseases caused by gliomas; and studies in languages other than English, as well as animal and cell culture studies.

### 2.4. Data extraction

Two reviewers (EMY and YY) independently extracted data from each study, including author, publication year, outcomes (overall survival, progression-free survival, adverse events, remission rates), study design (e.g. cross-sectional, case-control, cohort, RCTs), comparisons (e.g. RT plus TMZ vs. RT alone), and effect estimates (risk ratios, odds ratios, weighted mean differences, and standardized mean differences with 95% CIs). Additionally, we recorded the model of effect (random or fixed), heterogeneity (I^2^ statistic and Cochran’s Q test *P*-value), and publication bias (Egger’s test *P*-value or funnel plot analysis). If dose-response or subgroup analyses were performed, we also documented the results and nonlinearity tests’ *P*-values. For meta-analyses that included both cohort and case-control/cross-sectional studies with stratified results by study design, we prioritized cohort design subanalysis for extraction or reanalysis. During the full extraction process, disagreements between the two reviewers were recorded and categorized by type (e.g. outcome classification, effect size reporting, or study design interpretation). These disagreements were then reviewed and adjudicated by a third reviewer (XYL), who was blinded to the initial judgments and resolved discrepancies based on predefined criteria and consultation of the original full-text sources. In cases where the original reporting was ambiguous, resolution followed a consensus discussion involving all three reviewers. This structured approach was used to minimize subjective interpretation and ensure consistency across the dataset.

### 2.5. Quality assessment of methods and evidence

Three reviewers (EMY, XRL and YY) assessed the methodological quality of included articles using the validated AMSTAR tool for systematic reviews and meta-analyses. Evidence quality for each outcome was rated with the GRADE system as ‘high’, ‘moderate’, ‘low’, or ‘very low’ to support conclusions. Evidence was further categorized into Class I (convincing), Class II (highly suggestive), Class III (suggestive), Class IV (weak), and NS (non-significant), with detailed classification criteria in Table 1.

**Table 1.** Evidence categories criteria.

Evidence class	Description
Class I: convincing evidence	>1000 cases (or >20,000 participants for continuous outcomes), statistical significance at *p* < 10^−6^ (random-effects), no evidence of small-study effects and excess significance bias; 95% prediction interval excluded the null, no large heterogeneity (I^2^ < 50%)
Class II: highly suggestive evidence	>1000 cases (or >20,000 participants for continuous outcomes), statistical significance at *p* < 10^−6^ (random-effects) and largest study with 95% CI excluding the null value
Class III: suggestive evidence	>1000 cases (or >20,000 participants for continuous outcomes) and statistical significance at *p* < 0.001
Class IV: weak evidence	The remaining significant associations with *p* < 0.05
NS: non-significant	*p* > 0.05

### 2.6. Data analysis

We reanalyzed risk ratios, odds ratios, weighted mean differences, and standardized mean differences with 95% confidence intervals, using random or fixed effects models as appropriate. For each outcome, the type of effect model (fixed or random) was retained as reported in the original meta-analyses, when both models were reported, we prioritized the random-effects model in line with its wider applicability in the presence of heterogeneity. Heterogeneity (I^2^, Cochran’s Q-test *P*-values) and small-study effects (Egger’s test *p*-values) were calculated for each meta-analysis reporting relevant data. For Class I or II outcomes, sensitivity analyses were performed when possible to assess the impact of individual studies on reliability. If recent meta-analyses excluded studies from prior ones, we merged these datasets for reanalysis. When pooled data from observational studies and randomized trials were available, each study type was reanalyzed separately. Where reanalysis wasn’t feasible, summary data were extracted to assess heterogeneity and publication bias. Heterogeneity significance was set at *p* < 0.10, and other tests at *p* < 0.05. Evidence synthesis was done with Review Manager v5.4; Egger’s tests and sensitivity analyses used Stata v15, and Cohen’s κ statistics were calculated with IBM SPSS Statistics v25.

## 3. Results

### 3.1. Characteristics of meta-analyses

**Figure 1.**
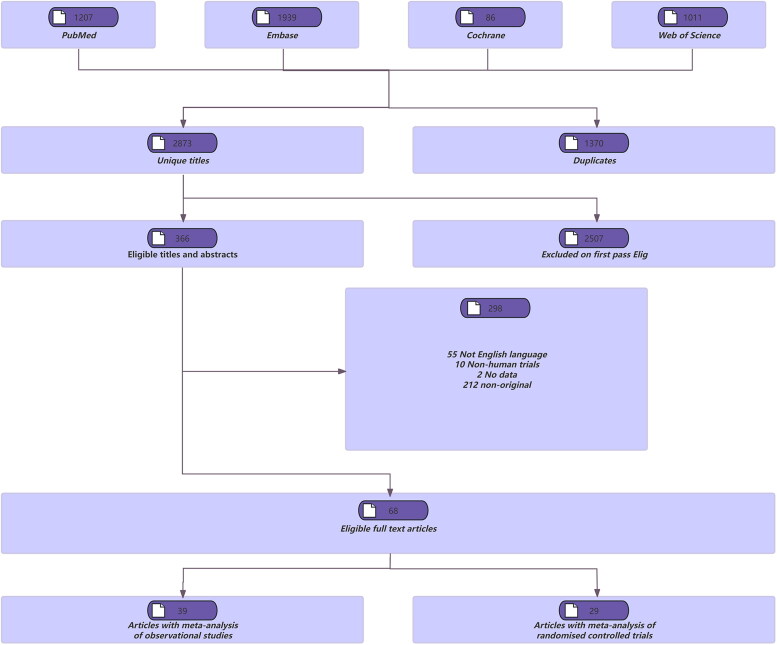
Literature search and screening process.

Figure 1 illustrates the flowchart of our literature search and selection process. We identified 4,243 unique articles through a systematic literature search. From these, applying our inclusion criteria, we derived 68 articles with meta-analyses, comprising 39 from observational studies and 29 from randomized controlled trials (RCTs). We identified 261 unique outcomes from the observational study meta-analyses and 128 from the RCT meta-analyses.

Most included meta-analyses focused on the associations between treatments and various types of gliomas, with 20 targeting GBM, followed by 16 on high-grade glioma, and others on glioma (*n* = 8), recurrent glioma (*n* = 5), and low-grade glioma (*n* = 4). Fewer studies addressed specific subtypes such as malignant glioma (*n* = 3) and IDH-wild-type GBM (*n* = 2). The most common treatment interventions were gross total resection (GTR) (*n* = 57) and awake craniotomy (*n* = 18), with other treatments including sub-total resection (STR) and adjuvant RT (*n* = 14 each). Out of 389 outcomes assessed, 215 showed beneficial associations and 24 harmful associations. The quality of evidence was predominantly rated as low or very low by GRADE criteria, with only 7.2% and 18.5% of outcomes classified as high and moderate quality, respectively. Two outcomes were graded as Class III evidence, with no findings reaching Class I or II evidence levels in this umbrella review.

### 3.2. OS

#### 3.2.1. High quality evidence

##### 3.2.1.1. RT plus TMZ

**Figure 2.**
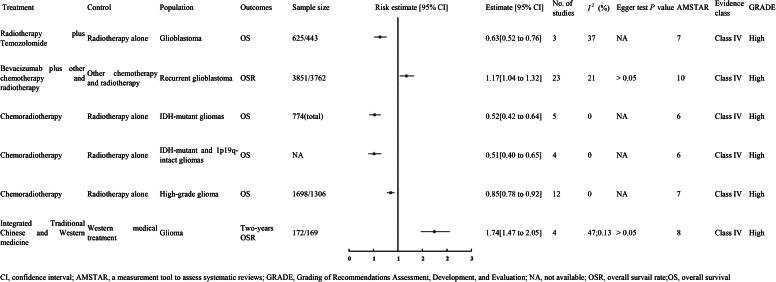
The OS of high quality evidence.

A meta-analysis of three randomized controlled trials with 1,068 patients-625 in the treatment group and 443 in the control group [10], showed that adding TMZ to RT significantly enhanced OS in GBM patients compared to RT alone (HR 0.63, 95% CI 0.52–0.76), with the evidence rated as high quality (Class IV) (Figure 2).

##### 3.2.1.2. BVZ plus other CT and RT

In a meta-analysis of 23 randomized controlled trials involving 7,613 patients with recurrent GBM [11], we found that BVZ combined with CT and RT significantly increased the overall survival rate (OSR) compared to CT and RT alone (OR 1.17, 95% CI 1.04 to 1.32), classified as high-quality evidence (Class IV) (Figure 2).

##### 3.2.1.3. CRT

Analyzing three meta-analyses, we noted that CRT improved OS compared to RT alone. In one meta-analysis of five RCTs with 774 patients with IDH-mutant gliomas, CRT significantly enhanced OS (HR 0.52, 95% CI 0.42 to 0.64, high quality evidence [Class IV]) [10]. Similarly, another meta-analysis of four RCTs focusing on IDH-mutant and 1p19q-intact gliomas showed a favorable outcome (HR 0.51, 95% CI 0.40 to 0.65, high quality evidence [Class IV]) [10]. Similarly, another meta-analysis of four RCTs focusing on IDH-mutant and 1p19q-intact gliomas showed a favorable outcome (HR 0.51, 95% CI 0.40 to 0.65, high quality evidence [Class IV]) (Figure 2) [12].

##### 3.2.1.4. Integrated Traditional Chinese and Western medicine

A meta-analysis of four RCTs with 341 glioma patients (172 experimental, 169 control) showed that combining Traditional Chinese and Western medicine significantly improved the two-year OS rate compared to Western medicine alone (RR 1.74, 95% CI 1.47–2.05; high-quality evidence, Class IV) [13]. Another meta-analysis of three RCTs with 180 patients (90 per group) also demonstrated that this integrated approach significantly boosted the two-year OS rate over Western medicine alone (RR 20.33, 95% CI 4.03–102.59; high-quality evidence, Class IV) (Figure 2) [13].

##### 3.2.1.5. Gene therapy

A meta-analysis of three randomized controlled trials with 508 patients (257 receiving gene therapy and 251 on standard treatment) showed a significant increase in median survival time for high-grade glioma patients treated with gene therapy (MD 0.59, 95% CI 0.41–0.76; high-quality evidence, Class IV) [14].

#### 3.2.2. Moderate quality evidence

##### 3.2.2.1. CRT

A meta-analysis of four randomized controlled trials with 963 anaplastic glioma patients (482 receiving CRT and 481 receiving RT) showed that CRT significantly improved OS (HR 0.84, 95% CI 0.72–0.98; high-quality evidence, Class IV) [15].

**Figure 3.**
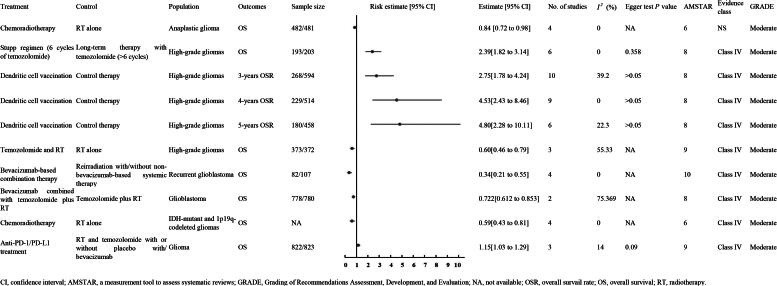
The OS of moderate quality evidence.

Additionally, another meta-analysis of three RCTs with patients having IDH-mutant and 1p19q-codeleted gliomas showed similar improvements in OS with CRT versus RT alone (HR 0.59, 95% CI 0.43 to 0.81, high quality evidence [NS]) (Figure 3) [10].

##### 3.2.2.2. Long-term therapy with TMZ

A meta-analysis of six non-randomized studies involving 396 patients with high-grade gliomas (193 in the Stupp regimen group and 203 in the long-term therapy group) demonstrated that the Stupp regimen (six cycles of TMZ) significantly improved OS compared to long-term TMZ therapy (HR 2.39, 95% CI 1.82 to 3.14, moderate quality evidence [Class IV]) (Figure 3) [16].

##### 3.2.2.3. Dendritic cell vaccination

A series of meta-analyses evaluated the efficacy of dendritic cell vaccination in patients with high-grade gliomas. The first, comprising one RCT and nine non-randomized controlled studies (NCRs) with 862 patients (268 in the experimental group and 594 in the control group), demonstrated a significant increase in three-year survival rates (RR 2.75, 95% CI 1.78 to 4.24, moderate quality evidence [Class IV]) [17]. The second analysis, including one RCT and eight NCRs with 743 patients (229 experimental, 514 control), showed improved four-year survival rates (RR 4.53, 95% CI 2.43 to 8.46, moderate quality evidence [Class IV]) [17]. The third, involving six NCRs with 638 patients (180 experimental, 458 control), indicated enhanced five-year survival rates (RR 4.80, 95% CI 2.28 to 10.11, moderate quality evidence [Class IV]) (Figure 3) [17].

##### 3.2.2.4. TMZ and RT

A meta-analysis of three RCTs with 745 high-grade glioma patients (373 receiving TMZ plus RT and 372 receiving RT alone) showed that TMZ combined with RT significantly improved OS (RR 0.60, 95% CI 0.46–0.79; moderate-quality evidence, Class IV) (Figure 3) [18].

##### 3.2.2.5. BVZ-based combination therapy

A meta-analysis of four cohort studies with 189 recurrent GBM patients (82 receiving BVZ-based therapy and 107 in the control group) showed that BVZ significantly improved OS compared to reirradiation with or without non-BVZ therapy (HR 0.34, 95% CI 0.21–0.55; moderate-quality evidence, Class IV) (Figure 3) [19].

##### 3.2.2.6. BVZ combined with TMZ plus RT

A meta-analysis of two RCTs with 1,558 GBM patients (778 receiving BVZ with TMZ and RT, 780 in the control group) found that adding BVZ significantly improved OS compared to TMZ and RT alone (HR 0.722, 95% CI 0.612–0.853; moderate-quality evidence, Class IV) (Figure 3) [6].

##### 3.2.2.7. Anti-PD-1/PD-L1 treatment

A meta-analysis of three randomized controlled trials with 1,645 glioma patients (822 in the anti-PD-1/PD-L1 group and 823 in the control group) indicated that anti-PD-1/PD-L1 treatment was linked to a reduced OS compared to RT and TMZ, with or without placebo or BVZ (HR 1.15, 95% CI 1.03–1.29; moderate-quality evidence, Class IV) (Figure 3) [20].

###### 3.2.3. Low quality evidence

Resection, compared to biopsy, significantly reduced mortality in elderly patients (≥60 years) with high-grade gliomas [21]. Additionally, GTR versus STR was linked to enhanced 5-year survival [22], and reduced mortality at 2 and 5 years in patients with low-grade gliomas [23], GTR also lowered 1-year and 2-year mortality rates in children with infratentorial high-grade gliomas [24], and markedly increased the 3-year, 10-year, and 15-year OS in glioma patients [25]. Moreover, GTR demonstrated improved OS in patients with isocitrate dehydrogenase-wild-type GBMs compared to biopsy or STR [26].

Dendritic cell vaccination, compared to standard therapy [27], carmustine wafers versus the Stupp regimen [28], and viral therapy with optimized injection methods alongside standard of care, versus standard care alone [29], were associated with improved OS in patients with high-grade gliomas. Additionally, combination therapy, compared to reirradiation [19], improved OS in patients with recurrent GBM. In patients with GBM, both carmustine implantation versus no treatment [30] and CRT versus RT alone [31] significantly improved OS. Meta-analysis also showed that carmustine implantation enhanced OS across various pathological grades of glioma [30]. Furthermore, cytoreductive resection versus biopsy extended OS in elderly patients (≥65 years) with supratentorial high-grade glioma [32]. In recurrent malignant glioma, hypofractionated stereotactic RT plus CT, compared to RT alone, improved OS [33]. Lastly, awake craniotomy versus asleep craniotomy [34] and adjuvant CRT versus RT alone [35] were each linked to better OS outcomes in patients with eloquent glioma and oligodendroglioma, respectively.

An improvement in the 2-year OS rate was associated with dendritic cell vaccination compared to control therapy in patients with high-grade glioma [17] and with the use of vaccines as opposed to conventional treatments in patients with malignant glioma [36].

Furthermore, tumor treating fields therapy combined with the standard of care, especially when device usage exceeded a 75% threshold, was linked to prolonged OS in patients newly diagnosed with GBM [37].

#### 3.2.4. Very low quality evidence

In our study, resection compared to biopsy was significantly associated with increased OS in patients with butterfly GBM [38] and in elderly patients (≥60 years) with high-grade gliomas [21], as well as reduced 1-year mortality in GBM patients [39]. Furthermore, 5-aminolevulinic acid-guided surgical resection, as opposed to conventional microsurgical resection, prolonged the mean difference in OS in patients with high-grade gliomas [40]. In elderly patients (≥60 years) with high-grade gliomas, both STR and GTR, compared to biopsy, significantly prolonged OS [21]. GTR also prolonged OS in patients with low-grade gliomas [22], all gliomas [25], high-grade gliomas [25], and isocitrate dehydrogenase-wild-type GBMs [26]. Additionally, GTR was linked to reduced 10-year mortality in low-grade glioma patients [39], increased 1-year, 2-year, and 5-year OS in glioma patients [25], and decreased 1-year and 2-year mortality in GBM patients [39].

STR compared to biopsy was associated with significantly reduced 2-year mortality in patients with low-grade glioma [23] and infratentorial pediatric high-grade gliomas [24], 1-year mortality in GBM [39], and improved OS in patients with isocitrate dehydrogenase-wild-type GBM [26] and low-grade glioma [22]. Resection, compared to biopsy, in low-grade glioma was linked to increased OS [22] and reduced mortality at 2, 5, and 10 years [23]. Additionally, supramaximal resection, as opposed to GTR, enhanced OS in GBM patients [41]. Similarly, supratotal resection versus GTR was linked to improved OS in GBM patients [42]. Moreover, GTR compared to biopsy significantly improved OS in patients with isocitrate dehydrogenase-wild-type GBM [26]. Cytoreductive resection compared to biopsy was associated with prolonged OS in patients with supratentorial high-grade glioma, across a wide age range, including those with Grade IV/4 and Grade III or IV gliomas [32].

Additionally, we observed that combination therapy, as opposed to systemic therapy or reirradiation, prolonged OS in patients with recurrent high-grade gliomas [19]. Furthermore, in the same study, BVZ-based combination therapy, compared to reirradiation with or without non-BVZ-based systemic therapy or systemic therapy alone, was associated with extended OS in these patients. Reirradiation also prolonged OS compared to systemic therapy alone.

Furthermore, our findings indicate that immunotherapy, compared to standard care treatments including surgical resection, RT, or CT, was associated with increased 1-year, 2-year, and 3-year OS in glioma patients [43]. Additionally, our analysis revealed that vaccines, as opposed to conventional treatments, were associated with improved OS in patients with malignant glioma, including primary malignant and high-grade gliomas [36].

We observed a significant association between adjuvant RT, compared to either salvage RT or no RT, and improved OS in patients with oligodendroglioma, including those defined by IDH mutation with 1p/19q co-deletion and Grade 3 oligodendroglioma. Additionally, adjuvant RT was associated with enhanced OS in patients with oligodendroglioma compared to those receiving salvage RT [35].

Additionally, we observed that viral therapy, compared to standard therapy, prolonged OS in patients with high-grade gliomas [27]. Similarly, dendritic cell vaccination was associated with prolonged 1-year OS compared to control therapy in these patients [17]. Meta-analyses by Guo et al. [29] revealed that both immunotherapy combinations with standard of care, and dendritic cell therapy combinations, improved OS in high-grade gliomas. Carmustine implantation, versus TMZ CT or the combination of TMZ and carmustine, was linked to extended OS in GBM patients [30]. Furthermore, significant associations were found between OS improvement in patients with glioma or GBM and the combined use of BVZ and TMZ [44], as well as between OS improvement and levetiracetam plus standard of care, compared to standard care alone in GBM [45]. Additionally, hypofractionated stereotactic RT combined with CT, compared to hypofractionated stereotactic RT alone, enhanced OS in patients with recurrent malignant glioma [33]. In contrast, TMZ combined with hypofractionated radiation therapy, versus standard radiation therapy, was associated with decreased OS in elderly GBM patients [46].

### 3.3. PFS

#### 3.3.1. High quality evidence

##### 3.3.1.1. Targeted therapy plus Lomustine

**Figure 4.**
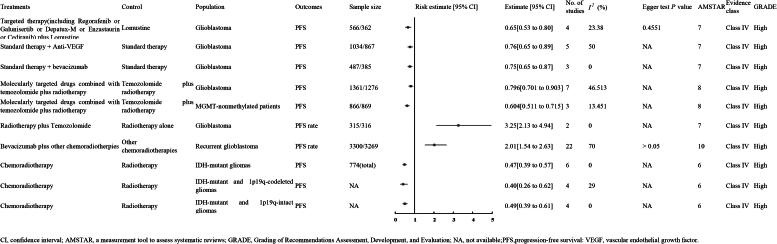
The PFS of high quality evidence.

A meta-analysis of four RCTs involving 928 GBM patients-566 in the experimental group and 362 in the control group-demonstrated that targeted therapy (including Regorafenib, Galunisertib, Depatux-M, Enzastaurin, or Cediranib) combined with Lomustine significantly improved outcomes compared to Lomustine alone (RR 0.65, 95% CI 0.53 to 0.80) (evidence level: high; Class IV) (Figure 4) [47].

##### 3.3.1.2. Standard therapy plus anti-vascular endothelial growth factor

An analysis of a meta-analysis encompassing five RCTs with 1,091 GBM patients-1,034 in the experimental group and 867 in the control group-found that adding anti-vascular endothelial growth factor to standard therapy significantly improved PFS compared to standard therapy alone (OR 0.76, 95% CI 0.65–0.89; high-quality evidence, Class IV) (Figure 4) [48].

##### 3.3.1.3. Standard therapy plus BVZ

An analysis pooling data from three randomized controlled trials, which included 872 patients with GBM-487 assigned to the experimental group and 385 to the control group-revealed that incorporating BVZ into the conventional treatment markedly enhances PFS compared to the conventional treatment on its own (Odds Ratio 0.75, 95% Confidence Interval 0.65 to 0.87; high evidence level; Class IV) (Figure 4) [48].

##### 3.3.1.4. Molecularly targeted drugs combined with TMZ plus RT

Su et al. [6] conducted two meta-analyses demonstrating that molecularly targeted drugs combined with TMZ and RT significantly improved PFS compared to TMZ and RT alone. The first meta-analysis, which included seven RCTs with a total of 2,637 GBM patients (1,361 in the experimental group and 1,276 in the control group), showed a hazard ratio (HR) of 0.796 (95% CI 0.701 to 0.903; evidence level: high; Class IV). The second analysis, encompassing three RCTs with 1,735 patients with MGMT-nonmethylated GBM (866 in the experimental group and 869 in the control group), reported an HR of 0.604 (95% CI 0.511 to 0.715; evidence level: high; Class IV) (Figure 4).

##### 3.3.1.5. RT plus TMZ

A meta-analysis of two RCTs with 631 GBM patients (315 treated and 316 controls) showed that adding TMZ to RT significantly improved PFS over RT alone (HR 0.604, 95% CI 0.511–0.715; high-quality evidence, Class IV) (Figure 4) [49].

##### 3.3.1.6. BVZ plus other CT and RT

A meta-analysis of 22 RCTs with 6,569 recurrent GBM patients-3,300 in the experimental group and 3,269 in the control group-showed that adding BVZ to CT and RT significantly increased PFS compared to CT and RT alone (HR 0.604, 95% CI 0.511–0.715; high-quality evidence, Class IV) (Figure 4) [11].

##### 3.3.1.7. CRT

Kinslow et al. [10] conducted three meta-analyses demonstrating that CRT significantly improved PFS compared to RT alone. The analyses included six RCTs with 774 patients with IDH-mutant gliomas (HR 0.47, 95% CI 0.39 to 0.57; evidence level: high; Class IV), four RCTs with patients with IDH-mutant and 1p19q-codeleted gliomas (HR 0.40, 95% CI 0.26 to 0.62; evidence level: high; Class IV), and four RCTs with IDH-mutant and 1p19q-intact gliomas (HR 0.49, 95% CI 0.39 to 0.61; evidence level: high; Class IV) (Figure 4).

###### 3.3.1.8. BVZ plus some specific cytotoxic treatments.

A meta-analysis of four RCTs with 465 recurrent GBM patients (248 on BVZ plus cytotoxic therapy, 217 controls) showed that adding BVZ significantly extended median PFS compared to cytotoxic therapy alone (MD 0.71, 95% CI 0.28–1.15; moderate-quality evidence, Class IV) [50].

#### 3.3.2. Moderate quality evidence

##### 3.3.2.1. CRT

**Figure 5.**
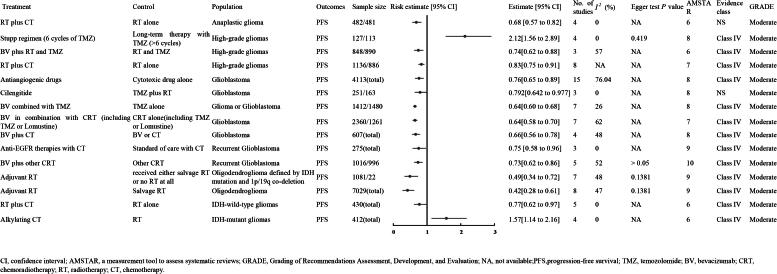
The PFS of moderate quality evidence.

Three meta-analyses showed that adding CT to RT significantly improved PFS over RT alone: one with four RCTs and 963 anaplastic glioma patients (HR 0.68, 95% CI 0.57–0.82; moderate-quality evidence, NS) [15], another with four RCTs and 2,022 high-grade glioma patients (HR 0.83, 95% CI 0.75–0.91; moderate-quality evidence, Class IV [12], and a third with five RCTs and 430 IDH-wild-type glioma patients (HR 0.77, 95% CI 0.62–0.97; moderate-quality evidence, Class IV) (Figure 5) [10].

##### 3.3.2.2. Long-term therapy with TMZ

A meta-analysis of four nonrandomized studies with 240 high-grade glioma patients-127 receiving long-term TMZ and 113 following the standard regimen-showed that extended TMZ therapy significantly increased PFS compared to the six-cycle Stupp regimen (HR 2.12, 95% CI 1.56–2.89; moderate-quality evidence, Class IV) (Figure 5) [16].

##### 3.3.2.3. *BVZ plus RT and TM*Z

A meta-analysis of three RCTs with 1,738 high-grade glioma patients (848 on BVZ plus RT and TMZ, 890 controls) found that adding BVZ significantly improved PFS compared to RT alone (HR 0.74, 95% CI 0.62–0.88; moderate-quality evidence, Class IV) (Figure 5) [51].

##### 3.3.2.4. Dendritic cell vaccination

A meta-analysis of one randomized controlled trial and two NCRs involving 196 patients with high-grade gliomas-48 in the experimental group and 148 in the control group-demonstrated that dendritic cell vaccination significantly increased the 2-year PFS rate compared to control therapy (HR 8.59, 95% CI 2.94 to 25.08; evidence level: moderate; Class IV) (Figure 5) [17].

##### 3.3.2.5. *Antiangiogenic drug*s

A meta-analysis of 15 RCTs with 4,113 GBM patients showed that antiangiogenic drugs significantly improved PFS over cytotoxic drugs alone (HR 8.59, 95% CI 2.94–25.08; moderate-quality evidence, Class IV) (Figure 5) [52].

##### 3.3.2.6. *Cilengitid*e

A meta-analysis of three RCTs involving 414 GBM patients-251 in the experimental group and 163 in the control group-demonstrated that cilengitide improved PFS compared to TMZ plus RT (HR 0.792, 95% CI 0.642 to 0.977; evidence level: moderate; NS) (Figure 5) [6].

##### 3.3.2.7. *BVZ combined with TM*Z

A meta-analysis of seven RCTs with 2,892 GBM patients (1,412 on BVZ plus TMZ, 1,480 controls) showed that adding BVZ significantly improved PFS over TMZ alone (HR 0.64, 95% CI 0.60–0.68; moderate-quality evidence, Class IV) (Figure 5) [44].

##### 3.3.2.8. *BVZ in combination with CR*T

A meta-analysis of seven RCTs involving 3,621 GBM patients-2,360 in the experimental group and 1,261 in the control group-demonstrated that BVZ combined with CRT (including TMZ or lomustine) significantly prolonged PFS compared to CRT alone (HR 0.64, 95% CI 0.58 to 0.70; evidence level: moderate; Class IV) (Figure 5).

Additionally, another meta-analysis of five RCTs including 2,012 patients with recurrent GBM-1,016 in the experimental group and 996 in the control group-showed that BVZ with CRT significantly improved PFS compared to CT and RT alone (HR 0.73, 95% CI 0.62 to 0.86; evidence level: moderate; Class IV) (Figure 5) [11].

##### 3.3.2.9. BVZ plus CT

A meta-analysis of four RCTs involving 607 GBM patients demonstrated that BVZ combined with CT significantly prolonged PFS compared to BVZ or CT alone (HR 0.66, 95% CI 0.56 to 0.78; evidence level: moderate; Class IV) [8] (Figure 5).

##### 3.3.2.10. Anti-EGFR therapies with CT

A meta-analysis of three RCTs with 275 patients found that adding anti-EGFR therapy to CT significantly improved PFS in recurrent GBM compared to CT alone (HR 0.75, 95% CI 0.58–0.96; moderate-quality evidence, Class IV) (Figure 5) [53].

##### 3.3.2.11. Adjuvant RT

Two meta-analyses [35] demonstrated that adjuvant RT significantly prolonged PFS in patients with oligodendroglioma. The first meta-analysis of seven studies showed improved PFS compared to either salvage RT or no RT in patients with oligodendroglioma defined by IDH mutation and 1p/19q co-deletion (HR 0.49, 95% CI 0.34 to 0.72; evidence level: moderate; Class IV). The second meta-analysis of eight studies found adjuvant RT improved PFS compared to salvage RT alone in patients with oligodendroglioma (HR 0.42, 95% CI 0.28 to 0.61; evidence level: moderate; Class IV) (Figure 5).

##### 3.3.2.12. Alkylating CT

A meta-analysis of four RCTs with 412 IDH-mutant glioma patients showed that alkylating CT reduced PFS compared to RT (HR 1.57, 95% CI 1.14–2.16; moderate-quality evidence, Class IV) (Figure 5) [10].

#### 3.3.3. Lower quality evidence

Resection, compared to biopsy, was associated with prolonged PFS in elderly patients (≥60 years) with high-grade gliomas [21]. Dendritic cell vaccination, compared to control therapy, improved 3-year and 4-year PFS in patients with high-grade gliomas [17]. TMZ combined with RT, compared to RT alone, was associated with improved PFS in high-grade glioma patients [18]. Additionally, patients with recurrent high-grade glioma and recurrent GBM showed significantly better PFS when treated with combination therapy compared to reirradiation [19]. Improved PFS was also observed in patients with recurrent GBM receiving BVZ-based combination therapy versus reirradiation, with or without non-BVZ-based systemic therapy [19]. GTR, compared to STR, was associated with a decreased 1-year progression risk in patients with infratentorial pediatric high-grade gliomas [24]. In GBM patients, targeted therapy compared to TMZ [47] and molecularly targeted drugs compared to TMZ plus RT [54] were linked to significantly prolonged PFS. GTR, compared to STR or biopsy, was also significantly associated with improved PFS in patients with isocitrate dehydrogenase–wild-type GBM [26]. Additionally, a significant 2-year PFS rate was observed in malignant glioma patients treated with vaccines compared to conventional treatments [36]. Meta-analyses by Ng et al. [35] showed that adjuvant RT, compared to either salvage RT or no RT, improved PFS in patients with oligodendroglioma or Grade 2 oligodendroglioma, and adjuvant CRT, compared to CT alone, was associated with better PFS in oligodendroglioma patients. However, anti-PD-1/PD-L1 treatment was not associated with improved PFS in glioma patients [20].

#### 3.3.4. Very low quality evidence

GTR, compared to STR, was significantly associated with prolonged PFS in patients with low-grade glioma [22], and isocitrate dehydrogenase-wild-type GBM [26]; reduced progression at 2, 5, and 10 years in low-grade glioma patients [23]; decreased 6-month progression in infratentorial pediatric high-grade gliomas [24]; reduced 1-year progression in GBM patients [39]; and improved 1-, 3-, and 5-year PFS in glioma patients [25]. Supratotal resection, compared to GTR or STR, was associated with improved PFS in GBM patients [42]. Additionally, supramaximal resection, compared to GTR, was linked to prolonged PFS in patients with GBM and IDH wild-type GBM [41]. Resection, compared to biopsy, was associated with decreased 1-year progression in GBM patients [39]. GTR, compared to biopsy, showed a significant association with prolonged PFS in patients with isocitrate dehydrogenase-wild-type GBM [26].

Early radiation, compared to late or no radiation, was associated with decreased progression at 2, 5, and 10 years [23].

BVZ-based combination therapy, compared to reirradiation with or without non-BVZ-based systemic therapy and systemic therapy alone, was associated with improved PFS in patients with recurrent high-grade glioma [19].

Additionally, CT, compared to RT, showed a significant association with reduced progression at 5 and 10 years in patients with low-grade glioma [23].

5-Aminolevulinic acid–guided surgical resection, compared to conventional microsurgical resection [40], dendritic cell vaccination compared to standard therapy [27], and combined immunotherapy with standard care compared to standard care alone [29] were all associated with prolonged PFS in patients with high-grade gliomas. In recurrent GBM patients, reirradiation compared to systemic therapy and combination therapy compared to systemic therapy were associated with increased PFS [19].

Immunotherapy compared to standard care (including surgical resection, RT, or CT) in glioma patients [43], carmustine implantation compared to non-carmustine treatment in GBM patients [30], awake versus asleep craniotomy in patients with eloquent glioma [34], adjuvant CRT compared to adjuvant RT in oligodendroglioma patients [35], and adjuvant RT compared to salvage or no RT in Grade 3 oligodendroglioma patients [35] were all associated with improved PFS.

### 3.4. Adverse events

#### 3.4.1. High quality evidence

##### 3.4.1.1. TMZ

**Figure 6.**
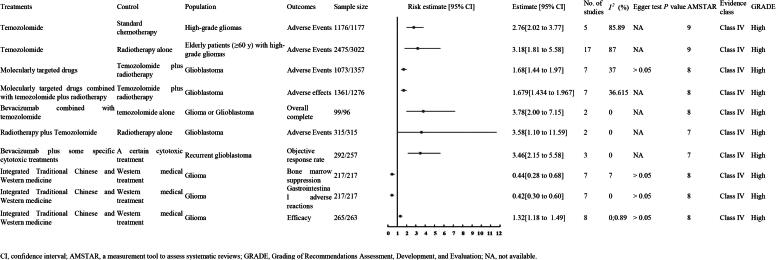
Other outcomes of high quality evidence.

A meta-analysis of five RCTs involving 2,353 patients with high-grade gliomas (1,176 in the experimental group and 1,177 in the control group) found that TMZ, compared to standard CT, was associated with a higher incidence of adverse events (OR 2.76, 95% CI 2.02 to 3.77; evidence level: high; Class IV) (Figure 6) [18].

Additionally, a meta-analysis of 17 RCTs involving 5,497 elderly patients (≥60 years) with high-grade gliomas (2,475 in the experimental group and 3,022 in the control group) found that TMZ, compared to RT alone, was linked to an increased risk of adverse events (OR 3.18, 95% CI 1.81 to 5.58; evidence level: high; Class IV) (Figure 6) [18].

##### 3.4.1.2. Molecularly targeted drugs

A meta-analysis of seven RCTs with 2,430 GBM patients (1,073 experimental, 1,357 control) showed that molecularly targeted drugs were significantly linked to more adverse events than TMZ plus RT (OR 1.68, 95% CI 1.44–1.97; high-quality evidence, Class IV) (Figure 6) [54].

##### 3.4.1.3. RT plus TMZ

A meta-analysis of two RCTs involving 630 patients (315 in the experimental group and 315 in the control group) found that RT plus TMZ was associated with increased adverse events compared to RT alone (RR 3.58, 95% CI 1.10 to 11.59; evidence level: high; Class IV) (Figure 6) [49].

##### 3.4.1.4. Integrated Traditional Chinese and Western medicine

A meta-analysis of seven RCTs involving 434 glioma patients (217 in the experimental group and 217 in the control group) demonstrated that integrated Traditional Chinese and Western medicine was associated with reduced bone marrow suppression (RR 0.44, 95% CI 0.28 to 0.68; evidence level: high; Class IV), decreased gastrointestinal adverse reactions (RR 0.42, 95% CI 0.30 to 0.60; evidence level: high; Class IV), and increased treatment efficiency (RR 1.32, 95% CI 1.18 to 1.49; evidence level: high; Class IV) compared to Western medical treatment alone, based on three meta-analyses of 7, 7, and 8 RCTs respectively (Figure 6) [13].

##### 3.4.1.5. Molecularly targeted drugs combined with TMZ plus RT

A meta-analysis of seven RCTs involving 2,637 GBM patients (1,361 in the experimental group and 1,276 in the control group) found that molecularly targeted drugs combined with TMZ and RT were associated with increased adverse effects compared to TMZ and RT alone (OR 1.679, 95% CI 1.434 to 1.967; evidence level: high; Class IV) [6].

#### 3.4.2. Moderate quality evidence

##### 3.4.2.1. Dose-dense TMZ

**Figure 7.**
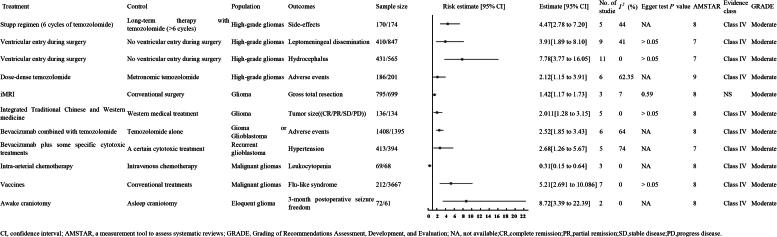
Other outcomes of moderate quality evidence.

A meta-analysis of six RCTs with 287 high-grade glioma patients (186 experimental, 101 control) found that dose-dense TMZ was significantly linked to more adverse events than metronomic TMZ (OR 2.12, 95% CI 1.15–3.91; moderate-quality evidence, Class IV) (Figure 7) [18].

##### 3.4.2.2. BVZ combined with TMZ

A meta-analysis of six RCTs involving 2,803 patients with glioma or GBM (1,408 in the experimental group and 1,395 in the control group) found that BVZ combined with TMZ was significantly associated with increased adverse events compared to TMZ alone (OR 2.52, 95% CI 1.85 to 3.43; evidence level: moderate; Class IV) (Figure 7) [44].

#### 3.4.3. Low quality evidence

Combination therapy with BVZ and CT, compared to BVZ or CT alone, was associated with a significant increase in adverse events in GBM patients [8]. Conversely, adjuvant RT, compared to adjuvant CT alone, was linked to a significant reduction in adverse events in patients with Grade 3 to 5 oligodendroglioma [35].

#### 3.4.4. Very low quality evidence

Combination therapies involving immunotherapy, viral therapy, or immunopotentiators with standard care, compared to standard care alone, were associated with a significant increase in adverse events in patients with high-grade gliomas [29]. Additionally, adjuvant CRT, compared to adjuvant RT, was significantly associated with increased adverse events in patients with Grade 3 to 5 oligodendroglioma [35].

### 3.5. Other outcomes

#### 3.5.1. High quality evidence

##### 3.5.1.1. BVZ combined with TMZ

A meta-analysis of two RCTs involving 195 patients with glioma or GBM (99 in the experimental group and 96 in the control group) indicated that BVZ combined with TMZ significantly improved the overall complete remission rate compared to TMZ alone (OR 3.78, 95% CI 2.00 to 7.15; evidence level: high; Class IV) [44].

##### 3.5.1.2. BVZ plus some specific cytotoxic treatments

A meta-analysis of three RCTs involving 549 patients with recurrent GBM (292 in the experimental group and 257 in the control group) found that BVZ combined with specific cytotoxic treatments was significantly associated with an increased objective response rate compared to a single cytotoxic treatment (RR 3.46, 95% CI 2.15 to 5.58; evidence level: high; Class IV) [50].

#### 3.5.2. Moderate quality evidence

##### 3.5.2.1. Long-term therapy with TMZ

A meta-analysis of five nonrandomized studies involving 244 patients with high-grade gliomas (170 in the experimental group and 174 in the control group) found that long-term TMZ therapy (>6 cycles) was associated with significantly fewer side effects compared to the Stupp regimen (6 cycles of TMZ) (RR 4.47, 95% CI 2.78 to 7.20; evidence level: moderate; Class IV) [16].

##### 3.5.2.2. Ventricular entry during surgery

Two meta-analyses [55] indicated that ventricular entry during surgery was associated with increased risks of leptomeningeal dissemination and hydrocephalus compared to no ventricular entry in patients with high-grade gliomas. One meta-analysis of nine studies involving 1,257 patients (410 in the experimental group and 847 in the control group) reported an odds ratio (OR) of 3.91 (95% CI 1.89 to 8.10; evidence level: moderate; Class IV), while the other meta-analysis of 11 studies with 996 patients (431 in the experimental group and 565 in the control group) reported an OR of 7.78 (95% CI 3.77 to 16.05; evidence level: moderate; Class IV).

##### 3.5.2.3. iMRI

A meta-analysis of three RCTs involving 1,494 glioma patients (795 in the experimental group and 699 in the control group) found that intraoperative MRI (iMRI) was associated with increased rates of gross total resection compared to conventional surgery (RR 1.42, 95% CI 1.17 to 1.73; evidence level: moderate; NS) [56].

##### 3.5.2.4. Integrated Traditional Chinese and Western medicine

A meta-analysis of five RCTs with 270 glioma patients (136 experimental, 134 control) showed that combining traditional Chinese and Western medicine significantly improved tumor control rates compared to Western treatment alone (RR 0.42, 95% CI 0.30–0.60; moderate-quality evidence, Class IV) [13].

##### 3.5.2.5. BVZ plus some specific cytotoxic treatments

A meta-analysis of five RCTs involving 807 patients with recurrent GBM (413 in the experimental group and 394 in the control group) found that BVZ combined with specific cytotoxic treatments was associated with increased hypertension compared to a single cytotoxic treatment (RR 0.42, 95% CI 0.30 to 0.60; evidence level: moderate; Class IV) [50].

##### 3.5.2.6. Intra-arterial CT

A meta-analysis of three RCTs involving 137 patients with malignant gliomas (69 in the experimental group and 68 in the control group) found that intra-arterial CT was associated with reduced leukocytopenia compared to intravenous CT (RR 0.31, 95% CI 0.15 to 0.64; evidence level: moderate; Class IV) [57].

##### 3.5.2.7. Vaccines

A meta-analysis of seven studies involving 3,879 patients with malignant gliomas (212 in the experimental group and 3,667 in the control group) indicated that vaccine therapy was associated with an increased incidence of flu-like syndrome compared to conventional treatments (RR 5.21, 95% CI 2.691 to 10.086; evidence level: moderate; Class IV) [36].

##### 3.5.2.8. Awake craniotomy

A meta-analysis of two studies involving 133 patients with eloquent glioma (72 in the experimental group and 61 in the control group) showed that awake craniotomy was associated with increased 3-month postoperative seizure freedom compared to asleep craniotomy (OR 8.72, 95% CI 3.39 to 22.39; evidence level: moderate; Class IV) [34].

#### 3.5.3. Low quality evidence

GTR, compared to STR, was associated with significantly increased tumor recurrence in adult pilocytic astrocytoma [58], higher seizure control and lower malignant transformation in low-grade glioma [22], and improved 5-year local control in glioma patients [25].

CT combined with RT, compared to BVZ or CT alone, was significantly associated with an increased objective response rate in GBM patients [8].

Additionally, vaccine therapy, compared to conventional treatments, was significantly linked to increased skin reactions in patients with malignant glioma [36].

Awake craniotomy, compared to asleep craniotomy, was associated with reduced 3-month postoperative neurological deficits and improved 3-month postoperative Karnofsky performance scores in patients with eloquent glioma [34].

Fluorescein-guided surgery, compared to standard resection without fluorescein, was associated with higher rates of gross total resection in patients with high-grade gliomas [59].

#### 3.5.4. Very low quality evidence

Resection, compared to biopsy, was associated with significantly improved postoperative Karnofsky performance status in elderly patients (≥60 years) with high-grade gliomas. Additionally, GTR, compared to STR, was linked to decreased recurrence and improved seizure control in glioma patients [25].

Furthermore, iMRI, compared to conventional surgery, was associated with increased gross total resection and mean extent of resection [56].

Awake craniotomy, compared to asleep craniotomy, was associated with an increased extent of resection in patients with eloquent glioma and GBM, as well as a reduced hospital stay for those with eloquent glioma [34]. Additionally, awake craniotomy, compared to general anesthetic resection, was linked to fewer late neurological deficits in glioma patients [60].

A significant association was also found between TMZ and unmethylated or methylated tumors in elderly GBM patients, compared to RT [61].

Furthermore, lower cumulative risk was observed in glioma patients treated with hormone replacement therapy or awake craniotomy compared to RT and TMZ [62]. Lastly, adjuvant CRT, compared to adjuvant RT, was associated with more adverse events in patients with Grade 3 to 5 oligodendroglioma [35].

### 3.6. Heterogeneity

In our study, heterogeneity was present in 70% of all outcomes. Reanalysis indicated significant heterogeneity (I^2^ > 50%) in approximately 40% of health outcomes. Potential factors explain the heterogeneity in most of the following outcomes. Firstly, the heterogeneity may mainly be ascribed to differences in demographic and research contexts, including setting, region, ethnicity, sex, age, genetics, diet and medical condition, which significantly impact treatment response and prognosis to some degree. In addition, various study designs may also contribute to inconsistent results. We analyzed RCTs, prospective or retrospective cohort studies, case-control studies and various other studies, which inherently featured different levels of evidence and risks of bias. In addition, differences in sample size, follow-up time, and adjustment for confounding factors further contributed to the inconsistency of the results. What’s more, heterogeneity may also originate from the implementation of treatment protocols. Differences existed in the protocol, dose, cycle or combination strategy of the same treatment across different studies. Surgical methods may be another source of heterogeneity,as differences in the specific scope of STR may influence patient outcomes. Additionally, the differences in biological characteristics of tumors are also a source of heterogeneity that cannot be ignored. Tumor subtypes covered in different studies inherently differ in treatment response, especially in studies with ambiguous tumor classification. Apart from that, the GRADE classification showed that most of the evidence was of low or very low quality, which may further contribute to the heterogeneity.

### 3.7. Assessment of risk of bias

Our Egger test revealed publication bias in three outcomes: targeted therapy versus lomustine in GBM (*p* = 0.0297), GTR versus STR in elderly patients (≥60 years) with high-grade gliomas (*p* = 0.02), and GTR versus STR in glioma (*p* = 0.044). Publication bias tests were performed for 79.5% of the outcomes, including 40.5% with Egger’s test and 38.7% with funnel plots without Egger’s test, and 38.7% of the outcomes undergo funnel plots without Egger test (24.2% showed publication bias, and 75.8% showed no publication bias).

### 3.8. AMSTAR, GRADE, and evidence classification

The median AMSTAR score for all health outcomes was 8 (range 4–10; IQR 7–9) (Supplementary Table 2), with individual scores in Supplementary Table 3. Evidence from cohort, case-control, and cross-sectional meta-analyses was generally rated low to moderate due to observational design and quality downgrades (e.g. bias, inconsistency, imprecision). Of 31 RCT meta-analyses, 12 were high quality, and 21 were downgraded to moderate due to inconsistency, imprecision, or publication bias. Remaining studies were rated low or very low quality due to similar issues (Supplementary Table 2).

Supplementary Table 4 presents the detailed GRADE classification for each outcome. Regarding evidence classification, the association between GTR and STR with 1-year and 2-year mortality in GBM patients was graded as class III. Of the remaining 387 outcomes, 231 (59.2%) were graded as Class IV, while 156 (40%) were identified as non-significant (Supplementary Table 4).

## 4. Discussion

### 4.1. Principal findings

Glioma treatment outcomes vary across approaches such as GTR, awake craniotomy, STR, and adjuvant RT. From 4,243 articles, we identified 68 meta-analyses with 261 outcomes, including 261 unique outcomes from observational studies and 128 from RCTs.

### 4.2. The importance of targeted therapy for the treatment of gliomas

Various targeted therapies have been associated with improved outcomes in glioma treatment, including combinations of targeted agents (Regorafenib, Galunisertib, Depatux-M, Enzastaurin, or Cediranib) with lomustine, standard therapies with anti-vascular endothelial growth factor inhibitors, molecular therapies with TMZ and RT, BVZ in conjunction with other chemotherapies and RT, molecular therapies, BVZ with TMZ, BVZ with specific cytotoxic agents, combination therapies based on BVZ, BVZ with TMZ and RT, antiangiogenic agents, cilengitide, BVZ with CRT including TMZ or lomustine, BVZ and CT, and CT with anti-EGFR therapies.

#### 4.2.1. BVZ

Most therapies under discussion predominantly utilize BVZ. Analysis revealed a significant association of BVZ with enhanced PFS, OS, objective response rates, and complete remission rates. However, the analysis also indicated a significant correlation between BVZ and increased adverse events, including hypertension. BVZ, a humanized monoclonal antibody targeting VEGF-165-a major isoform of VEGF-A-became the first angiogenesis inhibitor approved by the FDA in 2004, marking the initial commercial success of an antiangiogenic agent [63]. BVZ downregulates angiogenesis by binding to circulating vascular endothelial growth factor A, altering its interaction with endothelial receptors, thus reducing tumor blood supply [64]. This mechanism suppresses tumor growth, supporting the use of BVZ as a promising treatment option for gliomas. Research also suggests BVZ enhances tumor oxygenation and radiosensitivity, potentially improving RT outcomes [65]. Concurrently, BVZ reduces symptomatic radiation necrosis by decreasing vascular permeability. Additionally, BVZ may bolster immunotherapy’s efficacy by inhibiting dendritic cell maturation and promoting regulatory T cell proliferation [66].

Future research should focus on combining BVZ’s role in vasculature normalization with enhanced delivery of drugs and tumor-killing immune cells to mitigate tumor cell invasion, destroy tumor cells, and lessen toxicity from other treatments [67]. Additionally, future efforts will also emphasize preventing treatment resistance and recurrence to enhance the cost-effectiveness of BVZ and overall treatment strategies, as the economic aspects of antiangiogenic regimens in gliomas are of greater concern to patients than to clinicians [67]. Furthermore, due to pseudoprogression and pseudoresponse induced by BVZ, more appropriate response criteria should be applied in future studies [65]. Moreover, to maximize the benefits of BVZ, research should explore increasing synergistic antitumor effects through its combination with other treatment modalities, like various chemotherapeutic agents or surgical interventions. Exploring new administration routes or drug carriers for BVZ also represents a promising avenue; recent studies suggest that intranasal administration of BVZ may facilitate its transport to the central nervous system [68]. Intranasally administered BEV-loaded poly (D,L-lactic-co-glycolic) acid nanoparticles have demonstrated significant antiangiogenic effects [69]. Moreover, although BVZ has been associated with various benefits, more reliable biomarker-enrichment strategies are needed to determine the continuation of therapies.

#### 4.2.2. Cilengitide

Meta-analyses suggest cilengitide improves PFS without increasing toxicity or adverse events. Cilengitide is a cyclized pentapeptide that inhibits integrin-mediated adhesion and migration [70]. It potently and selectively blocks αvβ3 and αvβ5 integrins, disrupting cell attachment, migration, and differentiation in response to growth factors *in vitro* [71–73] and inhibiting angiogenesis *in vivo*, thus suppressing tumor growth without affecting existing blood vessels [74–76]. Cilengitide induces apoptosis in U87 glioma cells by inhibiting adherence to vitronectin and tenascin, proteins that mediate brain tumor invasion and growth [77]. Additionally, cilengitide synergizes with radio-immunotherapy in treating GBMs [78].

Future research will investigate additional combinatorial regimens and more reliable biomarkers to guide treatment, aiming to achieve personalized dosing for patients with gliomas. Furthermore, the mechanisms driving improved outcomes in patients with MGMT-methylated tumors warrant further investigation.

#### 4.2.3. Cediranib

Cediranib, an indole-ether quinazoline with a molecular weight of 450.51, potently inhibits VEGF signaling ATP-competitively by binding to the intracellular domains of three VEGF receptor tyrosine kinases, primarily through inhibition of VEGFR-2/Flk-1/KDR tyrosine kinase activity [79]. We observed an association with improved PFS in gliomas. Consequently, cediranib may inhibit not only VEGFR-2-mediated angiogenesis but also VEGFR-3-mediated lymphangiogenesis, thereby suppressing tumor growth [80].

However, cediranib also induces side effects, including hypertension-due to its impact on VEGF’s role in regulating vasomotor tone and blood pressure-and a marginally increased risk of bleeding and wound dehiscence [81]. Common systemic side effects also include fatigue, nausea, weight loss, and diarrhea.

Moreover, cediranib can alleviate tumor-associated vasogenic cerebral edema-a significant cause of morbidity and mortality in gliomas-through vascular normalization [82–84]. This leads to improved PFS and OS through edema control alone, even without significant tumor growth inhibition [85]. Indeed, cediranib also exhibits a steroid-sparing effect, reducing both short-term and long-term complications, thereby enhancing patient quality of life and neurological function. Additionally, cediranib significantly inhibits the tyrosine kinase activity of c-Kit and platelet-derived growth factor receptors alpha and beta (PDGFR-α, PDGFR-β).

Future research will incorporate advanced imaging modalities and biomarkers to more accurately assess anti-tumor responses, treatment effects, and toxicities, given that current MRI contrast enhancement parameters may be misleading. Furthermore, future studies will investigate combining cediranib with other therapies, such as CT or RT, to reduce side effects and improve the management of tumor cell infiltration and host vasculature co-option previously identified [86].

#### 4.2.4. Dasatinib

Contrary to the results above, we observed that combining dasatinib with BVZ was associated with decreased OS and PFS in patients with GBM. Dasatinib, a potent SRC and SFK tyrosine kinase inhibitor, is approved for treating certain leukemia types, primarily due to its effectiveness against BCR-ABL fusion proteins that drive these malignancies [87]. It suppresses GBM cell growth and prevents tumor invasion and metastasis by inhibiting SRC and SFK, key regulators in GBM [88]. Additionally, dasatinib may enhance the anti-tumor effects of NK cells in targeting glioma cells [89]. However, dasatinib might also impair the overall anti-tumor immune response by inhibiting regulatory T cells (Tregs) conversion and proliferation, potentially leading to decreased OS and PFS [89]. Furthermore, a study indicated that dasatinib is associated with cardiovascular adverse reactions, which may result in harmful outcomes [90]. Moreover, tumor cell resistance following long-term dasatinib treatment also contributes to the decline in OS and PFS [89].

In the future, researchers plan to combine dasatinib with other therapies, particularly immunotherapies like vaccines, to mitigate its side effects and enhance its anti-tumor efficacy. Additionally, further optimization of dasatinib’s dosage and administration regimens, tailored to the physical conditions of patients-particularly those with cardiovascular disease-is required. Moreover, future research will focus on overcoming resistance to dasatinib, including the identification of novel biomarkers, to facilitate personalized therapy.

Notably, the potential advantages of targeted therapies vary depending on molecular profiles and tumor subtypes. For instance, BVZ appears particularly beneficial in patients with recurrent GBM, offering symptomatic relief through edema control and prolonging PFS. Cediranib demonstrates steroid-sparing effects that may improve quality of life in selected patients. However, the benefits are often not uniform across populations, and agents like dasatinib have shown paradoxical outcomes, possibly due to immune modulation or resistance mechanisms. Therefore, while targeted agents provide new therapeutic avenues, their advantages are most pronounced in molecularly defined subsets rather than across unselected patient populations.

#### 4.2.5. Anti-EGFR

We observed that anti-EGFR therapies, including tyrosine kinase inhibitors, monoclonal antibodies, and targeted vaccines, were associated with improved PFS in patients with recurrent GBM. Recently, anti-EGFR therapies have been employed in the treatment of both GBM and low-grade gliomas [91]. EGFR, one of the most prevalent oncogenic mutation sites in cancer cells [92], is associated with tumor cell proliferation, migration, and evasion of apoptosis [93]. Anti-EGFR therapies can suppress tumor growth by selectively binding to the inactive configuration of EGFR’s extracellular domain, blocking the ligand-binding region and competing for receptor binding [94].

Future research will further explore novel methods to overcome blood-brain barrier (BBB) blockage. Moreover, more reliable therapeutic strategies are required to overcome resistance to EGFR inhibitors. Additionally, future studies will need to identify more effective and personalized sequences and combinations of EGFR inhibitors, integrating them with other therapies such as CT and RT, to enhance cytotoxicity potentiation.

### 4.3. The importance of immunotherapy for the treatment of gliomas

In our meta-analysis, dendritic cell vaccination was associated with improved OS, however, PD-1/PD-L1 inhibitors did not prolong OS, and vaccinations were more likely to cause flu-like symptoms.

#### 4.3.1. Vaccination

Vaccination has demonstrated significant potential for both preventing and treating gliomas [95]. Cancer vaccines target tumor-associated antigens to induce an immune response that destroys glioma cells. Peptide vaccines, dendritic cell vaccinations (DCVs), and autologous formalin-fixed tumor vaccines (AFTVs) are principal therapeutic strategies for gliomas [96]. Additionally, personalized vaccines represent a promising strategy.

Peptide vaccines, consisting of 8–25 amino acids, feature epitopes that serve as antigenic targets. Additionally, they are typically combined with carrier proteins to enhance immunogenicity [97,98]. Recent studies have focused on targeting EGFRvIII with peptide vaccines. A recent double-blind, randomized, phase II trial (NCT01498328) demonstrated that rindopepimut combined with standard BVZ resulted in better outcomes compared to BVZ alone in patients with recurrent EGFRvIII-positive GBM [99]. In summary, recent trials have shown promising developments in peptide vaccines; future studies will focus on combining these vaccines with angiogenesis inhibitors.

Dendritic cell vaccines consist of effective antigen-presenting cells (APCs) that provoke immune responses. DCs are generated *in vitro* by culturing CD14 monocytes with granulocyte-macrophage colony-stimulating factor and IL-4 to differentiate into immature DCs. Subsequently, these DCs, loaded with tumor antigens, are reinjected into the patient [100]. DCs are believed to initiate both intrinsic and adaptive immune responses, transforming immunologically cold gliomas into hot ones [101,102]. In 2011, a phase II clinical trial of high-grade gliomas using a DCV activated by irradiated tumor cells indicated a median survival time of 520 days, with 18.8% of patients surviving beyond five years [103]. For GBM, DCV treatment prolonged PFS in newly diagnosed patients [104]. Conversely, a phase II clinical study using tumor lysate DCV in patients with recurrent GBM indicated no significant survival increase [105]. Furthermore, a phase I clinical study demonstrated promising efficacy for a DCV targeting the epidermal growth factor receptor variant III (EGFRvIII) [106]. However, the efficacy and safety of DCVs targeting tumor-associated antigens remain controversial. A study that primed DCVs with antigens such as WT-1, HER2, MAGE-A3, MAGE-A1, and gp100 reported poor prognoses [107]. Conversely, a phase I and a randomized phase II trial found promising efficacy for DCVs using antigens such as HER2, TRP-2, gp100, MAGE-1, IL-13Rα2, and AIM-2 [107,108].

AFTV is a vaccine that uses formalin-fixed paraffin-embedded tumor sections to deliver tumor antigens, stimulating the expansion and long-term activation of cytotoxic T lymphocytes (CTLs) to target and kill tumor cells [109]. Its effectiveness has been demonstrated across various cancers [36], with previous studies showing promising results, particularly for metastatic cancers, leading to improved OS and PFS rates. AFTV also outperforms CD-based vaccines, B cell hydroma, tumor-initiating cell vaccines, and other vaccine types in terms of survival duration [36].

Advancements in next-generation sequencing and bioinformatics tools have increased the potential to identify tumor neoantigens, which arise from somatic mutations and are tumor-specific [110,111]. Personalized vaccines can be developed by effectively triggering de novo T cell responses against tumor neoantigens, which are highly specific to individual patients. Initial studies on personalized vaccines have shown strong tumor-specific immunogenicity and preliminary evidence of anti-tumor activity in patients with high-risk melanoma and other cancers [111].

Future studies will focus on enhancing vaccines to modulate the tumor microenvironment, suppressing glioma evasion and growth, while simultaneously improving local immunosuppression. Researchers will also explore more precise tumor antigens with high immunogenicity, even in highly heterogeneous gliomas, to increase vaccine efficacy and shorten treatment regimens, thereby reducing chronic immune toxicity and long-term implications. Given the various targets and synergistic mechanisms of vaccines and other therapies, such as CRT or targeted therapy, combined treatments may significantly improve therapeutic outcomes for glioma and GBM patients. However, extensive research is needed to identify novel biomarkers, establish scientific patient selection criteria, and optimize treatment regimens to minimize potential side effects. Additionally, more research is required to investigate population differences.

#### 4.3.2. Anti-PD-1/PD-L1 treatment

PD-L1 is an immune checkpoint molecule involved in programmed cell death. Its activation inhibits T-lymphocyte activity and promotes immune escape by cancer cells [112]. PD-L1 is expressed in human glioma tissue and correlates with glioma grade [113]. Previous studies have shown that therapeutic inhibition of PD-L1 significantly reduces tumor-infiltrating Treg cell numbers and prolongs OS [114]. Another study found that blocking PD-1 increases the cytotoxicity of NK cells against glioma stem cell-like cells. These findings suggest that PD-L1 may serve as a target for cancer treatment [115]. Nivolumab, a human IgG4 anti-PD-1 monoclonal antibody, has been shown to be better tolerated and achieves a better median OS compared to combined regimens [9]. Pembrolizumab, a humanized monoclonal antibody against PD-1, has demonstrated robust clinical activity and acceptable safety, and was also analyzed in our meta-analysis. However, our study shows contrasting results, which may be due to the high tumor heterogeneity and complex tumor microenvironment in glioma patients, as well as the low PD-L1 expression levels. Additionally, infiltrating T cells in glioma tissue may be in a state of exhaustion, similar to chronic viral infections, leading to significant T cell dysfunction. This could be a key factor contributing to our findings. Furthermore, the blood-brain barrier (BBB) may limit the effectiveness of anti-PD-1/PD-L1 therapies.

Combining anti-PD-1/PD-L1 therapy with other treatments shows promising potential. One study demonstrated that combining anti-PD-1 immunotherapy with stereotactic radiosurgery (SRS) prolonged OS [116]. Additionally, combining anti-PD-1 immunotherapy, an anti-TIM-1 antibody, and SRS significantly improved OS in a GBM mouse model compared to other treatments [117]. Combination therapy with PD-1 inhibitors and VEGF inhibitors has shown good tolerability and promise in both animal models and clinical trials [118,119]. Furthermore, triple combination therapy, consisting of an anti-PD-1 monoclonal antibody, GVAX, and an anti-OX40 monoclonal antibody, was highly effective against murine intracranial gliomas [120]. Given these studies, future research will focus on exploring effective combinations of anti-PD-1/PD-L1 therapy with other treatments, such as targeted therapy or CRT, which may more effectively address evasion mechanisms in the tumor microenvironment. Additionally, new administration methods, such as intracranial catheters for bypassing the blood-brain barrier (BBB), are being explored to improve drug delivery [121]. Moreover, investigating immune escape mechanisms in gliomas and identifying potential biomarkers for PD-1/PD-L1 inhibitor efficacy is crucial. Future studies will also focus on enhancing T-cell infiltration into tumors to improve the efficacy of anti-PD-1/PD-L1 therapies.

### 4.4. The importance of combination or using alone of CT and RT

#### 4.4.1. CT

We found that TMZ was associated with more adverse events than standard CT or RT in glioma patients. However, dose-dense TMZ was associated with fewer adverse events than metronomic TMZ in patients with high-grade gliomas. Intra-arterial CT was linked to increased leukocytopenia compared to intravenous CT. Long-term TMZ therapy (>6 cycles) improved OS and PFS with fewer side effects compared to the Stupp regimen (6 cycles of TMZ) in high-grade glioma patients. Conversely, alkylating CT was associated with decreased PFS in IDH-mutant glioma patients compared to RT.

TMZ, an alkylating agent of the triazene compound class, methylates DNA to damage tumor cells, ultimately inducing apoptosis [122]. It is currently the standard of care for gliomas and can be administered orally in an outpatient setting. By crossing the blood-brain barrier, it maximizes effectiveness while minimizing systemic toxicity. However, resistance due to the methylguanine methyltransferase (MGMT) DNA repair system remains a major cause of treatment failure. Although MGMT effectively repairs TMZ-induced DNA damage, the enzyme is depleted in the process [123]. This mechanism has prompted the exploration of dose-dense and long-term TMZ therapy, which may reduce resistance by depleting MGMT, thereby improving PFS and OS.

TMZ also exhibits immunomodulatory properties. When combined with vaccine immunotherapy, it increases serum IL-2 levels, promoting the maturation of naïve T cells into effector/memory T cells and expanding antigen-specific CD8 T cells in murine models, thereby improving survival [124]. Additionally, TMZ enhances responses to tumor antigens by erasing memory T cells, which boosts the efficacy of immunotherapy [125]. Furthermore, TMZ increases the proportion of Tregs and may enhance dendritic cell function, thereby altering the tumor microenvironment [122].

Future studies will investigate the impact of dosing and timing of TMZ combined with immune checkpoint inhibitors, and explore new evaluation criteria and biomarkers to guide glioma treatment and enable more personalized therapy regimens. Future trials will also examine the effects of TMZ on patients of different ages, determining the optimal regimen for each age group to achieve the most effective outcomes with minimal adverse effects. Additionally, the effects of combining TMZ with other therapies, such as RT or immunotherapy, should be explored to enhance treatment efficacy through synergies and reduce resistance and toxicity.

Procarbazine, another alkylating CT agent, generates nucleophilic diazonium ions that methylate RNA and DNA at nucleophilic sites, damaging tumor cells and inducing apoptosis. It is metabolically activated in the liver by cytochrome P450 (CYP3A4) [126]. Procarbazine also produces O6-alkylating agents [127], which cause DNA double-strand breaks, apoptosis, and cellular senescence in MGMT-proficient cells [126]. These mechanisms support the use of procarbazine in glioma patients. Increasing evidence suggests that senescence is not a completely dormant state; senescent cells can be reactivated to proliferate, leading to tumor recurrence. Therefore, administering senotherapeutics during and after procarbazine therapy is essential. As MGMT repairs critical toxic and senescence-inducing lesions caused by TMZ and procarbazine [126], future trials will focus on combining alkylating CT with MGMT inhibitors or other agents that block repair proteins. Additionally, future investigations will explore more accurate modes of procarbazine administration to directly target tumors and minimize side effects. Given that procarbazine is activated by cytochrome P450, future studies should combine it with other drugs or therapies to enhance its efficacy.

Intra-arterial CT has been used for primary brain tumors since the 1950s and 1960s [128,129]. It enhances drug concentration in tumors through regional delivery, particularly for tumors dependent on arterial blood supply, such as gliomas. However, the blood-brain barrier (BBB) and the short dwell time of rapidly cleared drugs limit its effectiveness. Another challenge is the uneven distribution of the drug within tumors or the brain due to poor mixing or streaming of the drug solution in the artery. Future trials will focus on strategies to enable intra-arterial CT to permeate or bypass the BBB while controlling the drug delivery rate, especially within tumors, to extend dwell time and improve tumor cell kill [57]. By controlling dwell time, uniform drug distribution within tumors may be achievable, potentially enhancing the effectiveness of intra-arterial CT over intravenous administration. Although convection-enhanced delivery has been proposed to bypass the BBB and deliver drugs directly to brain tumors, it remains highly invasive and difficult for targeting disseminated tumors. Therefore, future trials will continue to refine intra-arterial CT as a treatment for gliomas [130–132].

#### 4.4.2. Adjuvant RT

We found that adjuvant RT was associated with improved PFS compared to either salvage RT or no RT in patients with oligodendroglioma. Adjuvant RT is commonly used to treat GBM or glioma, enhancing survival and local control by targeting residual tumor cells. However, it can also cause late adverse effects, including neurocognitive dysfunction, radiation-induced tumors, tissue necrosis, and a decline in health-related quality of life. Future research will focus on developing optimal regimens combining adjuvant RT with other therapies to minimize side effects. Additionally, more advanced RT technologies are needed to improve treatment accuracy.

#### 4.4.3. CRT

We found that CRT, including TMZ or other alkylating CT combined with RT, was associated with improved OS and PFS in glioma and GBM patients. However, a high-quality meta-analysis also showed that TMZ plus RT was linked to more adverse events in GBM patients compared to RT alone.

Currently, the standard treatment for gliomas involves maximal resection followed by concomitant CT with TMZ within 30 days after surgery [133]. CRT, compared to RT or CT alone, can reduce the resistance caused by MGMT, thereby prolonging OS and PFS. However, both CT and RT carry risks of side effects, and their combination may exacerbate these adverse effects, as observed in our findings. Therefore, concurrent CRT with adjuvant TMZ is the preferred treatment for patients aged ≥70 years with good performance status and MGMT promoter methylation [134]. A systematic review and network meta-analysis involving 1596 elderly patients with GBM [135] suggests that the prognosis of moderately hypofractionated RT (3-weeks) with concurrent and adjuvant temozolomide is significantly better than RT alone or TMZ alone, which further proves our findings. This evidence reinforces the reliability of our findings regarding the superiority of combined CRT in improving survival outcomes, particularly in elderly GBM patients. Nevertheless, despite the consistent trends across studies, variations in study design, inclusion criteria, and treatment regimens warrant cautious interpretation. Future studies will investigate combined CRT with immunotherapy and explore the molecular mechanisms underlying the interaction between CRT and gliomas or GBMs to improve treatment efficacy and precision.

### 4.5. Surgery

Most meta-analyses on surgery provide ‘low’ or ‘very low’ quality evidence; however, several outcomes related to VE, iMRI, and awake craniotomy demonstrate moderate quality.

#### 4.5.1. Cerebral ventricular entry (VE) during surgery

Ventricular entry (VE) is often necessary in glioma resection to achieve a wider resection, especially when the tumor is located near the ventricles [136]. We found that cerebral ventricular entry during surgery increased leptomeningeal dissemination and hydrocephalus. One study found that ventricle entry during tumor resection was associated with a significantly higher rate of perioperative complications, with nearly half of the patients developing complications if the ventricle was entered [137]. Previous studies indicated that VE was linked to an increased incidence of leptomeningeal dissemination, likely through CSF circulation of neoplastic cells [136,138]. Furthermore, VE has been associated with higher complication rates and worse overall survival [137]. However, a study on GBM suggested no difference in the incidence of complications, including hydrocephalus, intraventricular hemorrhage, and surgical site infections, between patients who underwent VE and those who did not. Additionally, there was no increase in leptomeningeal spread or distant parenchymal recurrence in patients with VE, supporting its use to maximize gross total resection (EOR) [139].

Future trials will assess the relationship between VE size and patient outcomes in glioma, balancing extensive resection with the avoidance of postoperative neurological deficits that negatively impact function, to achieve personalized resection. Additionally, future studies will focus on the FLAIR hyperintense signal near the ventricular wall, where tumor cells may be present, to achieve wider resection.

#### 4.5.2. iMRI

The primary rationale for using iMRI is to control resection during surgery [140]. Brain shift during surgery reduces the reliability of preoperative MRI for conventional neuronavigation [140]. iMRI can update neuronavigation images, providing a more accurate visualization of brain parenchyma. However, iMRI requires a high installation cost for a dedicated operating room and may increase surgery duration and present technical challenges [56]. We found that iMRI was associated with a higher EOR in glioma patients compared to conventional surgery. A study also reported that iMRI-guided GBM multiforme resection resulted in greater tumor resection, improved quality of life, and prolonged survival compared to conventional neuronavigation-guided surgery [140].

Currently, volumetry is not a valid endpoint due to low interobserver agreement for postoperative and residual tumor volumes. However, future studies may address this by using iMRI to assess residual tumor volume or resection percentage as primary endpoints. Additionally, future investigations will compare iMRI with other image-guided surgery techniques, including intraoperative ultrasound, CT, and fluorescence-guided surgery using 5-aminolevulinic acid, to determine the most effective imaging technology for various surgeries. This will facilitate the use of iMRI in more procedures to enhance the EOR and prolong OS and PFS in malignant gliomas and other tumors. iMRI will also integrate with molecular imaging techniques to improve tumor biology assessment and support more personalized treatment protocols. Furthermore, advancements in iMRI technology are needed to reduce surgery duration and technical challenges, such as patient positioning. Finally, efforts to reduce the high installation cost of iMRI should be prioritized.

#### 4.5.3. Awake craniotomy

Awake craniotomy (AC) is an increasingly popular technique that keeps the patient awake and responsive during tumor excision. Combined with intraoperative cortical and subcortical mapping, AC helps prevent injury to eloquent brain areas [39,141,142]. However, intraoperative mapping using cortical and subcortical electrostimulation during AC may cause varying degrees of neurological deficits [143].

We found that AC was associated with a higher 3-month postoperative seizure freedom rate in patients with eloquent gliomas. However, a study showed that AC for intraaxial tumors and tumors of the supplementary motor area was linked to a higher risk of intraoperative seizures, potentially reducing the rate of GTR, leading to procedure failure and increasing the incidence of short-term postoperative motor and speech deterioration [144,145].

Future research will focus on integrating AC with surgical adjuncts, such as subcortical and cortical mapping, to achieve more reliable cortical localization and provide anatomical insights. Additionally, combining AC with neuroimaging techniques will be explored to gather functional information and improve prognosis. To reduce neurological deficits, future trials will investigate strategies to increase the distance between the point of electrostimulation and vital brain structures [146,147].

### 4.6. Integrating Chinese medicine and Western medicine

We found that integrating Chinese medicine with Western medicine was associated with improved 2-year OSR, greater efficacy, reduced bone marrow suppression and gastrointestinal adverse reactions, and smaller tumor size. Our re-analysis indicated that various Chinese herbal formulas combined with CT or RT benefit glioma patients, including Guben Yiqi Decoction, Jianghuang Xiaoliu Decoction, Shezhi HuangLing Decoction, Naofukang Decoction, Zini Decoction, Shenqi Injection, Gouqiduotang, Tongqiao Huoxue, Naoliu Decoction, and Sanqi Kangliu Decoction. Each herbal formula contains numerous ingredients that enhance immunity, exhibit anti-tumor effects, reduce CT side effects, and improve overall efficacy. A study including 110 patients with glioma by Yibin et al. [148] found that Self-made Yiqi Huoxue decoction combined with nalmefene hydrochloride in patients with qi deficiency and blood stasis syndrome after glioma surgery can effectively relieve clinical symptoms and signs, improve intelligence level and quality of life, and help to up-regulate total superoxide dismutase, catalase, and reduced glutathione concentrations. In conclusion, our findings are consistent with previous studies demonstrating the potential benefits of integrating traditional Chinese medicine with Western therapies in glioma management. The integration of traditional Chinese medicine with conventional therapies has shown potential advantages in supportive care, particularly in mitigating chemotherapy-induced gastrointestinal toxicity and bone marrow suppression. Specific herbal formulations such as Guben Yiqi Decoction and Naofukang Decoction may exert immunomodulatory and anti-inflammatory effects that complement chemoradiotherapy [13]. However, most of the existing literature, including our own, is limited by heterogeneity in study design, insufficient sample sizes, and varying methodological quality. Future studies will investigate additional Chinese medicines showing promise in glioma treatment, along with complementary therapies such as Chinese medicine rehabilitation, acupuncture, and Chinese therapeutic massage, to further improve health outcomes for glioma and GBM patients.

### 4.7. Gene therapy

We indicated that gene therapy was significantly associated with an increase in median survival time for high-grade glioma patients. A meta-analysis by Elnaz et al. [149] found that gene therapy elevated OS and PFS at two-year follow-up. Our findings are largely consistent with these results, further supporting the potential of gene therapy as a promising adjunct in the treatment of high-grade gliomas. Similar improvements in survival outcomes have been reported in multiple studies utilizing different genetic strategies, including oncolytic viruses, tumor-suppressor gene delivery, and RNA-based interventions.

However, despite the encouraging signals, current evidence remains limited by small sample sizes, heterogeneity in gene delivery methods, and lack of long-term follow-up. In our reanalysis, the degree of statistical uncertainty and overlapping confidence intervals also suggest that further confirmation is required.

Future studies should aim to validate these effects in large-scale randomized controlled trials, with standardized outcome reporting and stratification by molecular subtype, to clarify the true clinical utility of gene-based therapies in glioma management.

### 4.8. Strengths and limitations

This study is the first comprehensive umbrella review to systematically compile and evaluate glioma therapies. Two researchers independently conducted searches, selected studies, and extracted data. Where data were sufficient, we recalculated effect sizes with 95% confidence intervals, applied fixed or random effects models, and assessed heterogeneity and publication bias for each meta-analysis. We used AMSTAR for methodological quality, GRADE for evidence strength, and classified outcomes to gauge confidence. Based on these analyses, we offered evidence-based recommendations for optimal glioma therapies.

However, there are limitations to this umbrella review. It is constrained by the number of outcomes that could be assessed, meaning that many therapies for glioma could not be evaluated. Additionally, therapies not included in systematic reviews or meta-analyses were beyond the scope of this review. Therefore, this study is influenced by the original study designs and data quality. Future research should explore the specific mechanisms and long-term effects of certain treatment strategies. Despite efforts to reduce heterogeneity by reanalyzing the data, some endpoints remained unexplained, which may have impacted the conclusions. Furthermore, due to the lack of data, the possibility of publication bias could not be analyzed in individual meta-analyses, resulting in some degree of inaccuracy in the results. In addition, this umbrella review was confined to previously published systematic reviews and meta-analyses and therefore may not reflect insights from recent or small-scale primary studies that have yet to be synthesized. Such omissions could result in an incomplete appraisal of emerging treatment strategies and further publication bias. In summary, significant heterogeneity and bias risk in the source meta-analyses may constrain definitive results. In addition, despite recent advances in glioma research, several important gaps remain that warrant focused attention in future studies. First, certain glioma subtypes-such as oligodendrogliomas, pediatric diffuse gliomas, and rare molecular variants-remain under-investigated in high-level evidence syntheses. These subtypes may exhibit distinct biological behaviors and therapeutic responses that are not adequately captured in broader analyses. Second, few studies have directly compared therapeutic modalities in a head-to-head manner, especially for combinations such as chemoradiotherapy with immunotherapy or targeted agents. Randomized trials specifically designed to evaluate the comparative efficacy and safety of these integrated approaches are essential for guiding clinical decision-making. Third, there is a pressing need to standardize outcome definitions and reporting criteria across studies. Inconsistent endpoints, varying follow-up durations, and non-uniform efficacy metrics substantially hinder evidence synthesis and limit the generalizability of findings. Establishing consensus-based reporting frameworks would enhance comparability between studies and strengthen the quality of future meta-analyses. Addressing these gaps will be instrumental in refining treatment strategies and improving outcomes for diverse glioma patient populations.

## 5. Conclusion

This umbrella review identifies several therapies for gliomas, including immunotherapy, targeted therapies, CRT, surgery, gene therapy, integrated Chinese and Western medicine, and combined regimens, all of which show potential and advancements. High-quality evidence indicates that integrated Chinese and Western medicine, CRT with or without targeted therapy, and targeted therapy with or without CT are associated with improved OS and PFS. However, targeted therapy with or without RT is linked to higher adverse events, while integrated Chinese and Western medicine therapy is associated with fewer adverse events. Moderate-quality evidence supports similar findings, including CRT and targeted therapy with or without CT. Additionally, immunotherapies are linked to improved OS and PFS, whereas alkylating CT is associated with worse PFS. Given the lack of high-quality data, ongoing systematic reviews are imperative as new therapies are developed, particularly immunotherapies and those guided by genetic profiling. Future research should focus on combining therapies and advanced diagnostics to address tumor microenvironment challenges, resistance, and recurrence, while improving treatment precision and minimizing adverse effects. Future studies should also aim to optimize these treatments for broader patient populations and explore personalized therapeutic combinations to enhance efficacy.

## Supplementary Material

PRISMA_2020_checklist.docx

TableS1.docx

TableS3.docx

TableS4.docx

TableS2.docx

## Data Availability

The original contributions presented in the study are included in the article/supplementary material, further inquiries can be directed to the corresponding author.
