# Supplementary material for: Comparative efficacy of glioma treatment strategies: an umbrella review of meta-analyses
Source: Ann Med. 2025 Jul 1;57(1):2525394. doi: 10.1080/07853890.2025.2525394 (PMC12224736; doi:10.1080/07853890.2025.2525394)
Supplement: TableS1.docx [file IANN_A_2525394_SM3282.docx]

TableS1: Literature Search Strategy

PUBMED-1207

****((("Glioma"[Mesh]) OR (((((((((((((Gliomas) OR (Glial Cell Tumors)) OR (Glial Cell Tumor)) OR (Tumor, Glial Cell)) OR (Tumors, Glial Cell)) OR (Mixed Glioma)) OR (Glioma, Mixed)) OR (Gliomas, Mixed)) OR (Mixed Gliomas)) OR (Malignant Glioma)) OR (Glioma, Malignant)) OR (Gliomas, Malignant)) OR (Malignant Gliomas))) AND (systematic review or meta-analysis)) AND (("Therapeutics"[Mesh]) OR (((((Therapeutic) OR (Therapy)) OR (Therapies)) OR (Treatment)) OR (Treatments)))****

****Embase-1939****


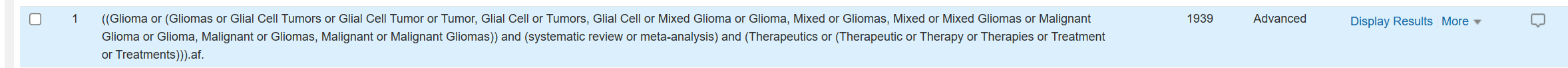


Cochrane-86
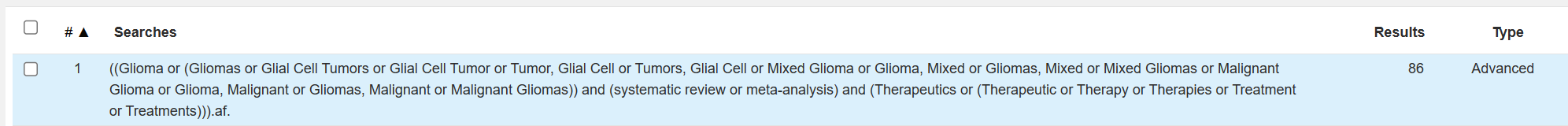


WOS-1011

**((Glioma) OR (((((((((((((Gliomas) OR (Glial Cell Tumors)) OR (Glial Cell Tumor)) OR (Tumor, Glial Cell)) OR (Tumors, Glial Cell)) OR (Mixed Glioma)) OR (Glioma, Mixed)) OR (Gliomas, Mixed)) OR (Mixed Gliomas)) OR (Malignant Glioma)) OR (Glioma, Malignant)) OR (Gliomas, Malignant)) OR (Malignant Gliomas))) AND (systematic review or meta-analysis)) AND ((Therapeutics) OR (((((Therapeutic) OR (Therapy)) OR (Therapies)) OR (Treatment)) OR (Treatments)))** (Topic)
