# Supplementary material for: Comparative efficacy of glioma treatment strategies: an umbrella review of meta-analyses
Source: Ann Med. 2025 Jul 1;57(1):2525394. doi: 10.1080/07853890.2025.2525394 (PMC12224736; doi:10.1080/07853890.2025.2525394)
Supplement: TableS3.docx [file IANN_A_2525394_SM3281.docx]

TableS3: AMSTAR score

| Treatment | Control | Population | Study | A priori design provided | Duplicate study selection & data extraction | At least two electronic databases searched | Status of publication used as an inclusion criterion | List of included and excluded studies provided | Characteristics of included studies provided | Scientific quality of included studies assessed | Scientific quality of the included studies use dappropriately to form conclusions | Appropriate methods to combine studies | Publication bias assessed | Conflict of interest included | Total AMSTAR Score |
| --- | --- | --- | --- | --- | --- | --- | --- | --- | --- | --- | --- | --- | --- | --- | --- |
|  |  |  |  |  |  |  |  |  |  |  |  |  |  |  |  |
|  |  |  |  |  |  |  |  |  |  |  |  |  |  |  |  |
| GTR | STR | Clear Cell Ependymomas | Sheen Kweh 2021 | 0 | 1 | 1 | 0 | 1 | 1 | 1 | 0 | 1 | 0 | 1 | 7 |
| GTR with adjuvant therapy | STR with adjuvant therapy | Clear Cell Ependymomas | Sheen Kweh 2021 | 0 | 1 | 1 | 0 | 1 | 1 | 1 | 0 | 1 | 0 | 1 | 7 |
| RT plus agents of CT | RT | Anaplastic glioma | Zhang 2013 | 0 | 1 | 1 | 0 | 0 | 1 | 0 | 0 | 1 | 1 | 1 | 6 |
| GTR | STR | Pediatric spinal cord astrocytomas | Azad 2018 | 0 | 1 | 1 | 0 | 0 | 1 | 0 | 1 | 1 | 0 | 1 | 6 |
| Surgical resection or CT | RT | Recurrent ependymoma | byer 2019 | 1 | 1 | 1 | 0 | 1 | 1 | 0 | 0 | 1 | 0 | 1 | 7 |
| GTR or STR | Biopsy and decompression | Intramedullary Grade II ependymomas | Sun 2019 | 0 | 1 | 1 | 0 | 1 | 0 | 1 | 0 | 1 | 0 | 1 | 5 |
| Adjuvant therapy or CRT | RT only | Intramedullary Grade II ependymomas | Sun 2019 | 0 | 1 | 1 | 0 | 1 | 0 | 1 | 0 | 1 | 0 | 1 | 5 |
| Awake DES | General anesthesia | Insular gliomas | Di Carlo 2020 | 0 | 1 | 1 | 0 | 1 | 1 | 1 | 0 | 1 | 1 | 1 | 7 |
| HFRT | CFRT | DIPG | Park 2020 | 0 | 1 | 1 | 0 | 1 | 1 | 0 | 0 | 1 | 1 | 1 | 7 |
| Endoscopic third ventriculostomy | Endoscopic third ventriculostomy | DIPG | Guida 2019 | 1 | 1 | 1 | 0 | 1 | 1 | 1 | 0 | 1 | 1 | 1 | 9 |
| GTR or STR or PR | SpTR | Difuse low‑grade glioma | Elsheikh 2022 | 1 | 1 | 1 | 0 | 1 | 1 | 1 | 0 | 1 | 1 | 1 | 9 |
| Resection | Biopsy | Butterfly glioblastoma | Soliman 2022 | 0 | 1 | 1 | 0 | 1 | 1 | 1 | 0 | 1 | 0 | 1 | 7 |
| Surgery or CT | RT | Pediatric Patients With Optic Pathway Gliomas | Thirunavu 2021 | 0 | 1 | 1 | 0 | 1 | 1 | 1 | 0 | 1 | 0 | 1 | 7 |
| Surgery or CT | RT | Optic Pathway Glioma | Yousefi 2022 | 0 | 1 | 1 | 0 | 1 | 0 | 1 | 0 | 1 | 1 | 1 | 7 |
| Intraoperative ALA or fluorescein or ultrasound | IMRI | High-grade gliomas | Eljamel 2016 | 0 | 0 | 1 | 0 | 0 | 0 | 0 | 0 | 1 | 0 | 1 | 3 |
| 5-aminolevulinic acid –guided surgical resection | Conventional microsurgical resection | High-grade gliomas | Gandhi 2019 | 0 | 1 | 1 | 0 | 1 | 0 | 0 | 0 | 1 | 0 | 1 | 5 |
| Awake craniotomy | General anesthesia | High-grade gliomas | Cui 2023 | 0 | 0 | 1 | 0 | 1 | 1 | 0 | 0 | 1 | 0 | 1 | 5 |
| GTR or STR | Biopsy | Elderly patients (≥60 y) with high-grade gliomas | Almenawer 2015 | 0 | 1 | 1 | 0 | 1 | 1 | 1 | 0 | 1 | 1 | 1 | 8 |
| Intraoperative stimulation mappingor awake craniotomy | General anesthesia | High-grade gliomas | Gerritsen 2019 | 0 | 1 | 1 | 0 | 1 | 1 | 0 | 0 | 1 | 1 | 1 | 7 |
| LITT | Open craniotomy | High-grade gliomas | Barnett 2016 | 0 | 1 | 1 | 0 | 1 | 1 | 1 | 0 | 1 | 0 | 1 | 7 |
| Long-term therapy with TMZ (>6 cycles) | Stupp regimen (6 cycles of TMZ) | High-grade gliomas | Xu 2017 | 0 | 1 | 1 | 0 | 1 | 1 | 1 | 0 | 1 | 1 | 1 | 8 |
| Vincristine regimens | Non-vincristine regimens | High-grade gliomas | Aydin 2010 | 0 | 0 | 0 | 0 | 0 | 0 | 0 | 0 | 1 | 0 | 1 | 2 |
| BV plus combined RT and TMZ | RT and TMZ | High-grade gliomas | Fu 2016 | 0 | 1 | 1 | 0 | 0 | 1 | 1 | 0 | 1 | 0 | 1 | 6 |
| DC therapy or viral therapy | Standard therapy | High-grade gliomas | Vatu 2019 | 0 | 1 | 1 | 0 | 0 | 0 | 0 | 0 | 1 | 1 | 1 | 5 |
| DC therapy | Standard therapy | High-grade gliomas | Li 2018 | 0 | 1 | 1 | 0 | 1 | 1 | 1 | 0 | 1 | 1 | 1 | 8 |
| Combination of re-irradiation therapy and BV | Re-irradiation therapy | High-grade gliomas | kulinich 2021 | 0 | 0 | 1 | 0 | 1 | 1 | 1 | 0 | 1 | 0 | 1 | 6 |
| Nitrosurea-based CT plus RT | RT only | High-grade gliomas | Hauch 2005 | 0 | 1 | 1 | 0 | 0 | 0 | 0 | 0 | 1 | 0 | 1 | 4 |
| Viral vector-mediated gene therapy with herpes simplex virus thymidine kinase | Standard treatment | High-grade gliomas or glioblastoma | Zhao 2014 | 0 | 1 | 1 | 0 | 1 | 1 | 0 | 0 | 1 | 0 | 1 | 6 |
| Interstitial therapy | Intracavitary therapy | High-grade gliomas | Choi 2020 | 0 | 1 | 1 | 0 | 1 | 1 | 0 | 1 | 1 | 0 | 1 | 7 |
| High-dose-rate techniques | Low-dose-rate techniques | High-grade gliomas | Choi 2020 | 0 | 1 | 1 | 0 | 1 | 1 | 0 | 1 | 1 | 0 | 1 | 7 |
| Intraoperative implantation of Carmustine Wafers | Stupp Regimen | High-grade gliomas | Ricciardi 2022 | 0 | 1 | 1 | 0 | 1 | 1 | 0 | 0 | 1 | 0 | 1 | 6 |
| Combination of immunotherapy and SOC | SOC alone | High-grade gliomas | Guo 2023 | 1 | 1 | 1 | 0 | 1 | 1 | 1 | 0 | 1 | 1 | 1 | 9 |
| TMZ and RT | RT alone | High-grade gliomas | Hart 2016 | 0 | 1 | 1 | 1 | 1 | 1 | 1 | 0 | 1 | 1 | 1 | 9 |
| TMZ | RT | Elderly patients (≥60 y) with high-grade gliomas | Hart 2016 | 0 | 1 | 1 | 1 | 1 | 1 | 1 | 0 | 1 | 1 | 1 | 9 |
| Dose‐dense TMZ | Metronomic TMZ | High-grade gliomas | Hart 2016 | 0 | 1 | 1 | 1 | 1 | 1 | 1 | 0 | 1 | 1 | 1 | 9 |
| TMZ | Nitrosourea CT | Recurrent glioblastoma | Hart 2016 | 0 | 1 | 1 | 1 | 1 | 1 | 1 | 0 | 1 | 1 | 1 | 9 |
| Etoposide | No etoposide | High-grade gliomas | Leonard 2013 | 0 | 0 | 0 | 0 | 0 | 0 | 0 | 0 | 1 | 0 | 1 | 2 |
| Irinotecan | No irinotecan | High-grade gliomas | Leonard 2013 | 0 | 0 | 0 | 0 | 0 | 0 | 0 | 0 | 1 | 0 | 1 | 2 |
| Reduced-dose BV | Standard -dose BV | Recurrent high-grade glioma or glioblastoma | Chen 2020 | 0 | 1 | 1 | 0 | 1 | 1 | 1 | 0 | 1 | 1 | 1 | 8 |
| Apatinib combined with dose-dense TMZ | Apatinib combined with conventional-dose TMZ | Recurrent high-grade glioma | Li 2023 | 0 | 1 | 1 | 0 | 1 | 1 | 0 | 0 | 1 | 1 | 1 | 7 |
| Schedule of 7 days on or 7 days off for the TMZ treatment or schedule of 21 days on or 7 days off for the TMZ treatment or schedule of 40–50 mg or m2 continuous for the TMZ treatment | Standard TMZ regimen | Recurrent high-grade glioma | Wei 2015 | 0 | 1 | 1 | 0 | 1 | 1 | 0 | 0 | 1 | 1 | 1 | 7 |
| Combination therapy or Reirradiation | Systemic therapy | Recurrent high-grade glioma | Marwah 2023 | 1 | 1 | 1 | 0 | 1 | 1 | 1 | 1 | 1 | 1 | 1 | 10 |
| ReRT with or without non-BV-based systemic therapy | BV-based combination therapy | Recurrent high-grade glioma | Marwah 2023 | 1 | 1 | 1 | 0 | 1 | 1 | 1 | 1 | 1 | 1 | 1 | 10 |
| GTR or STR or Resection of Any Extent | Biopsy | Low-Grade Glioma | Yang 2018 | 0 | 1 | 1 | 0 | 1 | 1 | 1 | 0 | 1 | 1 | 1 | 8 |
| GTR or STR | Biopsy | Low-Grade Glioma | Brown 2019 | 0 | 1 | 1 | 0 | 1 | 0 | 0 | 1 | 1 | 1 | 1 | 7 |
| Early radiation or delayed radiation | No radiation | Low-Grade Glioma | Brown 2019 | 0 | 1 | 1 | 0 | 1 | 0 | 0 | 1 | 1 | 1 | 1 | 7 |
| CT | No CT | Low-Grade Glioma | Brown 2019 | 0 | 1 | 1 | 0 | 1 | 0 | 0 | 1 | 1 | 1 | 1 | 7 |
| Surgical resection or GTR or STR or Adjuvant RT or Adjuvant CT or Multifocal Localization and Surgical Resection | Biopsy | Adult high-grade brainstem gliomas | Lus 2023 | 1 | 1 | 1 | 0 | 1 | 1 | 1 | 0 | 1 | 1 | 1 | 9 |
| Gamma Knife stereotactic radiosurgery | RT | Surgically inaccessible low-grade brainstem gliomas | Gagliardi 2021 | 0 | 1 | 1 | 0 | 1 | 1 | 0 | 0 | 1 | 0 | 1 | 6 |
| Hyperfractionated RT or HFRT | Conventional RT | Diffuse brainstem glioma in children and young adults | Hu 2016 | 0 | 1 | 1 | 1 | 1 | 1 | 1 | 1 | 1 | 1 | 1 | 10 |
| GTR or STR | Biopsy | PHGGs | Hatoum 2022 | 0 | 1 | 1 | 0 | 1 | 1 | 1 | 0 | 1 | 1 | 1 | 8 |
| Targeted therapy or immunotherapy or immunotherapy | Recurrent pediatric high-grade glioma | Recurrent pediatric high-grade glioma | Kline 2018 | 1 | 0 | 1 | 0 | 1 | 1 | 1 | 0 | 1 | 1 | 1 | 8 |
| GTR | STR | Infratentorial ependymomas | Montgomery 2023 | 0 | 1 | 1 | 0 | 1 | 1 | 1 | 0 | 1 | 1 | 1 | 8 |
| CT and radiation therapy or CT alone or CT alone | No CT and radiation therapy | Recurrent infratentorial ependymomas | Montgomery 2023 | 0 | 1 | 1 | 0 | 1 | 1 | 1 | 0 | 1 | 1 | 1 | 8 |
| GTR or STR | No surgery | Infratentorial ependymomas | Montgomery 2023 | 0 | 1 | 1 | 0 | 1 | 1 | 1 | 0 | 1 | 1 | 1 | 8 |
| CT and radiation therapy or CT alone or CT alone | No CT and radiation therapy | Recurrent infratentorial ependymomas | Montgomery 2023 | 0 | 1 | 1 | 0 | 1 | 1 | 1 | 0 | 1 | 1 | 1 | 8 |
| GTR | Supramaxi mal resection | Glioblastoma | Mier-García 2023 | 1 | 1 | 1 | 0 | 1 | 1 | 1 | 1 | 1 | 1 | 1 | 10 |
| GTR | Supramaxi mal resection | IDH wild-type | Mier-García 2023 | 1 | 1 | 1 | 0 | 1 | 1 | 1 | 1 | 1 | 1 | 1 | 10 |
| Surgery or CT or RT | Shunt | Glioblastoma multiforme | Cunha 2019 | 0 | 0 | 1 | 0 | 1 | 0 | 0 | 0 | 1 | 0 | 1 | 4 |
| Diffusion tensor imaging or fMRI or iMRI | Standard navigation | Glioma | Caras 2020 | 0 | 0 | 0 | 0 | 1 | 1 | 0 | 0 | 1 | 0 | 1 | 4 |
| IMRI | Conventional surgery | High-grade gliomas | Lo 2021 | 0 | 1 | 1 | 0 | 1 | 1 | 1 | 0 | 1 | 1 | 1 | 8 |
| IMRI | Conventional surgery | Low-Grade Glioma | Lo 2021 | 0 | 1 | 1 | 0 | 1 | 1 | 1 | 0 | 1 | 1 | 1 | 8 |
| BV plus lomustine or BV monotherapy or Regorafenib or TMZ plus Depatux‐M or Fotemustine or BV and irinotecan | Lomustine | Glioblastoma | Wei 2017 | 0 | 1 | 1 | 0 | 1 | 1 | 1 | 1 | 1 | 1 | 1 | 9 |
| Targeted combined CRT | CRT alone | Glioma | Ma 2023 | 1 | 1 | 1 | 0 | 1 | 1 | 1 | 0 | 1 | 1 | 1 | 9 |
| Charged particle radiation | Photon therapy | Glioma | Al-Lami 2024 | 0 | 0 | 1 | 0 | 1 | 1 | 1 | 0 | 1 | 0 | 1 | 6 |
| Hormone replacement therapy or oral contraceptives | No exogenous hormones use | Glioma | Lan 2018 | 0 | 1 | 1 | 0 | 1 | 1 | 1 | 1 | 1 | 1 | 1 | 9 |
| Active immunotherapy vaccination or Peptide vaccination or DNA vaccines  or Viral vector-based vaccines or Antigen non-specific vaccines or Autologous tumor cell therapy) or Passive immunotherapy(Lymphokine activated killer cell immuno therapy or Antibodies) | Standard care treatments(included surgical resection, RT, or CT) | Glioma | Hanaei 2018 | 0 | 1 | 1 | 0 | 1 | 1 | 1 | 0 | 1 | 0 | 1 | 7 |
| Awake craniotomy | General anesthetic resection | Glioma | Bu 2021 | 0 | 1 | 1 | 0 | 1 | 1 | 1 | 0 | 1 | 1 | 1 | 8 |
| CyberKnife treatment plus CT | CyberKnife treatment | Recurrent malignant gliomas | De Maria 2021 | 0 | 1 | 1 | 0 | 1 | 1 | 1 | 0 | 1 | 0 | 1 | 7 |
| Western medicine | Chinese medicine combined with western medicine treatment | Glioma | Yu 2021 | 0 | 1 | 1 | 0 | 1 | 1 | 1 | 0 | 1 | 1 | 1 | 8 |
| TMZ or other CT | No CT | Gliomatosis Cerebri | Georgakis 2018 | 1 | 1 | 1 | 0 | 0 | 1 | 0 | 0 | 1 | 0 | 1 | 6 |
| Local tumor radiation or Wole brain radiation or unknown method of radiation | No radiation | Gliomatosis Cerebri | Georgakis 2018 | 1 | 1 | 1 | 0 | 0 | 1 | 0 | 0 | 1 | 0 | 1 | 6 |
| Partial resection or Extensive resection | No surgery | Gliomatosis Cerebri | Georgakis 2018 | 1 | 1 | 1 | 0 | 0 | 1 | 0 | 0 | 1 | 0 | 1 | 6 |
| SRS plus BV | SRS alone | Recurrent glioblastoma | Habibi 2024 | 0 | 1 | 1 | 0 | 1 | 1 | 1 | 0 | 1 | 1 | 1 | 8 |
| BV plus other CRT | Other CRT | Recurrent glioblastoma | LI 2016 | 1 | 1 | 1 | 0 | 1 | 1 | 1 | 1 | 1 | 1 | 1 | 10 |
| Active immunotherapy | Standard therapy（combination of surgical resection, RT or CT） | Glioblastoma | Wahyuhadi 2022 | 1 | 1 | 1 | 0 | 1 | 1 | 1 | 0 | 1 | 0 | 1 | 8 |
| Immunotherapy | Non‐immunotherapy | Glioblastoma | Hu 2022 | 1 | 1 | 1 | 1 | 1 | 1 | 1 | 0 | 1 | 1 | 1 | 10 |
| Dose-escalated radiation therapy with or without TMZ or standard-of-care radiation therapy with TMZ | SOC radiation therapy | Newly diagnosed glioblastoma multiforme | Singh 2021 | 0 | 0 | 1 | 0 | 0 | 1 | 0 | 0 | 1 | 1 | 1 | 5 |
| Supratotal resection or STR | GTR | Glioblastoma Multiforme | Aziz 2023 | 1 | 1 | 1 | 0 | 1 | 1 | 1 | 0 | 1 | 1 | 1 | 9 |
| GTR or STR | Biopsy | Newly diagnosed supratentorial glioblastoma multiforme | Brown 2016 | 0 | 1 | 1 | 0 | 1 | 1 | 0 | 1 | 1 | 1 | 1 | 8 |
| Surgical excision | Biopsy | Glioblastoma | Elena 2016 | 0 | 0 | 0 | 0 | 0 | 0 | 0 | 0 | 1 | 0 | 1 | 2 |
| RT or alkylating drugs or “targeted” treatments or RT alone or RT and alkylating CT | No radiation | Glioblastoma | Elena 2016 | 0 | 0 | 0 | 0 | 0 | 0 | 0 | 0 | 1 | 0 | 1 | 2 |
| Levetiracetam | SOC | Glioblastoma | Chen 2022 | 0 | 1 | 1 | 0 | 1 | 1 | 1 | 0 | 1 | 1 | 1 | 8 |
| Levetiracetam with SOC | Other AED | Glioblastoma | Chen 2022 | 0 | 1 | 1 | 0 | 1 | 1 | 1 | 0 | 1 | 1 | 1 | 8 |
| Addition of Tumor Treating Fields therapy to SOC CRT | SOC alone | Newly diagnosed glioblastoma | Ballo 2023 | 0 | 1 | 1 | 0 | 1 | 1 | 1 | 0 | 1 | 0 | 1 | 7 |
| Valproic acid | Non-Valproic acid | Glioblastoma multiforme | Yuan 2014 | 0 | 1 | 1 | 1 | 1 | 1 | 1 | 0 | 1 | 1 | 1 | 9 |
| Valproic acid | Other AED use | Glioblastoma multiforme | Yuan 2014 | 0 | 1 | 1 | 1 | 1 | 1 | 1 | 0 | 1 | 1 | 1 | 9 |
| Valproic acid | No AED treatment | Glioblastoma multiforme | Yuan 2014 | 0 | 1 | 1 | 1 | 1 | 1 | 1 | 0 | 1 | 1 | 1 | 9 |
| Combination of molecularly targeted drugs with TMZ plus RT | TMZ plus RT | Glioblastoma | Wang 2019 | 0 | 1 | 1 | 0 | 1 | 1 | 1 | 0 | 1 | 1 | 1 | 8 |
| Treatment with targeted therapy alone or in combination with another medication (irinotecan or TMZ) | TMZ | Glioblastoma | Scherm 2023 | 0 | 1 | 1 | 0 | 1 | 1 | 0 | 0 | 1 | 1 | 1 | 7 |
| BV or Rindopepimut or Nimotuzumab or Everolimus or Temsirolimus or Cilengitide or Veliparib | TMZ | Glioblastoma | Scherm 2023 | 0 | 1 | 1 | 0 | 1 | 1 | 0 | 0 | 1 | 1 | 1 | 7 |
| Targeted therapy(including Regorafenib or Galunisertib or Depatux-M or Enzastaurin or Cediranib) | Lomustine | Glioblastoma | Ippen 2024 | 0 | 1 | 1 | 0 | 1 | 1 | 0 | 0 | 1 | 1 | 1 | 7 |
| Targeted therapy(including Regorafenib or Galunisertib or Depatux-M or Enzastaurin or Cediranib) plus Lomustine | Lomustine | Glioblastoma | Ippen 2024 | 0 | 1 | 1 | 0 | 1 | 1 | 0 | 0 | 1 | 1 | 1 | 7 |
| Targeted therapy(including Regorafenib or Galunisertib or Depatux-M or Enzastaurin or Cediranib) | BV | Glioblastoma | Ippen 2024 | 0 | 1 | 1 | 0 | 1 | 1 | 0 | 0 | 1 | 1 | 1 | 7 |
| BV combined with TMZ | TMZ alone | Glioma or glioblastoma | Wei 2024 | 0 | 1 | 1 | 0 | 1 | 1 | 1 | 0 | 1 | 1 | 1 | 8 |
| Molecularly targeted drugs combined with TMZ plus RT | TMZ plus RT | MGMT-methylated glioblastoma | Su 2016 | 0 | 1 | 1 | 0 | 1 | 1 | 1 | 0 | 1 | 1 | 1 | 8 |
| Molecularly targeted drugs combined with TMZ plus RT | TMZ plus RT | MGMT-methylated glioblastoma | Su 2016 | 0 | 1 | 1 | 0 | 1 | 1 | 1 | 0 | 1 | 1 | 1 | 8 |
| BV combined with TMZ plus RT | TMZ plus RT | Glioblastoma | Su 2016 | 0 | 1 | 1 | 0 | 1 | 1 | 1 | 0 | 1 | 1 | 1 | 8 |
| Cilengitide combined with TMZ plus RT | TMZ plus RT | Glioblastoma | Su 2016 | 0 | 1 | 1 | 0 | 1 | 1 | 1 | 0 | 1 | 1 | 1 | 8 |
| Molecularly targeted drugs combined with TMZ plus RT | TMZ plus RT | Glioblastoma | Su 2016 | 0 | 1 | 1 | 0 | 1 | 1 | 1 | 0 | 1 | 1 | 1 | 8 |
| Standard therapy + Anti-vascular endothelial growth factor | Standard therapy alone | Glioblastoma | Xiao 2018 | 0 | 1 | 1 | 0 | 1 | 1 | 1 | 0 | 1 | 0 | 1 | 7 |
| Standard therapy + BV | Standard therapy alone | Glioblastoma | Xiao 2018 | 0 | 1 | 1 | 0 | 1 | 1 | 1 | 0 | 1 | 0 | 1 | 7 |
| TMZ plus carmustine CT | TMZ | Glioblastoma | Xiao 2020 | 0 | 1 | 1 | 0 | 1 | 1 | 1 | 0 | 1 | 1 | 1 | 8 |
| Carmustine was administered by intravenous injection | Carmustine implanted into the resection cavity | Glioblastoma | Xiao 2020 | 0 | 1 | 1 | 0 | 1 | 1 | 1 | 0 | 1 | 1 | 1 | 8 |
| Higher dose of carmustine | Lower dose of carmustine | Glioblastoma | Xiao 2020 | 0 | 1 | 1 | 0 | 1 | 1 | 1 | 0 | 1 | 1 | 1 | 8 |
| Carmustine | Non-carmustine-treated | Glioblastoma | Xiao 2020 | 0 | 1 | 1 | 0 | 1 | 1 | 1 | 0 | 1 | 1 | 1 | 8 |
| Molecularly targeted therapies with RT | TMZ plus RT | Newly diagnosed glioblastoma multiforme | dos Santos 2015 | 0 | 1 | 1 | 0 | 1 | 1 | 0 | 0 | 1 | 0 | 1 | 6 |
| BV alone or BV combined with Lomustine or Cediranib alone or Cediranib combined with TMZ | Lomustine alone | Glioblastoma | Lombardi 2017 | 0 | 1 | 1 | 1 | 1 | 1 | 0 | 0 | 1 | 1 | 1 | 8 |
| BV plus Irinotecan combined with TMZ plus RT as first-line treatment or Dasatinib and RT combined with TMZ or Temsirolimus and RT combined with TMZ as first-line treatment | TMZ combined with RT | Glioblastoma | Lombardi 2017 | 0 | 1 | 1 | 1 | 1 | 1 | 0 | 0 | 1 | 1 | 1 | 8 |
| Cilengitide plus TMZ | TMZ combined with RT | MGMT-nonmethylated glioblastoma | Lombardi 2017 | 0 | 1 | 1 | 1 | 1 | 1 | 0 | 0 | 1 | 1 | 1 | 8 |
| Enzastaurin alone as second-line treatment | Lomustine alone as second-line treatment | Glioblastoma | Lombardi 2017 | 0 | 1 | 1 | 1 | 1 | 1 | 0 | 0 | 1 | 1 | 1 | 8 |
| BV as first-line treatment | BV as second-line treatment | Glioblastoma | Lombardi 2017 | 0 | 1 | 1 | 1 | 1 | 1 | 0 | 0 | 1 | 1 | 1 | 8 |
| Antiangiogenic drugs alone or Antiangiogenic drugs combined with cytotoxic drug | Cytotoxic drug | Glioblastoma | Lombardi 2017 | 0 | 1 | 1 | 1 | 1 | 1 | 0 | 0 | 1 | 1 | 1 | 8 |
| BV | Other angiogenesis inhibitors | Recurrent glioblastoma | Wang 2016 | 0 | 1 | 1 | 0 | 1 | 1 | 1 | 0 | 1 | 1 | 1 | 8 |
| BV | Thalidomide | Recurrent glioblastoma | Wang 2016 | 0 | 1 | 1 | 0 | 1 | 1 | 1 | 0 | 1 | 1 | 1 | 8 |
| Fluorescent-light use | Standard white-light use | Glioblastoma | Nikova 2022 | 0 | 1 | 1 | 0 | 1 | 0 | 0 | 0 | 1 | 0 | 1 | 5 |
| BV | Specific cytotoxic treatments, including lomustine, fotemustine, nivolumab, or TMZ | Recurrent glioblastoma | Zhang 2021 | 0 | 1 | 1 | 0 | 1 | 1 | 1 | 0 | 1 | 0 | 1 | 7 |
| BV plus some specific cytotoxic treatments | A certain cytotoxic treatment | Recurrent glioblastoma | Zhang 2021 | 0 | 1 | 1 | 0 | 1 | 1 | 1 | 0 | 1 | 0 | 1 | 7 |
| BV in combination with CRT(including TMZ or Lomustine) | CRT alone(including TMZ or Lomustine) | Glioblastoma | Lan 2022 | 0 | 1 | 1 | 0 | 1 | 1 | 1 | 0 | 1 | 0 | 1 | 7 |
| Combination of BV with CT(including carboplatin or iriontecan or lomustin) | BV or CT(including TMZ or Lomustine) | Glioblastoma | Yang 2017 | 1 | 1 | 1 | 0 | 1 | 1 | 1 | 0 | 1 | 0 | 1 | 8 |
| Extended adjuvant TMZ | Standard Stupp protocol | Glioblastoma | Attarian 2021 | 0 | 1 | 1 | 0 | 1 | 1 | 1 | 0 | 1 | 0 | 1 | 7 |
| High cumulative dose TMZ | Normal cumulative dose TMZ | Glioblastoma | Sun 2015 | 0 | 1 | 1 | 0 | 1 | 1 | 1 | 0 | 1 | 1 | 1 | 8 |
| Higher peak concentration TMZ | lower peak TMZ | Glioblastoma | Sun 2015 | 0 | 1 | 1 | 0 | 1 | 1 | 1 | 0 | 1 | 1 | 1 | 8 |
| Actinomycin-D or vincristine or mitoxantrone or vinblastine or doxorubicin or diaziquone or cisplatin or methotrexate or cytasine arabinoside or 5-flurouracil or bleomycin or carboplatin or carmustine or nimustine | Lomustine | Glioblastoma | Wolff 1999 | 0 | 0 | 0 | 0 | 0 | 0 | 0 | 0 | 1 | 0 | 1 | 2 |
| CT | No CT | Glioblastoma | Spiegel 2007 | 0 | 1 | 1 | 0 | 1 | 1 | 1 | 0 | 1 | 1 | 1 | 8 |
| Multi-agent CT | Single-agent CT | Glioblastoma | Spiegel 2007 | 0 | 1 | 1 | 0 | 1 | 1 | 1 | 0 | 1 | 1 | 1 | 8 |
| HFRT | Conventional fraction RT | Glioblastoma multiforme | Liao 2019 | 0 | 1 | 1 | 0 | 1 | 1 | 1 | 0 | 1 | 1 | 1 | 8 |
| RT plus oral CT | RT alone | Glioblastoma | Wang 2017 | 0 | 1 | 1 | 0 | 1 | 1 | 1 | 0 | 1 | 0 | 1 | 7 |
| RT plus TMZ | RT alone | Glioblastoma | Zhao 2021 | 0 | 1 | 1 | 0 | 1 | 1 | 1 | 0 | 1 | 0 | 1 | 7 |
| HFRT | 3D conformal normo-fractionated RT | Glioblastoma multiforme | Trone 2020 | 0 | 1 | 1 | 0 | 1 | 1 | 0 | 0 | 1 | 0 | 1 | 6 |
| HFRT plus TMZ CT | HFRT | Glioblastoma multiforme | Trone 2020 | 0 | 1 | 1 | 0 | 1 | 1 | 0 | 0 | 1 | 0 | 1 | 6 |
| Non-stereotactic HFRT or hypofractionated stereotactic RT | Stupp trial | Glioblastoma multiforme | Trone 2020 | 0 | 1 | 1 | 0 | 1 | 1 | 0 | 0 | 1 | 0 | 1 | 6 |
| HFRT or non-stereotactic hypofractionation or hypofractionated stereotactic RT or normofractionnated RT plus stereotactic radiation | “exclusive radiation” Stupp protocol | Glioblastoma multiforme | Trone 2020 | 0 | 1 | 1 | 0 | 1 | 1 | 0 | 0 | 1 | 0 | 1 | 6 |
| Stereotactic radiosurgery | Stupp protocol | Glioblastoma multiforme | Trone 2020 | 0 | 1 | 1 | 0 | 1 | 1 | 0 | 0 | 1 | 0 | 1 | 6 |
| HFRT | Normo-fractionation | Glioblastoma multiforme | Trone 2020 | 0 | 1 | 1 | 0 | 1 | 1 | 0 | 0 | 1 | 0 | 1 | 6 |
| Anti‐EGFR monoclonal antibodies with or without CT or EGFR TKIs with or without CT or anti‐EGFR vaccine with or without SOC | Placebo or SOC with or without CT | Glioblastoma | Lee 2020 | 0 | 1 | 1 | 0 | 1 | 1 | 1 | 1 | 1 | 1 | 1 | 9 |
| Combining immunotherapy with SOC chemo-RT following surgical resection | SOC CRT | Newly diagnosed glioblastoma | Lara-Velazquez 2021 | 0 | 1 | 1 | 0 | 1 | 1 | 1 | 1 | 1 | 1 | 1 | 9 |
| Aggressive resection | CT | Pediatric brain and or or spine glioblastoma multiforme | Yang 2021 | 0 | 1 | 1 | 0 | 1 | 1 | 0 | 0 | 1 | 0 | 1 | 6 |
| STR plus CT or biopsy plus CT or STR | Biopsy | Pediatric brain and or or spine glioblastoma multiforme | Yang 2021 | 0 | 1 | 1 | 0 | 1 | 1 | 0 | 0 | 1 | 0 | 1 | 6 |
| Repeat surgery | No surgery at recurrence | Recurrent glioblastoma diagnoses | Lu 2018 | 0 | 1 | 1 | 0 | 1 | 1 | 1 | 0 | 1 | 1 | 1 | 8 |
| BV plus other CRT | Other CRT | New diagnosed glioblastoma multiforme | Li 2015 | 0 | 1 | 1 | 0 | 1 | 1 | 1 | 0 | 1 | 1 | 1 | 8 |
| Combination of BV with irinotecan | BV alone | Glioblastoma multiforme | Zhang 2012 | 0 | 1 | 1 | 0 | 1 | 1 | 0 | 0 | 1 | 0 | 1 | 6 |
| High dose metronomic TMZ schedule | Low dose metronomic TMZ schedules | Recurrent glioblastoma multiforme | Chen 2013 | 0 | 1 | 1 | 0 | 1 | 1 | 0 | 0 | 1 | 0 | 1 | 6 |
| Metronomic TMZ schedules | Standard TMZ schedules | Recurrent glioblastoma multiforme | Chen 2013 | 0 | 1 | 1 | 0 | 1 | 1 | 0 | 0 | 1 | 0 | 1 | 6 |
| TMZ with hypofractionated radiation therapy | TMZ with standard radiation therapy | Elderly glioblastoma | Lu 2019 | 0 | 1 | 1 | 0 | 1 | 1 | 1 | 1 | 1 | 1 | 1 | 9 |
| TMZ alone | RT alone | Elderly glioblastoma | Yin 2014 | 0 | 1 | 1 | 0 | 1 | 1 | 1 | 0 | 1 | 1 | 1 | 8 |
| Hypofractionated radiation therapy | Conventionally fractionated radiation therapy | IDH-wildtype glioblastoma | Chidley 2022 | 0 | 0 | 0 | 0 | 1 | 1 | 0 | 0 | 1 | 0 | 1 | 4 |
| GTR | STR or biopsy | Glioblastoma IDH-WT | Jusue-Torres 2023 | 0 | 1 | 1 | 0 | 1 | 1 | 1 | 1 | 1 | 1 | 1 | 9 |
| Intra-arterial CT | Intravenous CT | Malignant gliomas | Chen 2013 | 0 | 1 | 1 | 0 | 1 | 1 | 1 | 1 | 1 | 0 | 1 | 8 |
| Radiation therapy and adjuvant CT | Radiation therapy alone | Anaplastic astrocytoma or glioblastoma multiforme | Fine 1993 | 0 | 0 | 0 | 0 | 0 | 0 | 0 | 0 | 1 | 1 | 1 | 3 |
| Hyperfractionated RT | Conventional fractionation RT | Malignant glioma | Laperriere 2002 | 0 | 0 | 1 | 0 | 0 | 1 | 0 | 0 | 1 | 0 | 1 | 4 |
| Whole brain irradiation | Local field boost irradiation | Malignant glioma | Laperriere 2002 | 0 | 0 | 1 | 0 | 0 | 1 | 0 | 0 | 1 | 0 | 1 | 4 |
| 60 Gy in 30 fractions | 45 Gy in 20 fractions | Malignant glioma | Laperriere 2002 | 0 | 0 | 1 | 0 | 0 | 1 | 0 | 0 | 1 | 0 | 1 | 4 |
| Particle therapy | Photon RT | Malignant glioma | Laperriere 2002 | 0 | 0 | 1 | 0 | 0 | 1 | 0 | 0 | 1 | 0 | 1 | 4 |
| Radiation sensitizers | RT alone | Malignant glioma | Laperriere 2002 | 0 | 0 | 1 | 0 | 0 | 1 | 0 | 0 | 1 | 0 | 1 | 4 |
| Vaccines | Conventional treatments | Malignant glioma | Jajin 2024 | 0 | 1 | 1 | 0 | 1 | 1 | 1 | 0 | 1 | 1 | 1 | 8 |
| Personalized vaccines | Non-personalized vaccines | Malignant glioma | Jajin 2024 | 0 | 1 | 1 | 0 | 1 | 1 | 1 | 0 | 1 | 1 | 1 | 8 |
| Cytoreductive resection | Biopsy | Supratentorial high-grade glioma | Tsitlakidis 2010 | 0 | 1 | 1 | 1 | 1 | 1 | 0 | 0 | 1 | 1 | 1 | 8 |
| Intraoperative 5-aminolevulinic acid (5-ALA)-guided resection | Conventional neuronavigation-guided resection | High-grade malignant gliomas | Zhao 2013 | 0 | 1 | 1 | 0 | 1 | 1 | 1 | 0 | 1 | 1 | 1 | 8 |
| HFSRT + CT | Sole HFSRT management | Recurrent malignant glioma | Hu 2019 | 0 | 1 | 1 | 0 | 1 | 1 | 0 | 0 | 1 | 1 | 1 | 7 |
| Awake surgery | General anesthesia | Supratentorial right-sided gliomas | Ramirez-Ferrer 2024 | 1 | 1 | 1 | 0 | 1 | 1 | 1 | 0 | 1 | 0 | 1 | 8 |
| Adjuvant RT | Received either salvage RT or no RT at all | Oligodendroglioma | Ng 2024 | 1 | 1 | 1 | 0 | 1 | 1 | 1 | 0 | 1 | 1 | 1 | 9 |
| Adjuvant RT | Salvage RT | Oligodendroglioma | Ng 2024 | 1 | 1 | 1 | 0 | 1 | 1 | 1 | 0 | 1 | 1 | 1 | 9 |
| Adjuvant RT or adjuvant CRT | Adjuvant CT alone | Oligodendroglioma | Ng 2024 | 1 | 1 | 1 | 0 | 1 | 1 | 1 | 0 | 1 | 1 | 1 | 9 |
| Adding CT to RT | Monotherapy with RT alone | Oligodendroglioma | Ng 2024 | 1 | 1 | 1 | 0 | 1 | 1 | 1 | 0 | 1 | 1 | 1 | 9 |
| Glioma resections with stimulation mapping | Glioma resections without stimulation mapping | Infiltrative gliomas | De Witt Hamer 2012 | 0 | 1 | 1 | 0 | 1 | 0 | 1 | 0 | 1 | 1 | 1 | 7 |
| GTR or STR or near total resection | Biopsy and RT | Adult Pilocytic Astrocytoma | Bond 2018 | 0 | 0 | 0 | 0 | 1 | 1 | 0 | 0 | 1 | 0 | 1 | 4 |
| GTR | STR | Grade Ⅱ Pleomorphic Xanthoastrocytoma | Mallick 2018 | 0 | 0 | 1 | 0 | 1 | 0 | 0 | 0 | 1 | 0 | 1 | 4 |
| GTR | Lesser volumes of tumor resected | Intramedullary ependymoma and astrocytoma | Hamilton 2019 | 0 | 1 | 1 | 0 | 0 | 0 | 1 | 0 | 1 | 0 | 1 | 5 |
| RT | No RT | Intramedullary ependymoma and astrocytoma | Hamilton 2019 | 0 | 1 | 1 | 0 | 0 | 0 | 1 | 0 | 1 | 0 | 1 | 5 |
| CRT | RT alone | High-grade glioma | Stewart 2002 | 0 | 1 | 1 | 1 | 0 | 1 | 0 | 0 | 1 | 1 | 1 | 7 |
| Surgery + chemotherapeutic wafer + RT | Surgery + or - placebo wafers + RT + or - systemic CT e.g. PCV or TMZ | High grade glioma | Hart 2008 | 0 | 1 | 1 | 1 | 0 | 1 | 1 | 0 | 1 | 1 | 1 | 8 |
| With ventricular entry during surgery | Without ventricular entry during surgery | High grade glioma | Mistry 2018 | 0 | 1 | 1 | 0 | 0 | 0 | 1 | 1 | 1 | 1 | 1 | 7 |
| Fluorescein-guided surgery | Standard resection without fluorescein guidance | High grade glioma | Smith 2021 | 0 | 1 | 1 | 0 | 1 | 1 | 1 | 0 | 1 | 1 | 1 | 8 |
| Neutron beam therapy | Photon therapy | High grade glioma | Maucort-Boulch 2010 | 0 | 1 | 1 | 0 | 0 | 1 | 1 | 0 | 1 | 1 | 1 | 7 |
| Photon-based intensity-modulated radiation therapy | Proton beam therapy | Pediatric low grade glioma | Lu 2020 | 0 | 1 | 1 | 0 | 1 | 1 | 1 | 1 | 1 | 1 | 1 | 9 |
| Photon-based intensity-modulated radiation therapy | Proton beam therapy | Adult low grade glioma | Lu 2020 | 0 | 1 | 1 | 0 | 1 | 1 | 1 | 1 | 1 | 1 | 1 | 9 |
| GTR | STR | Glioma | Shi 2023 | 0 | 1 | 1 | 0 | 1 | 1 | 1 | 0 | 1 | 1 | 1 | 8 |
| Lobectomy | GTR | Glioblastoma multiforme | Zheng 2023 | 0 | 0 | 1 | 0 | 1 | 1 | 1 | 0 | 1 | 0 | 1 | 6 |
| Awake craniotomy | Asleep craniotomy | Eloquent glioma | Sattari 2024 | 0 | 1 | 1 | 0 | 1 | 1 | 1 | 0 | 1 | 1 | 1 | 8 |
| CRT | RT | *IDH-wild-type gliomas* | Kinslow 2024 | 0 | 1 | 1 | 0 | 0 | 1 | 0 | 0 | 1 | 1 | 1 | 6 |
| Anti-PD-1 or PD-L1 treatment | Placebo with RT and TMZ | Glioma | Zeng 2023 | 1 | 1 | 1 | 0 | 1 | 1 | 1 | 0 | 1 | 1 | 1 | 9 |
| GTR, gross total resection; STR, subtotal resection; CRT, chemoradiotherpy; RT, RT; CT, CT; DES, direct electrical stimulation; PR, partial resection; SpTR, supratotal resection; HFSRT, hypofractionated stereotactic radiotherapy; SOC, standard of care; BV, bevacizumab; TMZ, temozolomide; AED, anti-epileptic drug; EGFR, epidermal growth factor receptor; HFRT, hypofractionated radiotherapy; CFRT, conventional fractionated radiotherapy; DIPG, diffuse intrinsic pontine gliomas; pHGGs, infratentorial pediatric high-grade gliomas; iMRI, intraoperative magnetic resonance imaging; fMRI, functional magnetic reso nance imaging. | | | | | | | | | | | | | | | |
