# Supplementary material for: Comparative efficacy of glioma treatment strategies: an umbrella review of meta-analyses
Source: Ann Med. 2025 Jul 1;57(1):2525394. doi: 10.1080/07853890.2025.2525394 (PMC12224736; doi:10.1080/07853890.2025.2525394)
Supplement: TableS4.docx [file IANN_A_2525394_SM3280.docx]

TableS4: GRADE classification

| Treatment | Control | Population | Outcome | Included MA | No. of studies | Risk of bias | Inconsistency | Indirectness | Imprecision | Publication bias | Plausible confounding | Magnitude of effect | Dose-response gradient | Quality |
| --- | --- | --- | --- | --- | --- | --- | --- | --- | --- | --- | --- | --- | --- | --- |
|  |  |  |  |  |  |  |  |  |  |  |  |  |  |  |
| *Significant associations* | | | | | | | | | | | | | | |
| CRT | RT alone | Anaplastic glioma | OS | Zhang 2013 | 4 | no serious risk | no serious inconsistency | no serious indirectness | serious imprecision | undetected | would not reduce effect | no | no | Moderate |
| CRT | RT alone | Anaplastic glioma | PFS | Zhang 2013 | 4 | no serious risk | no serious inconsistency | no serious indirectness | serious imprecision | undetected | would not reduce effect | no | no | Moderate |
| GTR | STR | Adult Pilocytic Astrocytoma | Tumor recurrence | Bond 2018 | 7 | no serious risk | no serious inconsistency | no serious indirectness | no serious imprecision | strongly suspected | would not reduce effect | yes | no | Low |
| Resection | Biopsy | Butterfly glioblastoma | OS | Soliman 2022 | 13 | no serious risk | serious inconsistency | no serious indirectness | serious imprecision | NA | would not reduce effect | yes | no | Very low |
| 5-aminolevulinic acid (5-ALA)–guided surgical resection | Conventional microsurgical resection | High-grade gliomas | GTR | Gandhi 2019 | 4 | serious risk | no serious inconsistency | no serious indirectness | no serious imprecision | NA | would not reduce effect | yes | no | Low |
| 5-aminolevulinic acid (5-ALA)–guided surgical resection | Conventional microsurgical resection | High-grade gliomas | Mean difference in OS | Gandhi 2019 | 7 | serious risk | no serious inconsistency | no serious indirectness | no serious imprecision | NA | would not reduce effect | no | no | Very low |
| 5-aminolevulinic acid (5-ALA)–guided surgical resection | Conventional microsurgical resection | High-grade gliomas | PFS | Gandhi 2019 | 4 | serious risk | serious inconsistency | no serious indirectness | no serious imprecision | NA | would not reduce effect | no | no | Very low |
| Resection | Biopsy | Elderly patients (≥60 y) with high-grade gliomas | OS | Almenawer 2015 | 9 | no serious risk | serious inconsistency | no serious indirectness | no serious imprecision | undetected | would not reduce effect | no | no | Very low |
| Resection | Biopsy | Elderly patients (≥60 y) with high-grade gliomas | Postoperative KPS | Almenawer 2015 | 4 | no serious risk | serious inconsistency | no serious indirectness | no serious imprecision | undetected | would not reduce effect | no | no | Very low |
| Resection | Biopsy | Elderly patients (≥60 y) with high-grade gliomas | PFS | Almenawer 2015 | 4 | no serious risk | no serious inconsistency | no serious indirectness | no serious imprecision | undetected | would not reduce effect | no | no | Low |
| Resection | Biopsy | Elderly patients (≥60 y) with high-grade gliomas | Mortality | Almenawer 2015 | 8 | no serious risk | no serious inconsistency | no serious indirectness | serious imprecision | undetected | would not reduce effect | yes | no | Low |
| STR | Biopsy | Elderly patients (≥60 y) with high-grade gliomas | OS | Almenawer 2015 | 11 | no serious risk | serious inconsistency | no serious indirectness | serious imprecision | undetected | would not reduce effect | no | no | Very low |
| GTR | Biopsy | Elderly patients (≥60 y) with high-grade gliomas | OS | Almenawer 2015 | 10 | no serious risk | serious inconsistency | no serious indirectness | serious imprecision | undetected | would not reduce effect | no | no | Very low |
| GTR | STR | Elderly patients (≥60 y) with high-grade gliomas | OS | Almenawer 2015 | 11 | no serious risk | no serious inconsistency | no serious indirectness | no serious imprecision | strongly suspected | would not reduce effect | no | no | Very low |
| Stupp regimen (6 cycles of TMZ) | Long-term therapy with TMZ (>6 cycles) | High-grade gliomas | OS | Xu 2017 | 6 | no serious risk | no serious inconsistency | no serious indirectness | no serious imprecision | undetected | would not reduce effect | yes | no | Moderate |
| Stupp regimen (6 cycles of TMZ) | Long-term therapy with TMZ (>6 cycles) | High-grade gliomas | PFS | Xu 2017 | 4 | no serious risk | no serious inconsistency | no serious indirectness | no serious imprecision | undetected | would not reduce effect | yes | no | Moderate |
| Stupp regimen (6 cycles of TMZ) | Long-term therapy with TMZ (>6 cycles) | High-grade gliomas | Side-effects | Xu 2017 | 5 | no serious risk | no serious inconsistency | no serious indirectness | no serious imprecision | undetected | would not reduce effect | yes | no | Moderate |
| BV plus RT/TMZ | RT/TMZ | High-grade gliomas | PFS | Fu 2016 | 3 | no serious risk | serious inconsistency | no serious indirectness | no serious imprecision | NA | would not reduce effect | no | no | Moderate |
| DC vaccination | Standard therapy | High-grade gliomas | OS | Vatu 2019 | 8 | no serious risk | no serious inconsistency | no serious indirectness | no serious imprecision | undetected | would not reduce effect | no | no | Low |
| DC vaccination | Standard therapy | High-grade gliomas | PFS | Vatu 2019 | 3 | no serious risk | no serious inconsistency | no serious indirectness | serious imprecision | undetected | would not reduce effect | no | no | Very low |
| Viral therapy | Standard therapy | High-grade gliomas | OS | Vatu 2019 | 6 | no serious risk | serious inconsistency | no serious indirectness | serious imprecision | undetected | would not reduce effect | no | no | Very low |
| DC vaccination | Control therapy | High-grade gliomas | 1-years OS | Li 2018 | 13 | no serious risk | no serious inconsistency | no serious indirectness | serious imprecision | undetected | would not reduce effect | no | no | very low |
| DC vaccination | Control therapy | High-grade gliomas | 2-years OS | Li 2018 | 12 | no serious risk | no serious inconsistency | no serious indirectness | no serious imprecision | undetected | would not reduce effect | no | no | Low |
| DC vaccination | Control therapy | High-grade gliomas | 3-years OS | Li 2018 | 10 | no serious risk | no serious inconsistency | no serious indirectness | no serious imprecision | undetected | would not reduce effect | yes | no | Moderate |
| DC vaccination | Control therapy | High-grade gliomas | 4-years OS | Li 2018 | 9 | no serious risk | no serious inconsistency | no serious indirectness | no serious imprecision | undetected | would not reduce effect | yes | no | Moderate |
| DC vaccination | Control therapy | High-grade gliomas | 5-years OS | Li 2018 | 6 | no serious risk | no serious inconsistency | no serious indirectness | no serious imprecision | undetected | would not reduce effect | yes | no | Moderate |
| DC vaccination | Control therapy | High-grade gliomas | 2-years PFS | Li 2018 | 4 | no serious risk | no serious inconsistency | no serious indirectness | no serious imprecision | undetected | would not reduce effect | yes | no | Moderate |
| DC vaccination | Control therapy | High-grade gliomas | 3-years PFS | Li 2018 | 3 | no serious risk | no serious inconsistency | no serious indirectness | serious imprecision | undetected | would not reduce effect | yes | no | Low |
| DC vaccination | Control therapy | High-grade gliomas | 4-years PFS | Li 2018 | 2 | no serious risk | no serious inconsistency | no serious indirectness | serious imprecision | undetected | would not reduce effect | yes | no | Low |
| CRT | RT alone | High-grade gliomas | PFS | Stewart 2002 | 8 | no serious risk | NA | no serious indirectness | no serious imprecision | NA | would not reduce effect | no | no | Moderate |
| Gene therapy | Standard treatment | High-grade gliomas | Median survival time | Zhao 2014 | 3 | no serious risk | no serious inconsistency | no serious indirectness | no serious imprecision | NA | would not reduce effect | no | no | High |
| Carmustine Wafers | Stupp Regimen | High-grade gliomas | OS | Ricciardi 2022 | 4 | serious risk | no serious inconsistency | no serious indirectness | serious imprecision | NA | would not reduce effect | no | no | Low |
| Combination of immunotherapy and SOC | SOC alone | High-grade gliomas | OS | Guo 2023 | 11 | serious risk | serious inconsistency | no serious indirectness | serious imprecision | undetected | would not reduce effect | no | no | Very low |
| Combination of immunotherapy and SOC | SOC alone | High-grade gliomas | PFS | Guo 2023 | 6 | serious risk | serious inconsistency | no serious indirectness | serious imprecision | undetected | would not reduce effect | no | no | Very low |
| Combination of immunotherapy and SOC | SOC alone | High-grade gliomas | Adverse events | Guo 2023 | 75 | serious risk | serious inconsistency | no serious indirectness | no serious imprecision | undetected | would not reduce effect | no | no | Very low |
| Combination of DC therapy and SOC | SOC alone | High-grade gliomas | OS | Guo 2023 | 3 | serious risk | no serious inconsistency | no serious indirectness | serious imprecision | undetected | would not reduce effect | no | no | Very low |
| Combination of viral therapy and SOC | SOC alone | High-grade gliomas | Adverse events | Guo 2023 | 45 | serious risk | no serious inconsistency | no serious indirectness | no serious imprecision | strongly suspected | would not reduce effect | no | no | Very low |
| Combination of immunopotentiators and SOC | SOC alone | High-grade gliomas | Adverse events | Guo 2023 | 29 | serious risk | serious inconsistency | no serious indirectness | no serious imprecision | strongly suspected | would not reduce effect | yes | no | Very low |
| Viral therapy with optimized injection methods combined with SOC | SOC alone | High-grade gliomas | OS | Guo 2023 | 4 | serious risk | no serious inconsistency | no serious indirectness | no serious imprecision | undetected | would not reduce effect | no | no | Low |
| Ventricular entry during surgery | Without ventricular entry during surgery | High-grade gliomas | Incidences of leptomeningeal dissemination | Mistry 2018 | 9 | no serious risk | no serious inconsistency | no serious indirectness | no serious imprecision | undetected | would not reduce effect | yes | no | Moderate |
| Ventricular entry during surgery | Without ventricular entry during surgery | High-grade gliomas | Incidences of hydrocephalus | Mistry 2018 | 11 | no serious risk | no serious inconsistency | no serious indirectness | no serious imprecision | undetected | would not reduce effect | yes | no | Moderate |
| TMZ and RT | RT alone | High-grade gliomas | OS | Hart 2016 | 3 | no serious risk | serious inconsistency | no serious indirectness | no serious imprecision | undetected | would not reduce effect | no | no | Moderate |
| TMZ and RT | RT alone | High-grade gliomas | PFS | Hart 2016 | 3 | no serious risk | serious inconsistency | no serious indirectness | serious imprecision | undetected | would not reduce effect | no | no | Low |
| TMZ | Standard CT | High-grade gliomas | Adverse events | Hart 2016 | 5 | no serious risk | serious inconsistency | no serious indirectness | no serious imprecision | undetected | would not reduce effect | yes | no | High |
| TMZ | RT alone | Elderly patients (≥60 y) with high-grade gliomas | Adverse Events | Hart 2016 | 17 | no serious risk | serious inconsistency | no serious indirectness | no serious imprecision | undetected | would not reduce effect | yes | no | High |
| Dose‐dense TMZ | Metronomic TMZ high-grade gliomas | High-grade gliomas | Adverse events | Hart 2016 | 6 | no serious risk | serious inconsistency | no serious indirectness | serious imprecision | undetected | would not reduce effect | yes | no | Moderate |
| Fluorescein-guided surgery | Standard resection without fluorescein guidance | High-grade gliomas | GTR | Smith 2021 | 10 | serious risk | no serious inconsistency | no serious indirectness | no serious imprecision | undetected | would not reduce effect | yes | no | Low |
| Combination therapy | Systemic therapy | Recurrent high-grade glioma | OS | Marwah 2023 | 7 | no serious risk | no serious inconsistency | no serious indirectness | serious imprecision | undetected | would not reduce effect | no | no | Very low |
| Combination therapy | Systemic therapy | Recurrent high-grade glioma | PFS | Marwah 2023 | 5 | no serious risk | serious inconsistency | no serious indirectness | no serious imprecision | undetected | would not reduce effect | no | no | Very low |
| Combination therapy | Reirradiation | Recurrent high-grade glioma | OS | Marwah 2023 | 7 | no serious risk | no serious inconsistency | no serious indirectness | serious imprecision | undetected | would not reduce effect | no | no | Very low |
| Combination therapy | Reirradiation | Recurrent high-grade glioma | PFS | Marwah 2023 | 4 | no serious risk | no serious inconsistency | no serious indirectness | no serious imprecision | undetected | would not reduce effect | no | no | Low |
| BV-based combination therapy | Reirradiation with/without non-BV-based systemic therapy | Recurrent high-grade glioma | OS | Marwah 2023 | 5 | no serious risk | no serious inconsistency | no serious indirectness | serious imprecision | strongly suspected | would not reduce effect | yes | no | Very low |
| BV-based combination therapy | Reirradiation with/without non-BV-based systemic therapy | Recurrent high-grade glioma | PFS | Marwah 2023 | 2 | no serious risk | no serious inconsistency | no serious indirectness | serious imprecision | strongly suspected | would not reduce effect | yes | no | Very low |
| BV-based combination therapy | Reirradiation with/without non-BV-based systemic therapy | Recurrent high-grade glioma | Toxicity-Radiation Necrosis | Marwah 2023 | 5 | no serious risk | no serious inconsistency | no serious indirectness | no serious imprecision | strongly suspected | would not reduce effect | yes | no | Low |
| BV-Based Combination Therapy | Systemic Therapy | Recurrent high-grade glioma | OS | Marwah 2023 | 6 | no serious risk | no serious inconsistency | no serious indirectness | serious imprecision | undetected | would not reduce effect | no | no | Very low |
| BV-Based Combination Therapy | Systemic Therapy | Recurrent high-grade glioma | PFS | Marwah 2023 | 4 | no serious risk | serious inconsistency | no serious indirectness | serious imprecision | undetected | would not reduce effect | no | no | Very low |
| Reirradiation | Systemic therapy | Recurrent glioblastoma | OS | Marwah 2023 | 3 | no serious risk | no serious inconsistency | no serious indirectness | serious imprecision | undetected | would not reduce effect | no | no | Very low |
| Reirradiation | Systemic therapy | Recurrent glioblastoma | PFS | Marwah 2023 | 2 | no serious risk | no serious inconsistency | no serious indirectness | serious imprecision | undetected | would not reduce effect | no | no | Very low |
| Combination therapy | Systemic therapy | Recurrent glioblastoma | OS | Marwah 2023 | 2 | no serious risk | no serious inconsistency | no serious indirectness | serious imprecision | undetected | would not reduce effect | no | no | Very low |
| Combination therapy | Systemic therapy | Recurrent glioblastoma | PFS | Marwah 2023 | 2 | no serious risk | no serious inconsistency | no serious indirectness | serious imprecision | undetected | would not reduce effect | no | no | Very low |
| Combination Therapy | Reirradiation | Recurrent glioblastoma | OS | Marwah 2023 | 5 | no serious risk | no serious inconsistency | no serious indirectness | no serious imprecision | undetected | would not reduce effect | no | no | Low |
| Combination Therapy | Reirradiation | Recurrent glioblastoma | PFS | Marwah 2023 | 4 | no serious risk | no serious inconsistency | no serious indirectness | no serious imprecision | undetected | would not reduce effect | no | no | Low |
| BV-based combination therapy | Reirradiation with/without non-BV-based systemic therapy | Recurrent glioblastoma | OS | Marwah 2023 | 4 | no serious risk | no serious inconsistency | no serious indirectness | no serious imprecision | undetected | would not reduce effect | yes | no | Moderate |
| BV-based combination therapy | Reirradiation with/without non-BV-based systemic therapy | Recurrent glioblastoma | PFS | Marwah 2023 | 2 | no serious risk | no serious inconsistency | no serious indirectness | serious imprecision | undetected | would not reduce effect | yes | no | Low |
| STR | Biopsy | Low-Grade Glioma | OS | Yang 2018 | 3 | no serious risk | serious inconsistency | no serious indirectness | serious imprecision | undetected | would not reduce effect | no | no | Very low |
| GTR | Biopsy | Low-Grade Glioma | OS | Yang 2018 | 4 | no serious risk | no serious inconsistency | no serious indirectness | no serious imprecision | undetected | would not reduce effect | no | no | Low |
| Resection of any extent | Biopsy | Low-Grade Glioma | OS | Yang 2018 | 6 | no serious risk | no serious inconsistency | no serious indirectness | serious imprecision | undetected | would not reduce effect | no | no | Very low |
| STR | GTR | Low-Grade Glioma | OS | Yang 2018 | 11 | no serious risk | serious inconsistency | no serious indirectness | no serious imprecision | undetected | would not reduce effect | no | no | Very low |
| GTR | STR | Low-Grade Glioma | 5-year survival | Yang 2018 | 14 | no serious risk | no serious inconsistency | no serious indirectness | no serious imprecision | undetected | would not reduce effect | no | no | low |
| GTR | STR | Low-Grade Glioma | PFS | Yang 2018 | 5 | no serious risk | serious inconsistency | no serious indirectness | serious imprecision | undetected | would not reduce effect | no | no | Very low |
| GTR | STR | Low-Grade Glioma | Seizure control | Yang 2018 | 6 | no serious risk | no serious inconsistency | no serious indirectness | no serious imprecision | undetected | would not reduce effect | no | no | Low |
| GTR | STR | Low-Grade Glioma | Malignant transformation | Yang 2018 | 6 | no serious risk | no serious inconsistency | no serious indirectness | serious imprecision | undetected | would not reduce effect | yes | no | Low |
| GTR | STR | Low-Grade Glioma | Mortality at 2 years | Brown 2019 | 19 | serious risk | no serious inconsistency | no serious indirectness | no serious imprecision | NA | would not reduce effect | yes | no | Low |
| GTR | STR | Low-Grade Glioma | Mortality at 5 years | Brown 2019 | 23 | serious risk | no serious inconsistency | no serious indirectness | no serious imprecision | NA | would not reduce effect | yes | no | Low |
| GTR | STR | Low-Grade Glioma | Mortality at 10 years | Brown 2019 | 15 | serious risk | serious inconsistency | no serious indirectness | no serious imprecision | NA | would not reduce effect | yes | no | Very low |
| GTR | STR | Low-Grade Glioma | Progression at 2 years | Brown 2019 | 10 | serious risk | serious inconsistency | no serious indirectness | no serious imprecision | NA | would not reduce effect | yes | no | Very low |
| GTR | STR | Low-Grade Glioma | Progression at 5 years | Brown 2019 | 13 | serious risk | serious inconsistency | no serious indirectness | no serious imprecision | NA | would not reduce effect | no | no | Very low |
| GTR | STR | Low-Grade Glioma | Progression at 10 years | Brown 2019 | 8 | serious risk | serious inconsistency | no serious indirectness | no serious imprecision | NA | would not reduce effect | no | no | Very low |
| STR | Biopsy | Low-Grade Glioma | Mortality at 2 years | Brown 2019 | 7 | serious risk | no serious inconsistency | no serious indirectness | serious imprecision | NA | would not reduce effect | no | no | Very low |
| Resection | Biopsy | Low-Grade Glioma | Mortality at 2 years | Brown 2019 | 7 | serious risk | serious inconsistency | no serious indirectness | serious imprecision | NA | would not reduce effect | yes | no | Very low |
| Resection | Biopsy | Low-Grade Glioma | Mortality at 5 years | Brown 2019 | 8 | serious risk | serious inconsistency | no serious indirectness | serious imprecision | NA | would not reduce effect | no | no | Very low |
| Resection | Biopsy | Low-Grade Glioma | Mortality at 10 years | Brown 2019 | 3 | serious risk | no serious inconsistency | no serious indirectness | serious imprecision | NA | would not reduce effect | no | no | Very low |
| Early Radiation | Late/No Radiation | Low-Grade Glioma | Progression at 2 years | Brown 2019 | 6 | serious risk | serious inconsistency | no serious indirectness | serious imprecision | NA | would not reduce effect | no | no | Very low |
| Early Radiation | Late/No Radiation | Low-Grade Glioma | Progression at 5 years | Brown 2019 | 6 | serious risk | serious inconsistency | no serious indirectness | no serious imprecision | NA | would not reduce effect | no | no | Very low |
| Early Radiation | Late/No Radiation | Low-Grade Glioma | Progression at 10 years | Brown 2019 | 4 | serious risk | serious inconsistency | no serious indirectness | serious imprecision | NA | would not reduce effect | no | no | Very low |
| CT | RT | Low-Grade Glioma | Progression at 5 years | Brown 2019 | 3 | serious risk | no serious inconsistency | no serious indirectness | serious imprecision | NA | would not reduce effect | no | no | Very low |
| CT | RT | Low-Grade Glioma | Progression at 10 years | Brown 2019 | 3 | serious risk | serious inconsistency | no serious indirectness | serious imprecision | NA | would not reduce effect | no | no | Very low |
| GTR | STR | pHGGs | 1-Year Mortality | Hatoum 2022 | 29 | no serious risk | no serious inconsistency | no serious indirectness | no serious imprecision | undetected | would not reduce effect | no | no | Low |
| GTR | STR | pHGGs | 2-Year Mortality | Hatoum 2022 | 29 | no serious risk | no serious inconsistency | no serious indirectness | no serious imprecision | undetected | would not reduce effect | no | no | Low |
| STR | Biopsy | pHGGs | 2-Year Mortality | Hatoum 2022 | 23 | no serious risk | no serious inconsistency | no serious indirectness | serious imprecision | undetected | would not reduce effect | no | no | Very low |
| GTR | STR | pHGGs | 6-Month Progression | Hatoum 2022 | 17 | no serious risk | no serious inconsistency | no serious indirectness | serious imprecision | undetected | would not reduce effect | no | no | Very low |
| GTR | STR | pHGGs | 1-Year Progression | Hatoum 2022 | 17 | no serious risk | no serious inconsistency | no serious indirectness | no serious imprecision | undetected | would not reduce effect | no | no | Low |
| SMR | GTR | Glioblastoma | PFS | Mier-García 2023 | 8 | serious risk | no serious inconsistency | no serious indirectness | no serious imprecision | NA | would not reduce effect | no | no | Very low |
| SMR | GTR | Glioblastoma | OS | Mier-García 2023 | 13 | serious risk | no serious inconsistency | no serious indirectness | no serious imprecision | NA | would not reduce effect | no | no | Very low |
| SMR | GTR | Glioblastoma, IDH wild-type | PFS | Mier-García 2023 | 3 | serious risk | no serious inconsistency | no serious indirectness | serious imprecision | NA | would not reduce effect | no | no | Very low |
| GTR | STR | Glioma | 1-year OS | Shi 2023 | 13 | no serious risk | serious inconsistency | no serious indirectness | no serious imprecision | undetected | would not reduce effect | no | no | Very low |
| GTR | STR | Glioma | 2-year OS | Shi 2023 | 14 | no serious risk | serious inconsistency | no serious indirectness | serious imprecision | undetected | would not reduce effect | no | no | Very low |
| GTR | STR | Glioma | 3-year OS | Shi 2023 | 10 | no serious risk | no serious inconsistency | no serious indirectness | no serious imprecision | undetected | would not reduce effect | no | no | Low |
| GTR | STR | Glioma | 5-year OS | Shi 2023 | 35 | no serious risk | serious inconsistency | no serious indirectness | no serious imprecision | undetected | would not reduce effect | no | no | Very low |
| GTR | STR | Glioma | 10-year OS | Shi 2023 | 15 | no serious risk | no serious inconsistency | no serious indirectness | no serious imprecision | undetected | would not reduce effect | no | no | Low |
| GTR | STR | Glioma | 15-year OS | Shi 2023 | 3 | no serious risk | no serious inconsistency | no serious indirectness | no serious imprecision | undetected | would not reduce effect | no | no | Low |
| GTR | STR | Glioma | OS | Shi 2023 | 3 | no serious risk | serious inconsistency | no serious indirectness | serious imprecision | undetected | would not reduce effect | no | no | Very low |
| GTR | STR | Glioma | 1-year PFS | Shi 2023 | 3 | no serious risk | serious inconsistency | no serious indirectness | serious imprecision | undetected | would not reduce effect | no | no | Very low |
| GTR | STR | Glioma | 3-year PFS | Shi 2023 | 3 | no serious risk | no serious inconsistency | no serious indirectness | serious imprecision | undetected | would not reduce effect | yes | no | Very low |
| GTR | STR | Glioma | 5-year PFS | Shi 2023 | 13 | no serious risk | serious inconsistency | no serious indirectness | no serious imprecision | undetected | would not reduce effect | no | no | Very low |
| GTR | STR | Glioma | Recurrence | Shi 2023 | 13 | no serious risk | serious inconsistency | no serious indirectness | no serious imprecision | undetected | would not reduce effect | no | no | Very low |
| GTR | STR | Glioma | 5-year local control | Shi 2023 | 2 | no serious risk | no serious inconsistency | no serious indirectness | serious imprecision | undetected | would not reduce effect | yes | no | Low |
| GTR | STR | Glioma | Seizure control | Shi 2023 | 4 | no serious risk | serious inconsistency | no serious indirectness | serious imprecision | undetected | would not reduce effect | no | no | Very low |
| GTR | STR | High-grade gliomas | 1-year OS | Shi 2023 | 12 | no serious risk | serious inconsistency | no serious indirectness | no serious imprecision | undetected | would not reduce effect | no | no | Very low |
| GTR | STR | Adults with low grade glioma | 1-year OS | Shi 2023 | 9 | no serious risk | serious inconsistency | no serious indirectness | serious imprecision | undetected | would not reduce effect | no | no | Very low |
| GTR | STR | High-grade gliomas | 2-year OS | Shi 2023 | 11 | no serious risk | no serious inconsistency | no serious indirectness | no serious imprecision | undetected | would not reduce effect | no | no | Low |
| GTR | STR | Adults with low grade glioma | 2-year OS | Shi 2023 | 2 | no serious risk | no serious inconsistency | no serious indirectness | serious imprecision | undetected | would not reduce effect | no | no | Very low |
| GTR | STR | High-grade gliomas | 3-year OS | Shi 2023 | 5 | no serious risk | no serious inconsistency | no serious indirectness | serious imprecision | undetected | would not reduce effect | no | no | Very low |
| GTR | STR | Adults with low grade glioma | 3-year OS | Shi 2023 | 4 | no serious risk | no serious inconsistency | no serious indirectness | serious imprecision | undetected | would not reduce effect | no | no | Very low |
| GTR | STR | Low-grade glioma | 5-year OS | Shi 2023 | 13 | no serious risk | serious inconsistency | no serious indirectness | serious imprecision | undetected | would not reduce effect | no | no | Very low |
| GTR | STR | High-grade gliomas | OS | Shi 2023 | 2 | no serious risk | serious inconsistency | no serious indirectness | serious imprecision | undetected | would not reduce effect | no | no | Very low |
| iMRI | Conventional surgery | Glioma | GTR | Lo 2021 | 11 | no serious risk | no serious inconsistency | no serious indirectness | serious imprecision | undetected | would not reduce effect | no | no | Very low |
| iMRI | Conventional surgery | Glioma | Mean extent of resection | Lo 2021 | 9 | no serious risk | no serious inconsistency | no serious indirectness | serious imprecision | undetected | would not reduce effect | no | no | Very low |
| Conventional surgery | iMRI | Glioma | GTR | Lo 2021 | 3 | no serious risk | no serious inconsistency | no serious indirectness | serious imprecision | undetected | would not reduce effect | no | no | Moderate |
| Hormone replacement therapy | RT and TMZ | Glioma | Cumulative risk | Lan 2018 | 10 | no serious risk | no serious inconsistency | no serious indirectness | NA | undetected | would not reduce effect | no | no | Very low |
| Oral contraceptives | RT and TMZ | Glioma | Cumulative risk | Lan 2018 | 4 | no serious risk | no serious inconsistency | no serious indirectness | NA | undetected | would not reduce effect | no | no | Very low |
| Immunotherapy | Standard care treatments(included surgical resection, RT, or CT) | Glioma | 1-year OS | Hanaei 2018 | 7 | serious risk | no serious inconsistency | no serious indirectness | serious imprecision | NA | would not reduce effect | no | no | Very low |
| Immunotherapy | Standard care treatments(included surgical resection, RT, or CT) | Glioma | 2-year OS | Hanaei 2018 | 8 | serious risk | no serious inconsistency | no serious indirectness | serious imprecision | NA | would not reduce effect | no | no | Very low |
| Active immunotherapy | Standard care treatments(included surgical resection, RT, or CT) | Glioma | 3-year OS | Hanaei 2018 | 3 | serious risk | no serious inconsistency | no serious indirectness | serious imprecision | NA | would not reduce effect | no | no | Very low |
| Immunotherapy | Standard care treatments(included surgical resection, RT, or CT) | Glioma | PFS | Hanaei 2018 | 9 | serious risk | NA | no serious indirectness | serious imprecision | NA | would not reduce effect | no | no | Very low |
| Awake craniotomy | General anesthetic resection | Glioma | Late neurological deficit | Bu 2021 | 9 | no serious risk | no serious inconsistency | no serious indirectness | serious imprecision | undetected | would not reduce effect | no | no | Very low |
| Integrated Traditional Chinese and Western medicine | Western medical treatment | Glioma | Efficacy | Yu 2021 | 8 | no serious risk | no serious inconsistency | no serious indirectness | no serious imprecision | undetected | would not reduce effect | no | no | High |
| Integrated Traditional Chinese and Western medicine | Western medical treatment | Glioma | Complete remission/partial remission/stable disease/progress disease | Yu 2021 | 5 | no serious risk | no serious inconsistency | no serious indirectness | serious imprecision | undetected | would not reduce effect | yes | no | Moderate |
| Integrated Traditional Chinese and Western medicine | Western medical treatment | Glioma | Bone marrow suppression | Yu 2021 | 7 | no serious risk | no serious inconsistency | no serious indirectness | no serious imprecision | undetected | would not reduce effect | yes | no | High |
| Integrated Traditional Chinese and Western medicine | Western medical treatment | Glioma | Gastrointestinal adverse reactions | Yu 2021 | 7 | no serious risk | no serious inconsistency | no serious indirectness | no serious imprecision | undetected | would not reduce effect | yes | no | High |
| Integrated Traditional Chinese and Western medicine | Western medical treatment | Glioma | Two-year survival time | Yu 2021 | 4 | no serious risk | no serious inconsistency | no serious indirectness | no serious imprecision | undetected | would not reduce effect | no | no | High |
| Integrated Traditional Chinese and Western medicine | Western medical treatment | Glioma | Three-year survival time | Yu 2021 | 3 | no serious risk | no serious inconsistency | no serious indirectness | no serious imprecision | undetected | would not reduce effect | yes | no | High |
| Targeted therapy(including Regorafenib or Galunisertib or Depatux-M or Enzastaurin or Cediranib) plus Lomustine | Lomustine | Glioblastoma | PFS | Ippen 2024 | 4 | no serious risk | no serious inconsistency | no serious indirectness | no serious imprecision | undetected | would not reduce effect | no | no | High |
| Targeted therapy | TMZ | Glioblastoma | PFS | Scherm 2023 | 12 | no serious risk | serious inconsistency | no serious indirectness | serious imprecision | undetected | would not reduce effect | no | no | Low |
| Molecularly targeted drugs | TMZ plus RT | Glioblastoma | PFS | Wang 2019 | 13 | no serious risk | serious inconsistency | no serious indirectness | serious imprecision | undetected | would not reduce effect | no | no | Low |
| Molecularly targeted drugs | TMZ plus RT | Glioblastoma | Adverse events | Wang 2019 | 7 | no serious risk | no serious inconsistency | no serious indirectness | no serious imprecision | undetected | would not reduce effect | no | no | High |
| Antiangiogenic drugs | Cytotoxic drug alone | Glioblastoma | PFS | Lombardi 2017 | 15 | no serious risk | serious inconsistency | no serious indirectness | no serious imprecision | undetected | would not reduce effect | no | no | Moderate |
| Carmustine implantation | Non-carmustine-treated | Glioblastoma | OS | Xiao 2020 | 21 | no serious risk | no serious inconsistency | no serious indirectness | no serious imprecision | undetected | would not reduce effect | no | no | Low |
| Carmustine implantation | Non-carmustine-treated | Glioblastoma | PFS | Xiao 2020 | 8 | no serious risk | serious inconsistency | no serious indirectness | serious imprecision | undetected | would not reduce effect | no | no | Very low |
| Carmustine implantation | Non-carmustine-treated | Different pathological grades of glioma | OS | Xiao 2020 | 20 | no serious risk | no serious inconsistency | no serious indirectness | no serious imprecision | undetected | would not reduce effect | no | no | Low |
| Carmustine implantation | TMZ CT and TMZ plus carmustine CT | Glioblastoma | OS | Xiao 2020 | 2 | no serious risk | no serious inconsistency | no serious indirectness | serious imprecision | undetected | would not reduce effect | no | no | Very low |
| Standard therapy + Anti-vascular endothelial growth factor | Standard therapy | Glioblastoma | PFS | Xiao 2018 | 5 | no serious risk | no serious inconsistency | no serious indirectness | no serious imprecision | NA | would not reduce effect | no | no | High |
| Standard therapy + BV | Standard therapy | Glioblastoma | PFS | Xiao 2018 | 3 | no serious risk | no serious inconsistency | no serious indirectness | no serious imprecision | NA | would not reduce effect | no | no | High |
| Molecularly targeted drugs combined with TMZ plus RT | TMZ plus RT | Glioblastoma | PFS | Su 2016 | 7 | no serious risk | no serious inconsistency | no serious indirectness | no serious imprecision | undetected | would not reduce effect | no | no | High |
| Molecularly targeted drugs combined with TMZ plus RT | TMZ plus RT | MGMT-nonmethylated patients | PFS | Su 2016 | 3 | no serious risk | no serious inconsistency | no serious indirectness | no serious imprecision | undetected | would not reduce effect | no | no | High |
| BV combined with TMZ plus RT | TMZ plus RT | Glioblastoma | OS | Su 2016 | 2 | no serious risk | serious inconsistency | no serious indirectness | no serious imprecision | undetected | would not reduce effect | no | no | Moderate |
| Cilengitide | TMZ plus RT | Glioblastoma | PFS | Su 2016 | 3 | no serious risk | no serious inconsistency | no serious indirectness | serious imprecision | undetected | would not reduce effect | no | no | Moderate |
| Molecularly targeted drugs combined with TMZ plus RT | TMZ plus RT | Glioblastoma | Adverse effects | Su 2016 | 7 | no serious risk | no serious inconsistency | no serious indirectness | no serious imprecision | undetected | would not reduce effect | no | no | High |
| BV combined with TMZ | TMZ alone | Glioma or glioblastoma | PFS | Wei 2024 | 7 | no serious risk | no serious inconsistency | no serious indirectness | no serious imprecision | strongly suspected | would not reduce effect | no | no | Moderate |
| BV combined with TMZ | TMZ alone | Glioma or glioblastoma | Overall complete remission rate | Wei 2024 | 2 | no serious risk | no serious inconsistency | no serious indirectness | no serious imprecision | strongly suspected | would not reduce effect | yes | no | High |
| BV combined with TMZ | TMZ alone | Glioma or glioblastoma | OS | Wei 2024 | 7 | no serious risk | serious inconsistency | no serious indirectness | serious imprecision | strongly suspected | would not reduce effect | no | no | Very low |
| BV combined with TMZ | TMZ alone | Glioma or glioblastoma | Adverse events | Wei 2024 | 6 | no serious risk | serious inconsistency | no serious indirectness | no serious imprecision | strongly suspected | would not reduce effect | yes | no | Moderate |
| Levetiracetam plus SOC | SOC | Glioblastoma | OS | Chen 2022 | 6 | no serious risk | serious inconsistency | no serious indirectness | serious imprecision | strongly suspected | would not reduce effect | no | no | Very low |
| Supratotal resection | GTR | Glioblastoma | OS | Aziz 2023 | 11 | no serious risk | serious inconsistency | no serious indirectness | no serious imprecision | strongly suspected | would not reduce effect | no | no | Very low |
| Supratotal resection | GTR | Glioblastoma | PFS | Aziz 2023 | 4 | no serious risk | serious inconsistency | no serious indirectness | serious imprecision | strongly suspected | would not reduce effect | yes | no | Very low |
| Supratotal resection | STR or GTR | Glioblastoma | PFS | Aziz 2023 | 6 | no serious risk | serious inconsistency | no serious indirectness | no serious imprecision | strongly suspected | would not reduce effect | no | no | Very low |
| Tumor Treating Fields therapy plus SOC | SOC alone | Newly diagnosed glioblastoma | OS | Ballo 2023 | 7 | no serious risk | no serious inconsistency | no serious indirectness | no serious imprecision | NA | would not reduce effect | no | no | Low |
| ≥ 75% device usage rate threshold | <75% device usage rate threshold | Newly diagnosed glioblastoma | OS | Ballo 2023 | 5 | no serious risk | no serious inconsistency | no serious indirectness | no serious imprecision | NA | would not reduce effect | no | no | Low |
| BV in combination with CRT(including TMZ or Lomustine) | CRT alone(including TMZ or Lomustine) | Glioblastoma | PFS | Lan 2022 | 7 | no serious risk | serious inconsistency | no serious indirectness | no serious imprecision | undetected | would not reduce effect | no | no | Moderate |
| Combination therapy of BV plus CT | BV or CT alone | Glioblastoma | PFS | Yang 2017 | 4 | no serious risk | no serious inconsistency | no serious indirectness | no serious imprecision | strongly suspected | would not reduce effect | no | no | Moderate |
| Combination therapy of BV plus CT | BV or CT alone | Glioblastoma | Object response rate | Yang 2017 | 3 | no serious risk | no serious inconsistency | no serious indirectness | serious imprecision | strongly suspected | would not reduce effect | no | no | Low |
| Combination therapy of BV plus CT | BV or CT alone | Glioblastoma | Adverse events | Yang 2017 | 2 | no serious risk | no serious inconsistency | no serious indirectness | serious imprecision | strongly suspected | would not reduce effect | no | no | Low |
| CRT | RT | Glioblastoma | OS | Wang 2017 | 5 | no serious risk | serious inconsistency | no serious indirectness | no serious imprecision | strongly suspected | would not reduce effect | no | no | Low |
| RT plus TMZ | RT alone | Glioblastoma | Survival rate | Zhao 2021 | 3 | no serious risk | no serious inconsistency | no serious indirectness | no serious imprecision | NA | would not reduce effect | no | no | High |
| RT plus TMZ | RT alone | Glioblastoma | PFS | Zhao 2021 | 2 | no serious risk | no serious inconsistency | no serious indirectness | no serious imprecision | NA | would not reduce effect | yes | no | High |
| RT plus TMZ | RT alone | Glioblastoma | Adverse events | Zhao 2021 | 2 | no serious risk | no serious inconsistency | no serious indirectness | serious imprecision | NA | would not reduce effect | yes | no | High |
| Anti-EGFR therapies with CT | SOC with CT | Recurrent glioblastoma | PFS | Lee 2020 | 3 | no serious risk | no serious inconsistency | no serious indirectness | serious imprecision | undetected | would not reduce effect | no | no | Moderate |
| GTR | STR | Glioblastoma | 1-Year Mortality | Brown 2016 | 25 | no serious risk | serious inconsistency | no serious indirectness | no serious imprecision | strongly suspected | would not reduce effect | no | no | Very low |
| GTR | STR | Glioblastoma | 2-Year Mortality | Brown 2016 | 23 | no serious risk | serious inconsistency | no serious indirectness | no serious imprecision | undetected | would not reduce effect | no | no | Very low |
| STR | Biopsy | Glioblastoma | 1-Year Mortality | Brown 2016 | 20 | no serious risk | serious inconsistency | no serious indirectness | no serious imprecision | undetected | would not reduce effect | no | no | Very low |
| Resection | Biopsy | Glioblastoma | 1-Year Mortality | Brown 2016 | 21 | no serious risk | serious inconsistency | no serious indirectness | no serious imprecision | undetected | would not reduce effect | no | no | Very low |
| GTR | STR | Glioblastoma | Progression at one year | Brown 2016 | 5 | no serious risk | serious inconsistency | no serious indirectness | no serious imprecision | undetected | would not reduce effect | no | no | Very low |
| Resection | Biopsy | Glioblastoma | Progression at one year | Brown 2016 | 7 | no serious risk | serious inconsistency | no serious indirectness | serious imprecision | undetected | would not reduce effect | no | no | Very low |
| BV plus other CRT | Other CRT | Recurrent glioblastoma | Median PFS | LI 2016 | 5 | no serious risk | serious inconsistency | no serious indirectness | no serious imprecision | undetected | would not reduce effect | no | no | Moderate |
| BV plus other CRT | Other CRT | Recurrent glioblastoma | PFS rate | LI 2016 | 22 | no serious risk | serious inconsistency | no serious indirectness | no serious imprecision | undetected | would not reduce effect | yes | no | High |
| BV plus other CRT | Other CRT | Recurrent glioblastoma | OS rate | LI 2016 | 23 | no serious risk | no serious inconsistency | no serious indirectness | serious imprecision | undetected | would not reduce effect | no | no | High |
| BV plus some specific cytotoxic treatments | A certain cytotoxic treatment | Recurrent glioblastoma | Objective response rate | Zhang 2021 | 3 | no serious risk | no serious inconsistency | no serious indirectness | no serious imprecision | undetected | would not reduce effect | yes | no | High |
| BV plus some specific cytotoxic treatments | A certain cytotoxic treatment | Recurrent glioblastoma | Median PFS | Zhang 2021 | 4 | no serious risk | no serious inconsistency | no serious indirectness | serious imprecision | undetected | would not reduce effect | no | no | Moderate |
| BV plus some specific cytotoxic treatments | A certain cytotoxic treatment | Recurrent glioblastoma | Hypertension | Zhang 2021 | 5 | no serious risk | serious inconsistency | no serious indirectness | serious imprecision | undetected | would not reduce effect | yes | no | Moderate |
| TMZ with hypofractionated radiation therapy | TMZ with standard radiation therapy | Elderly glioblastoma | Median OS | Lu 2019 | 7 | serious risk | serious inconsistency | no serious indirectness | serious imprecision | undetected | would not reduce effect | no | no | Very low |
| TMZ alone | RT alone | Elderly glioblastoma | Methylated tumors | Yin 2014 | 3 | no serious risk | no serious inconsistency | no serious indirectness | serious imprecision | undetected | would not reduce effect | no | no | Very low |
| TMZ alone | RT alone | Elderly glioblastoma | Unmethylated tumors | Yin 2014 | 2 | no serious risk | no serious inconsistency | no serious indirectness | no serious imprecision | undetected | would not reduce effect | no | no | Very low |
| GTR | STR | Glioblastoma, IDH–wild-type | OS | Jusue-Torres 2023 | 5 | serious risk | no serious inconsistency | no serious indirectness | no serious imprecision | undetected | would not reduce effect | no | no | Very low |
| GTR | Biopsy | Glioblastoma, IDH–wild-type | OS | Jusue-Torres 2023 | 2 | serious risk | serious inconsistency | no serious indirectness | NA | undetected | would not reduce effect | no | no | Very low |
| STR | Biopsy | Glioblastoma, IDH–wild-type | OS | Jusue-Torres 2023 | 2 | serious risk | no serious inconsistency | no serious indirectness | NA | undetected | would not reduce effect | no | no | Very low |
| GTR | STR | Glioblastoma, IDH–wild-type | PFS | Jusue-Torres 2023 | 4 | serious risk | serious inconsistency | no serious indirectness | no serious imprecision | strongly suspected | would not reduce effect | no | no | Very low |
| GTR | STR | Glioblastoma, IDH–wild-type | OS | Jusue-Torres 2023 | 4 | serious risk | no serious inconsistency | no serious indirectness | NA | undetected | would not reduce effect | yes | no | Very low |
| GTR | Biopsy | Glioblastoma, IDH–wild-type | OS | Jusue-Torres 2023 | 5 | serious risk | no serious inconsistency | no serious indirectness | no serious imprecision | undetected | would not reduce effect | yes | no | Low |
| GTR | STR or biopsy | Glioblastoma, IDH–wild-type | OS | Jusue-Torres 2023 | 9 | serious risk | no serious inconsistency | no serious indirectness | no serious imprecision | undetected | would not reduce effect | yes | no | Low |
| GTR | STR | Glioblastoma, IDH–wild-type | PFS | Jusue-Torres 2023 | 2 | serious risk | no serious inconsistency | no serious indirectness | no serious imprecision | undetected | would not reduce effect | no | no | Very low |
| GTR | Biopsy | Glioblastoma, IDH–wild-type | PFS | Jusue-Torres 2023 | 3 | serious risk | no serious inconsistency | no serious indirectness | NA | undetected | would not reduce effect | yes | no | Very low |
| GTR | STR/biopsy | Glioblastoma, IDH–wild-type | PFS | Jusue-Torres 2023 | 5 | serious risk | no serious inconsistency | no serious indirectness | no serious imprecision | undetected | would not reduce effect | yes | no | Low |
| Intra-arterial CT | Intravenous CT | Malignant glioma | Leukocytopenia | Chen 2013 | 3 | no serious risk | no serious inconsistency | no serious indirectness | serious imprecision | strongly suspected | would not reduce effect | yes | no | Moderate |
| Vaccines | Conventional treatments | Malignant glioma | OS rate for 2 years | Jajin 2024 | 13 | no serious risk | serious inconsistency | no serious indirectness | no serious imprecision | undetected | would not reduce effect | yes | no | Low |
| Vaccines | Conventional treatments | Malignant glioma | PFS rate for 2 years | Jajin 2024 | 9 | no serious risk | serious inconsistency | no serious indirectness | no serious imprecision | undetected | would not reduce effect | yes | no | Low |
| Vaccines | Conventional treatments | Malignant glioma | Survival duration | Jajin 2024 | 13 | no serious risk | serious inconsistency | no serious indirectness | no serious imprecision | undetected | would not reduce effect | no | no | Very low |
| Vaccines | Conventional treatments | Malignant glioma | Skin reaction | Jajin 2024 | 7 | no serious risk | no serious inconsistency | no serious indirectness | serious imprecision | undetected | would not reduce effect | yes | no | Low |
| Vaccines | Conventional treatments | Malignant glioma | Flu-like syndrome | Jajin 2024 | 7 | no serious risk | no serious inconsistency | no serious indirectness | no serious imprecision | undetected | would not reduce effect | yes | no | Moderate |
| Vaccines | Conventional treatments | Primary malignant glioma | OS duration | Jajin 2024 | 6 | no serious risk | NA | no serious indirectness | serious imprecision | undetected | would not reduce effect | no | no | Very low |
| Vaccines | Conventional treatments | High-grade gliomas | OS duration | Jajin 2024 | 4 | no serious risk | NA | no serious indirectness | serious imprecision | undetected | would not reduce effect | no | no | Very low |
| Cytoreductive resection | Biopsy | Supratentorial high-grade glioma | OS | Tsitlakidis 2010 | 3 | serious risk | no serious inconsistency | no serious indirectness | serious imprecision | undetected | would not reduce effect | no | no | Very low |
| Cytoreductive resection | Biopsy | Grade IV/4 glioma | OS | Tsitlakidis 2010 | 3 | serious risk | serious inconsistency | no serious indirectness | serious imprecision | undetected | would not reduce effect | no | no | Very low |
| Cytoreductive resection | Biopsy | Grade III or IV glioma | OS | Tsitlakidis 2010 | 2 | serious risk | no serious inconsistency | no serious indirectness | serious imprecision | undetected | would not reduce effect | no | no | Very low |
| Cytoreductive resection | Biopsy | Elderly participants (≥ 65 years) with supratentorial high-grade glioma | OS | Tsitlakidis 2010 | 2 | serious risk | no serious inconsistency | no serious indirectness | no serious imprecision | undetected | would not reduce effect | no | no | Low |
| Cytoreductive resection | Biopsy | Supratentorial high-grade glioma with a wide age range | OS | Tsitlakidis 2010 | 3 | serious risk | serious inconsistency | no serious indirectness | serious imprecision | undetected | would not reduce effect | no | no | Very low |
| HFSRT + CT | Sole HFSRT management | Recurrent malignant glioma | OS | Hu 2019 | 5 | serious risk | no serious inconsistency | no serious indirectness | no serious imprecision | undetected | would not reduce effect | yes | no | Low |
| HFSRT + CT | Sole HFSRT management | Recurrent malignant glioma | OS | Hu 2019 | 6 | serious risk | no serious inconsistency | no serious indirectness | no serious imprecision | undetected | would not reduce effect | no | no | Very low |
| Awake craniotomy | Asleep craniotomy | Eloquent glioma | Extent of resection | Sattari 2024 | 11 | no serious risk | serious inconsistency | no serious indirectness | no serious imprecision | undetected | would not reduce effect | no | no | Very low |
| Awake craniotomy | Asleep craniotomy | Eloquent glioblastoma multiforme | Extent of resection | Sattari 2024 | 6 | no serious risk | serious inconsistency | no serious indirectness | serious imprecision | undetected | would not reduce effect | no | no | Very low |
| Awake craniotomy | Asleep craniotomy | Eloquent glioma | OS | Sattari 2024 | 4 | no serious risk | no serious inconsistency | no serious indirectness | no serious imprecision | undetected | would not reduce effect | no | no | Low |
| Awake craniotomy | Asleep craniotomy | Eloquent glioma | PFS | Sattari 2024 | 4 | no serious risk | serious inconsistency | no serious indirectness | serious imprecision | undetected | would not reduce effect | no | no | Very low |
| Awake craniotomy | Asleep craniotomy | Eloquent glioma | 3-month postoperative neurological deficits | Sattari 2024 | 10 | no serious risk | no serious inconsistency | no serious indirectness | serious imprecision | undetected | would not reduce effect | yes | no | Low |
| Awake craniotomy | Asleep craniotomy | Eloquent glioma | 3-month postoperative Karnofsky performance score | Sattari 2024 | 4 | no serious risk | no serious inconsistency | no serious indirectness | no serious imprecision | undetected | would not reduce effect | no | no | Low |
| Awake craniotomy | Asleep craniotomy | Eloquent glioma | 3-month postoperative seizure freedom | Sattari 2024 | 2 | no serious risk | no serious inconsistency | no serious indirectness | no serious imprecision | undetected | would not reduce effect | yes | no | Moderate |
| Awake craniotomy | Asleep craniotomy | Eloquent glioma | Length of hospital stay | Sattari 2024 | 3 | no serious risk | serious inconsistency | no serious indirectness | serious imprecision | undetected | would not reduce effect | no | no | Very low |
| Adjuvant RT | Received either salvage RT or no RT at all | Oligodendroglioma | OS | Ng 2024 | 15 | no serious risk | serious inconsistency | no serious indirectness | serious imprecision | undetected | would not reduce effect | no | no | Very low |
| Adjuvant RT | Received either salvage RT or no RT at all | Oligodendroglioma | PFS | Ng 2024 | 12 | no serious risk | no serious inconsistency | no serious indirectness | no serious imprecision | undetected | would not reduce effect | no | no | Low |
| Adjuvant RT | Received either salvage RT or no RT at all | Oligodendroglioma defined by IDH mutation and 1p/19q co-deletion | OS | Ng 2024 | 6 | no serious risk | serious inconsistency | no serious indirectness | serious imprecision | undetected | would not reduce effect | no | no | Very low |
| Adjuvant RT | Received either salvage RT or no RT at all | Oligodendroglioma defined by IDH mutation and 1p/19q co-deletion | PFS | Ng 2024 | 7 | no serious risk | no serious inconsistency | no serious indirectness | no serious imprecision | undetected | would not reduce effect | yes | no | Moderate |
| Adjuvant RT | Salvage RT | Oligodendroglioma | OS | Ng 2024 | 10 | no serious risk | serious inconsistency | no serious indirectness | serious imprecision | undetected | would not reduce effect | no | no | Very low |
| Adjuvant CRT | RT alone | Oligodendroglioma | OS | Ng 2024 | 5 | no serious risk | no serious inconsistency | no serious indirectness | no serious imprecision | undetected | would not reduce effect | no | no | Low |
| Adjuvant RT | Received either salvage RT or no RT at all | Grade 3 oligodendroglioma | OS | Ng 2024 | 6 | no serious risk | serious inconsistency | no serious indirectness | serious imprecision | undetected | would not reduce effect | no | no | Very low |
| Adjuvant RT | Salvage RT | Oligodendroglioma | PFS | Ng 2024 | 8 | no serious risk | no serious inconsistency | no serious indirectness | no serious imprecision | undetected | would not reduce effect | yes | no | Moderate |
| Adjuvant CRT | CT alone | Oligodendroglioma | PFS | Ng 2024 | 4 | no serious risk | serious inconsistency | no serious indirectness | no serious imprecision | undetected | would not reduce effect | yes | no | Low |
| Adjuvant CRT | Adjuvant RT | Oligodendroglioma | PFS | Ng 2024 | 5 | no serious risk | serious inconsistency | no serious indirectness | serious imprecision | undetected | would not reduce effect | no | no | Very low |
| Adjuvant RT | Received either salvage RT or no RT at all | Grade 2 oligodendroglioma | PFS | Ng 2024 | 4 | no serious risk | serious inconsistency | no serious indirectness | no serious imprecision | undetected | would not reduce effect | yes | no | Low |
| Adjuvant RT | Received either salvage RT or no RT at all | Grade 3 oligodendroglioma | PFS | Ng 2024 | 6 | no serious risk | serious inconsistency | no serious indirectness | no serious imprecision | undetected | would not reduce effect | no | no | Very low |
| Adjuvant RT | Adjuvant CT alone | Grade 3 to 5 oligodendroglioma | Adverse events | Ng 2024 | 2 | no serious risk | no serious inconsistency | no serious indirectness | no serious imprecision | undetected | would not reduce effect | no | no | Low |
| Adjuvant CRT | Adjuvant RT | Grade 3 to 5 oligodendroglioma | Adverse events | Ng 2024 | 2 | no serious risk | serious inconsistency | no serious indirectness | serious imprecision | undetected | would not reduce effect | yes | no | Very low |
| CRT | RT | IDH-wild-type gliomas | PFS | Kinslow 2024 | 5 | no serious risk | no serious inconsistency | no serious indirectness | serious imprecision | undetected | would not reduce effect | no | no | Moderate |
| CRT | RT | IDH-mutant gliomas | OS | Kinslow 2024 | 5 | no serious risk | no serious inconsistency | no serious indirectness | no serious imprecision | undetected | would not reduce effect | no | no | High |
| CRT | RT | IDH-mutant gliomas | PFS | Kinslow 2024 | 6 | no serious risk | no serious inconsistency | no serious indirectness | no serious imprecision | undetected | would not reduce effect | yes | no | High |
| Alkylating CT | RT | IDH-mutant gliomas | PFS | Kinslow 2024 | 4 | no serious risk | no serious inconsistency | no serious indirectness | serious imprecision | undetected | would not reduce effect | no | no | Moderate |
| CRT | RT | IDH-mutant and 1p19q-codeleted gliomas | OS | Kinslow 2024 | 4 | no serious risk | no serious inconsistency | no serious indirectness | serious imprecision | undetected | would not reduce effect | no | no | Moderate |
| CRT | RT | IDH-mutant and 1p19q-codeleted gliomas | PFS | Kinslow 2024 | 4 | no serious risk | no serious inconsistency | no serious indirectness | no serious imprecision | undetected | would not reduce effect | yes | no | High |
| CRT | RT | IDH-mutant and 1p19q-intact gliomas | OS | Kinslow 2024 | 4 | no serious risk | no serious inconsistency | no serious indirectness | no serious imprecision | undetected | would not reduce effect | no | no | High |
| CRT | RT | IDH-mutant and 1p19q-intact gliomas | PFS | Kinslow 2024 | 4 | no serious risk | no serious inconsistency | no serious indirectness | no serious imprecision | undetected | would not reduce effect | yes | no | High |
| Anti-PD-1/PD-L1 treatment | RT and TMZ with or without placebo with/ BV | Glioma | OS | Zeng 2023 | 3 | no serious risk | no serious inconsistency | no serious indirectness | serious imprecision | undetected | would not reduce effect | no | no | Moderate |
| Anti-PD-1/PD-L1 treatment | Placebo with RT and TMZ | Glioma | PFS | Zeng 2023 | 3 | no serious risk | serious inconsistency | no serious indirectness | serious imprecision | undetected | would not reduce effect | no | no | Low |
| *Not Significant associations* | | | | | | | | | | | | | | |
| HFRT | CFRT | DIPG | OS | Park 2020 | 4 | no serious risk | no serious inconsistency | no serious indirectness | serious imprecision | undetected | would not reduce effect | no | no | Very low |
| HFRT | CFRT | DIPG | PFS | Park 2020 | 4 | no serious risk | no serious inconsistency | no serious indirectness | serious imprecision | undetected | would not reduce effect | no | no | Very low |
| BV plus RT/TMZ | RT/TMZ | High-grade gliomas | 6-month survival rate | Fu 2016 | 3 | no serious risk | serious inconsistency | no serious indirectness | serious imprecision | NA | would not reduce effect | no | no | Low |
| Viral therapy | Standard therapy | High-grade gliomas | PFS | Vatu 2019 | 4 | no serious risk | serious inconsistency | no serious indirectness | serious imprecision | undetected | would not reduce effect | no | no | Very low |
| DC vaccination | Control therapy | High-grade gliomas | 0.5-years OS | Li 2018 | 11 | no serious risk | no serious inconsistency | no serious indirectness | serious imprecision | undetected | would not reduce effect | no | no | Very low |
| DC vaccination | Control therapy | High-grade gliomas | 0.5-years PFS | Li 2018 | 5 | no serious risk | no serious inconsistency | no serious indirectness | serious imprecision | undetected | would not reduce effect | no | no | Very low |
| DC vaccination | Control therapy | High-grade gliomas | 1-years PFS | Li 2018 | 4 | no serious risk | serious inconsistency | no serious indirectness | serious imprecision | undetected | would not reduce effect | no | no | Very low |
| CRT | RT alone | High-grade gliomas | Survival | Stewart 2002 | 12 | no serious risk | no serious inconsistency | no serious indirectness | no serious imprecision | NA | would not reduce effect | no | no | High |
| Gliadel | Placebo | High-grade gliomas | Survival | Hart 2008 | 2 | no serious risk | serious inconsistency | no serious indirectness | serious imprecision | NA | would not reduce effect | no | no | Low |
| Gene therapy | Standard treatment | High-grade gliomas | Tumor progression | Zhao 2014 | 4 | no serious risk | no serious inconsistency | no serious indirectness | serious imprecision | NA | would not reduce effect | no | no | Moderate |
| Gene therapy | Standard treatment | High-grade gliomas | OS | Zhao 2014 | 3 | no serious risk | no serious inconsistency | no serious indirectness | serious imprecision | NA | would not reduce effect | no | no | Moderate |
| Gene therapy | Standard treatment | Glioblastoma | OS | Zhao 2014 | 2 | no serious risk | no serious inconsistency | no serious indirectness | serious imprecision | NA | would not reduce effect | no | no | Moderate |
| Carmustine Wafers | Stupp Regimen | High-grade gliomas | PFS | Ricciardi 2022 | 4 | serious risk | serious inconsistency | no serious indirectness | serious imprecision | NA | would not reduce effect | no | no | Very low |
| Combination of viral therapy and SOC | SOC alone | High-grade gliomas | OS | Guo 2023 | 6 | serious risk | serious inconsistency | no serious indirectness | serious imprecision | strongly suspected | would not reduce effect | no | no | Very low |
| Combination of viral therapy and SOC | SOC alone | High-grade gliomas | PFS | Guo 2023 | 2 | serious risk | serious inconsistency | no serious indirectness | serious imprecision | strongly suspected | would not reduce effect | no | no | Very low |
| Combination of multiple courses of treatment/multi-point injection/ small injection volume viral therapy and SOC compared | SOC alone | High-grade gliomas | PFS | Guo 2023 | 2 | serious risk | serious inconsistency | no serious indirectness | serious imprecision | undetected | would not reduce effect | no | no | Very low |
| Combination of DC therapy and SOC | SOC alone | High-grade gliomas | PFS | Guo 2023 | 3 | serious risk | no serious inconsistency | no serious indirectness | no serious imprecision | undetected | would not reduce effect | no | no | Low |
| Combination of immunopotentiators and SOC | SOC alone | High-grade gliomas | OS | Guo 2023 | 2 | serious risk | no serious inconsistency | no serious indirectness | serious imprecision | strongly suspected | would not reduce effect | no | no | Very low |
| TMZ | HFRT | Elderly patients (≥60 y) with high-grade gliomas | Survival | Hart 2013 | 1 | no serious risk | NA | no serious indirectness | serious imprecision | undetected | would not reduce effect | no | no | Low |
| TMZ | Standard RT | Elderly patients (≥60 y) with high-grade gliomas | Survival | Hart 2013 | 2 | no serious risk | serious inconsistency | no serious indirectness | serious imprecision | undetected | would not reduce effect | no | no | Low |
| TMZ | Standard RT | Elderly patients (≥60 y) with high-grade gliomas | PFS | Hart 2013 | 1 | no serious risk | NA | no serious indirectness | serious imprecision | undetected | would not reduce effect | no | no | Low |
| Dose‐dense TMZ | Metronomic TMZ high-grade gliomas | High-grade gliomas | Survival | Hart 2013 | 1 | no serious risk | NA | no serious indirectness | serious imprecision | undetected | would not reduce effect | no | no | Low |
| TMZ | Nitrosourea CT | Recurrent glioblastoma | Survival | Hart 2013 | 2 | no serious risk | no serious inconsistency | no serious indirectness | serious imprecision | undetected | would not reduce effect | no | no | Moderate |
| TMZ | Nitrosourea CT | Recurrent glioblastoma | PFS | Hart 2013 | 2 | no serious risk | serious inconsistency | no serious indirectness | serious imprecision | undetected | would not reduce effect | no | no | Low |
| TMZ | Nitrosourea CT | Recurrent glioblastoma | Adverse events | Hart 2013 | 3 | no serious risk | no serious inconsistency | no serious indirectness | serious imprecision | undetected | would not reduce effect | no | no | Moderate |
| Reirradiation | Systemic therapy | Recurrent high-grade glioma | OS | Marwah 2023 | 3 | no serious risk | no serious inconsistency | no serious indirectness | serious imprecision | undetected | would not reduce effect | no | no | Very low |
| Reirradiation | Systemic therapy | Recurrent high-grade glioma | PFS | Marwah 2023 | 2 | no serious risk | no serious inconsistency | no serious indirectness | serious imprecision | undetected | would not reduce effect | no | no | Very low |
| Combination therapy | Systemic therapy | Recurrent high-grade glioma | Toxicity-CTCAE Grade 3+ | Marwah 2023 | 5 | no serious risk | no serious inconsistency | no serious indirectness | serious imprecision | undetected | would not reduce effect | no | no | Very low |
| Combination therapy | Systemic therapy | Recurrent high-grade glioma(RCT Only) | OS | Marwah 2023 | 2 | no serious risk | no serious inconsistency | no serious indirectness | serious imprecision | undetected | would not reduce effect | no | no | Very low |
| Combination therapy | Systemic therapy | Recurrent high-grade glioma(RCT Only) | PFS | Marwah 2023 | 2 | no serious risk | serious inconsistency | no serious indirectness | serious imprecision | undetected | would not reduce effect | no | no | Very low |
| Combination therapy | Systemic therapy | Recurrent high-grade glioma(RCT Only) | Toxicity-CTCAE Grade 3+ | Marwah 2023 | 2 | no serious risk | no serious inconsistency | no serious indirectness | serious imprecision | undetected | would not reduce effect | no | no | Very low |
| STR | Biopsy | Low-Grade Glioma | Mortality at 5 years | Brown 2019 | 8 | serious risk | serious inconsistency | no serious indirectness | serious imprecision | NA | would not reduce effect | no | no | Very low |
| STR | Biopsy | Low-Grade Glioma | Mortality at 10 years | Brown 2019 | 3 | serious risk | no serious inconsistency | no serious indirectness | serious imprecision | NA | would not reduce effect | no | no | Very low |
| Early Radiation | Late/No Radiation | Low-Grade Glioma | Mortality at 2 years | Brown 2019 | 9 | serious risk | serious inconsistency | no serious indirectness | serious imprecision | NA | would not reduce effect | no | no | Very low |
| Early Radiation | Late/No Radiation | Low-Grade Glioma | Mortality at 5 years | Brown 2019 | 10 | serious risk | serious inconsistency | no serious indirectness | serious imprecision | NA | would not reduce effect | no | no | Very low |
| Early Radiation | Late/No Radiation | Low-Grade Glioma | Mortality at 10 years | Brown 2019 | 6 | serious risk | serious inconsistency | no serious indirectness | serious imprecision | NA | would not reduce effect | no | no | Very low |
| CT | RT | Low-Grade Glioma | Mortality at 2 years | Brown 2019 | 5 | serious risk | no serious inconsistency | no serious indirectness | serious imprecision | NA | would not reduce effect | no | no | Very low |
| CT | RT | Low-Grade Glioma | Mortality at 5 years | Brown 2019 | 5 | serious risk | no serious inconsistency | no serious indirectness | serious imprecision | NA | would not reduce effect | no | no | Very low |
| CT | RT | Low-Grade Glioma | Mortality at 10 years | Brown 2019 | 3 | serious risk | no serious inconsistency | no serious indirectness | serious imprecision | NA | would not reduce effect | no | no | Very low |
| CT | RT | Low-Grade Glioma | Progression at 2 years | Brown 2019 | 3 | serious risk | no serious inconsistency | no serious indirectness | serious imprecision | NA | would not reduce effect | no | no | Very low |
| STR | Biopsy | pHGGs | 1-Year Mortality | Hatoum 2022 | 23 | no serious risk | no serious inconsistency | no serious indirectness | serious imprecision | undetected | would not reduce effect | no | no | Very low |
| STR | Biopsy | pHGGs | 6-Month Progression | Hatoum 2022 | 15 | no serious risk | no serious inconsistency | no serious indirectness | serious imprecision | undetected | would not reduce effect | no | no | Very low |
| STR | Biopsy | pHGGs | 1-Year Progression | Hatoum 2022 | 15 | no serious risk | no serious inconsistency | no serious indirectness | serious imprecision | undetected | would not reduce effect | no | no | Very low |
| SMR | GTR | Glioblastoma, IDH wild-type | OS | Mier-García 2023 | 4 | serious risk | serious inconsistency | no serious indirectness | serious imprecision | NA | would not reduce effect | no | no | Very low |
| GTR | STR | Glioma | 2-year PFS | Shi 2023 | 2 | no serious risk | no serious inconsistency | no serious indirectness | serious imprecision | undetected | would not reduce effect | no | no | Very low |
| GTR | STR | Glioma | 10-year PFS | Shi 2023 | 5 | no serious risk | serious inconsistency | no serious indirectness | serious imprecision | undetected | would not reduce effect | no | no | Very low |
| GTR | STR | Glioma | PFS | Shi 2023 | 2 | no serious risk | serious inconsistency | no serious indirectness | serious imprecision | undetected | would not reduce effect | no | no | Very low |
| GTR | STR | Glioma | Tumor progression | Shi 2023 | 3 | no serious risk | serious inconsistency | no serious indirectness | serious imprecision | undetected | would not reduce effect | no | no | Very low |
| Conventional surgery | iMRI | Glioma | Mean extent of resection | Lo 2021 | 2 | no serious risk | serious inconsistency | no serious indirectness | NA | undetected | would not reduce effect | no | no | Low |
| Conventional surgery | iMRI | Glioma | PFS | Lo 2021 | 2 | no serious risk | no serious inconsistency | no serious indirectness | serious imprecision | undetected | would not reduce effect | no | no | Moderate |
| Conventional surgery | iMRI | Glioma | OS | Lo 2021 | 3 | no serious risk | no serious inconsistency | no serious indirectness | serious imprecision | undetected | would not reduce effect | no | no | Moderate |
| Conventional surgery | iMRI | Glioma | LOS | Lo 2021 | 4 | no serious risk | serious inconsistency | no serious indirectness | serious imprecision | undetected | would not reduce effect | no | no | Low |
| Targeted combined CRT | CRT alone | Glioma | OS | Ma 2023 | 12 | no serious risk | serious inconsistency | no serious indirectness | serious imprecision | undetected | would not reduce effect | no | no | Low |
| Targeted combined CRT | CRT alone | Glioma | PFS | Ma 2023 | 11 | no serious risk | serious inconsistency | no serious indirectness | serious imprecision | undetected | would not reduce effect | no | no | Low |
| Targeted combined CRT | CRT alone | Glioma | Adverse events | Ma 2023 | 2 | no serious risk | serious inconsistency | no serious indirectness | serious imprecision | undetected | would not reduce effect | no | no | Low |
| Oral contraceptives | RT and TMZ | Glioma | Cumulative risk | Lan 2018 | 9 | no serious risk | no serious inconsistency | no serious indirectness | NA | undetected | would not reduce effect | no | no | Very low |
| Hormone replacement therapy | RT and TMZ | Glioma | Cumulative risk | Lan 2018 | 4 | no serious risk | no serious inconsistency | no serious indirectness | NA | undetected | would not reduce effect | no | no | Very low |
| Immunotherapy | Standard care treatments(included surgical resection, RT, or CT) | Glioma | Mean survival time | Hanaei 2018 | 7 | serious risk | serious inconsistency | no serious indirectness | serious imprecision | NA | would not reduce effect | no | no | Very low |
| Immunotherapy | Standard care treatments(included surgical resection, RT, or CT) | Glioma | 1-year PFS | Hanaei 2018 | 3 | serious risk | no serious inconsistency | no serious indirectness | serious imprecision | NA | would not reduce effect | no | no | Very low |
| Awake craniotomy | General anesthetic resection | Glioma | Early language deficit | Bu 2021 | 8 | no serious risk | serious inconsistency | no serious indirectness | serious imprecision | undetected | would not reduce effect | no | no | Very low |
| Awake craniotomy | General anesthetic resection | Glioma | Late language deficit | Bu 2021 | 7 | no serious risk | no serious inconsistency | no serious indirectness | serious imprecision | undetected | would not reduce effect | no | no | Very low |
| Awake craniotomy | General anesthetic resection | Glioma | Early motor deficit | Bu 2021 | 8 | no serious risk | serious inconsistency | no serious indirectness | serious imprecision | undetected | would not reduce effect | no | no | Very low |
| Awake craniotomy | General anesthetic resection | Glioma | Late motor deficit | Bu 2021 | 7 | no serious risk | no serious inconsistency | no serious indirectness | serious imprecision | undetected | would not reduce effect | no | no | Very low |
| Awake craniotomy | General anesthetic resection | Glioma | Early neurological deficit | Bu 2021 | 9 | no serious risk | serious inconsistency | no serious indirectness | serious imprecision | undetected | would not reduce effect | no | no | Very low |
| Awake craniotomy | General anesthetic resection | Glioma | Extend of tumor resection | Bu 2021 | 10 | no serious risk | no serious inconsistency | no serious indirectness | serious imprecision | undetected | would not reduce effect | no | no | Very low |
| Awake craniotomy | General anesthetic resection | Glioma | Mean operation time | Bu 2021 | 4 | no serious risk | serious inconsistency | no serious indirectness | serious imprecision | undetected | would not reduce effect | no | no | Very low |
| Awake craniotomy | General anesthetic resection | Glioma | Mean hospital stay | Bu 2021 | 4 | no serious risk | serious inconsistency | no serious indirectness | serious imprecision | undetected | would not reduce effect | no | no | Very low |
| Integrated Traditional Chinese and Western medicine | Western medical treatment | Glioma | One-year survival time | Yu 2021 | 5 | no serious risk | no serious inconsistency | no serious indirectness | serious imprecision | undetected | would not reduce effect | no | no | Moderate |
| Targeted therapy(including Regorafenib or Galunisertib or Depatux-M or Enzastaurin or Cediranib) | Lomustine | Glioblastoma | OS | Ippen 2024 | 5 | no serious risk | serious inconsistency | no serious indirectness | serious imprecision | undetected | would not reduce effect | no | no | Low |
| Targeted therapy(including Regorafenib or Galunisertib or Depatux-M or Enzastaurin or Cediranib) plus Lomustine | Lomustine | Glioblastoma | OS | Ippen 2024 | 5 | no serious risk | no serious inconsistency | no serious indirectness | serious imprecision | undetected | would not reduce effect | no | no | Moderate |
| Targeted therapy(including Regorafenib or Galunisertib or Depatux-M or Enzastaurin or Cediranib) | BV | Glioblastoma | OS | Ippen 2024 | 8 | no serious risk | no serious inconsistency | no serious indirectness | serious imprecision | undetected | would not reduce effect | no | no | Moderate |
| Targeted therapy(including Regorafenib or Galunisertib or Depatux-M or Enzastaurin or Cediranib) | Lomustine | Glioblastoma | PFS | Ippen 2024 | 4 | no serious risk | serious inconsistency | no serious indirectness | serious imprecision | undetected | would not reduce effect | no | no | Low |
| Targeted therapy(including Regorafenib or Galunisertib or Depatux-M or Enzastaurin or Cediranib) | BV | Glioblastoma | PFS | Ippen 2024 | 9 | no serious risk | serious inconsistency | no serious indirectness | serious imprecision | undetected | would not reduce effect | no | no | Low |
| Targeted therapy | TMZ | Glioblastoma | OS | Scherm 2023 | 11 | no serious risk | serious inconsistency | no serious indirectness | serious imprecision | undetected | would not reduce effect | no | no | Low |
| Molecularly targeted drugs | TMZ plus RT | Glioblastoma | OS | Wang 2019 | 15 | no serious risk | no serious inconsistency | no serious indirectness | serious imprecision | undetected | would not reduce effect | no | no | Moderate |
| Antiangiogenic drugs | Cytotoxic drug alone | Glioblastoma | OS | Lombardi 2017 | 17 | no serious risk | no serious inconsistency | no serious indirectness | serious imprecision | undetected | would not reduce effect | no | no | Moderate |
| Higher dose of carmustine | Lower dose of carmustine | Glioblastoma | OS | Xiao 2020 | 3 | no serious risk | no serious inconsistency | no serious indirectness | serious imprecision | undetected | would not reduce effect | no | no | Very low |
| Standard therapy + Anti-vascular endothelial growth factor | Standard therapy | Glioblastoma | OS | Xiao 2018 | 6 | no serious risk | serious inconsistency | no serious indirectness | serious imprecision | NA | would not reduce effect | no | no | Low |
| Standard therapy + BV | Standard therapy | Glioblastoma | OS | Xiao 2018 | 4 | no serious risk | serious inconsistency | no serious indirectness | serious imprecision | NA | would not reduce effect | no | no | Low |
| Molecularly targeted drugs combined with TMZ plus RT | TMZ plus RT | Glioblastoma | OS | Su 2016 | 5 | no serious risk | no serious inconsistency | no serious indirectness | serious imprecision | undetected | would not reduce effect | no | no | Moderate |
| Molecularly targeted drugs combined with TMZ plus RT | TMZ plus RT | MGMT-methylated patients | PFS | Su 2016 | 3 | no serious risk | serious inconsistency | no serious indirectness | serious imprecision | undetected | would not reduce effect | no | no | Low |
| Molecularly targeted drugs combined with TMZ plus RT | TMZ plus RT | MGMT-methylated patients | OS | Su 2016 | 3 | no serious risk | no serious inconsistency | no serious indirectness | serious imprecision | undetected | would not reduce effect | no | no | Moderate |
| Molecularly targeted drugs combined with TMZ plus RT | TMZ plus RT | MGMT-nonmethylated patients | OS | Su 2016 | 3 | no serious risk | no serious inconsistency | no serious indirectness | serious imprecision | undetected | would not reduce effect | no | no | Moderate |
| BV combined with TMZ plus RT | TMZ plus RT | Glioblastoma | PFS | Su 2016 | 3 | no serious risk | no serious inconsistency | no serious indirectness | serious imprecision | undetected | would not reduce effect | no | no | Moderate |
| Cilengitide | TMZ plus RT | Glioblastoma | OS | Su 2016 | 2 | no serious risk | no serious inconsistency | no serious indirectness | serious imprecision | undetected | would not reduce effect | no | no | Moderate |
| Levetiracetam plus SOC | SOC | Glioblastoma | OS | Chen 2022 | 11 | no serious risk | serious inconsistency | no serious indirectness | serious imprecision | strongly suspected | would not reduce effect | no | no | Very low |
| Levetiracetam plus SOC | Other AED treatment | Glioblastoma | Adverse events | Chen 2022 | 8 | no serious risk | serious inconsistency | no serious indirectness | serious imprecision | strongly suspected | would not reduce effect | no | no | Very low |
| BV plus other CRT | Other CRT | Newly diagnosed glioblastoma | All-Cause Discontinuation | Li 2015 | 3 | no serious risk | serious inconsistency | no serious indirectness | serious imprecision | undetected | would not reduce effect | no | no | Low |
| BV plus other CRT | Other CRT | Newly diagnosed glioblastoma | Thrombocytopenia | Li 2015 | 2 | no serious risk | serious inconsistency | no serious indirectness | serious imprecision | undetected | would not reduce effect | no | no | Low |
| BV plus other CRT | Other CRT | Newly diagnosed glioblastoma | Deep Vein Thrombosis | Li 2015 | 2 | no serious risk | serious inconsistency | no serious indirectness | serious imprecision | undetected | would not reduce effect | no | no | Low |
| BV plus other CRT | Other CRT | Newly diagnosed glioblastoma | Pulmonary Embolism | Li 2015 | 2 | no serious risk | no serious inconsistency | no serious indirectness | serious imprecision | undetected | would not reduce effect | no | no | Moderate |
| BV in combination with CRT(including TMZ or Lomustine) | CRT alone(including TMZ or Lomustine) | Glioblastoma | OS | Lan 2022 | 7 | no serious risk | no serious inconsistency | no serious indirectness | serious imprecision | undetected | would not reduce effect | no | no | Moderate |
| Combination therapy of BV plus CT | BV or CT alone | Glioblastoma | OS | Yang 2017 | 4 | no serious risk | no serious inconsistency | no serious indirectness | serious imprecision | strongly suspected | would not reduce effect | no | no | Low |
| High cumulative dose TMZ | Normal cumulative dose TMZ | Glioblastoma | OS | Sun 2015 | 3 | no serious risk | no serious inconsistency | no serious indirectness | serious imprecision | undetected | would not reduce effect | no | no | Moderate |
| Higher peak concentration TMZ | lower peak TMZ | Glioblastoma | OS | Sun 2015 | 3 | no serious risk | no serious inconsistency | no serious indirectness | serious imprecision | undetected | would not reduce effect | no | no | Moderate |
| High cumulative dose TMZ | Normal cumulative dose TMZ | Glioblastoma | PFS | Sun 2015 | 2 | no serious risk | serious inconsistency | no serious indirectness | serious imprecision | undetected | would not reduce effect | no | no | Low |
| HFRT | Conventional fraction RT | Glioblastoma | OS | Liao 2019 | 4 | no serious risk | no serious inconsistency | no serious indirectness | serious imprecision | undetected | would not reduce effect | no | no | Moderate |
| HFRT | Conventional fraction RT | Glioblastoma | PFS | Liao 2019 | 2 | no serious risk | serious inconsistency | no serious indirectness | serious imprecision | undetected | would not reduce effect | no | no | Low |
| CRT | RT | Glioblastoma | PFS | Wang 2017 | 4 | no serious risk | serious inconsistency | no serious indirectness | serious imprecision | strongly suspected | would not reduce effect | no | no | Very low |
| RT plus TMZ | RT alone | Glioblastoma | Hematological complications | Zhao 2021 | 3 | no serious risk | serious inconsistency | no serious indirectness | serious imprecision | NA | would not reduce effect | no | no | Moderate |
| RT plus TMZ | RT alone | Glioblastoma | Serious adverse events | Zhao 2021 | 2 | no serious risk | no serious inconsistency | no serious indirectness | serious imprecision | NA | would not reduce effect | no | no | Low |
| Anti-EGFR therapies | Placebo | Glioblastoma | OS | Lee 2020 | 3 | no serious risk | no serious inconsistency | no serious indirectness | serious imprecision | undetected | would not reduce effect | no | no | Moderate |
| Anti-EGFR therapies with CT | SOC with CT | Recurrent Glioblastoma | OS | Lee 2020 | 4 | no serious risk | serious inconsistency | no serious indirectness | serious imprecision | undetected | would not reduce effect | no | no | Low |
| Anti-EGFR therapies | Placebo | Glioblastoma | PFS | Lee 2020 | 2 | no serious risk | no serious inconsistency | no serious indirectness | serious imprecision | undetected | would not reduce effect | no | no | Moderate |
| Anti-EGFR therapies | SOC | Glioblastoma | Lymphopenia | Lee 2020 | 4 | no serious risk | serious inconsistency | no serious indirectness | serious imprecision | undetected | would not reduce effect | no | no | Low |
| Anti-EGFR therapies | SOC | Glioblastoma | Neutropenia | Lee 2020 | 4 | no serious risk | no serious inconsistency | no serious indirectness | serious imprecision | undetected | would not reduce effect | no | no | Moderate |
| Anti-EGFR therapies | SOC | Glioblastoma | Thrombocytopenia | Lee 2020 | 4 | no serious risk | serious inconsistency | no serious indirectness | serious imprecision | undetected | would not reduce effect | no | no | Low |
| Anti-EGFR therapies | SOC | Glioblastoma | Rash | Lee 2020 | 4 | no serious risk | serious inconsistency | no serious indirectness | serious imprecision | undetected | would not reduce effect | no | no | Low |
| Anti-EGFR therapies | SOC | Glioblastoma | Diarrhoea | Lee 2020 | 5 | no serious risk | no serious inconsistency | no serious indirectness | serious imprecision | undetected | would not reduce effect | no | no | Moderate |
| Anti-EGFR therapies | SOC | Glioblastoma | Fatigue | Lee 2020 | 5 | no serious risk | serious inconsistency | no serious indirectness | serious imprecision | undetected | would not reduce effect | no | no | Low |
| STR | Biopsy | Glioblastoma | 2-Year Mortality | Brown 2016 | 16 | no serious risk | no serious inconsistency | no serious indirectness | serious imprecision | undetected | would not reduce effect | no | no | Very low |
| Resection | Biopsy | Glioblastoma | 2-Year Mortality | Brown 2016 | 17 | no serious risk | serious inconsistency | no serious indirectness | serious imprecision | undetected | would not reduce effect | no | no | Very low |
| GTR | STR | Glioblastoma | Progression at six months | Brown 2016 | 6 | no serious risk | serious inconsistency | no serious indirectness | serious imprecision | undetected | would not reduce effect | no | no | Very low |
| Resection | Biopsy | Glioblastoma | Progression at six months | Brown 2016 | 4 | no serious risk | serious inconsistency | no serious indirectness | serious imprecision | undetected | would not reduce effect | no | no | Very low |
| Lobectomy | GTR | Glioblastoma | KPS scores | Zheng 2023 | 3 | no serious risk | serious inconsistency | no serious indirectness | serious imprecision | NA | would not reduce effect | no | no | Very low |
| Active immunotherapy | Standard therapy（combination of surgical resection, RT or CT） | Glioblastoma | OS | Wahyuhadi 2022 | 6 | serious risk | serious inconsistency | no serious indirectness | serious imprecision | NA | would not reduce effect | no | no | Very low |
| Active immunotherapy | Standard therapy（combination of surgical resection, RT or CT） | Glioblastoma | PFS | Wahyuhadi 2022 | 6 | serious risk | no serious inconsistency | no serious indirectness | serious imprecision | NA | would not reduce effect | no | no | Low |
| BV plus other CRT | Other CRT | Recurrent glioblastoma | Median OS | LI 2016 | 5 | no serious risk | serious inconsistency | no serious indirectness | serious imprecision | undetected | would not reduce effect | no | no | Moderate |
| BV plus some specific cytotoxic treatments | A certain cytotoxic treatment | Recurrent glioblastoma | Median OS | Zhang 2021 | 5 | no serious risk | no serious inconsistency | no serious indirectness | serious imprecision | NA | would not reduce effect | no | no | Moderate |
| BV plus some specific cytotoxic treatments | A certain cytotoxic treatment | Recurrent glioblastoma | 6-month PFS | Zhang 2021 | 3 | no serious risk | no serious inconsistency | no serious indirectness | serious imprecision | NA | would not reduce effect | no | no | Moderate |
| BV plus some specific cytotoxic treatments | A certain cytotoxic treatment | Recurrent glioblastoma | 12-month OS | Zhang 2021 | 5 | no serious risk | no serious inconsistency | no serious indirectness | serious imprecision | NA | would not reduce effect | no | no | Moderate |
| TMZ alone | RT alone | Elderly glioblastoma | OS | Yin 2014 | 5 | no serious risk | no serious inconsistency | no serious indirectness | serious imprecision | undetected | would not reduce effect | no | no | Very low |
| TMZ alone | RT alone | Elderly glioblastoma | PFS | Yin 2014 | 2 | no serious risk | serious inconsistency | no serious indirectness | serious imprecision | undetected | would not reduce effect | no | no | Very low |
| GTR | Biopsy | Glioblastoma, IDH–wild-type | PFS | Jusue-Torres 2023 | 2 | serious risk | serious inconsistency | no serious indirectness | serious imprecision | undetected | would not reduce effect | no | no | Very low |
| STR | Biopsy | Glioblastoma, IDH–wild-type | PFS | Jusue-Torres 2023 | 2 | serious risk | serious inconsistency | no serious indirectness | serious imprecision | undetected | would not reduce effect | no | no | Very low |
| Intra-arterial CT | Intravenous CT | Malignant glioma | Disease control rate | Chen 2013 | 2 | no serious risk | serious inconsistency | no serious indirectness | serious imprecision | strongly suspected | would not reduce effect | no | no | Very low |
| Intra-arterial CT | Intravenous CT | Malignant glioma | Effective rate | Chen 2013 | 2 | no serious risk | serious inconsistency | no serious indirectness | serious imprecision | strongly suspected | would not reduce effect | no | no | Very low |
| Intra-arterial CT | Intravenous CT | Malignant glioma | Thrombocytopenia | Chen 2013 | 3 | no serious risk | no serious inconsistency | no serious indirectness | serious imprecision | strongly suspected | would not reduce effect | no | no | Low |
| Intra-arterial CT | Intravenous CT | Malignant glioma | Anemia | Chen 2013 | 4 | no serious risk | serious inconsistency | no serious indirectness | serious imprecision | strongly suspected | would not reduce effect | no | no | Very low |
| Hyperfractionated RT | Conventional fractionation RT | Malignant glioma | 1-Year Mortality | Laperriere 2002 | 4 | serious risk | NA | no serious indirectness | serious imprecision | undetected | would not reduce effect | no | no | Very low |
| Vaccines | Conventional treatments | Malignant glioma | Lymphopenia | Jajin 2024 | 5 | no serious risk | serious inconsistency | no serious indirectness | serious imprecision | undetected | would not reduce effect | no | no | Very low |
| Vaccines | Conventional treatments | Recurrent malignant glioma | OS duration | Jajin 2024 | 6 | no serious risk | NA | no serious indirectness | no serious imprecision | undetected | would not reduce effect | no | no | Very low |
| Vaccines | Conventional treatments | Low-Grade Glioma | OS duration | Jajin 2024 | 9 | no serious risk | NA | no serious indirectness | serious imprecision | undetected | would not reduce effect | no | no | Very low |
| Awake craniotomy | Asleep craniotomy | Eloquent glioma | Duration of operation | Sattari 2024 | 3 | no serious risk | serious inconsistency | no serious indirectness | serious imprecision | undetected | would not reduce effect | no | no | Very low |
| Adjuvant RT | Adjuvant CT alone | Oligodendroglioma | OS | Ng 2024 | 4 | no serious risk | serious inconsistency | no serious indirectness | serious imprecision | undetected | would not reduce effect | no | no | Very low |
| Adjuvant CRT | CT alone | Oligodendroglioma | OS | Ng 2024 | 4 | no serious risk | serious inconsistency | no serious indirectness | serious imprecision | undetected | would not reduce effect | no | no | Very low |
| Adjuvant RT | Received either salvage RT or no RT at all | Grade 2 oligodendroglioma | OS | Ng 2024 | 6 | no serious risk | serious inconsistency | no serious indirectness | serious imprecision | undetected | would not reduce effect | no | no | Very low |
| Adjuvant RT | Adjuvant CT alone | Oligodendroglioma | PFS | Ng 2024 | 4 | no serious risk | serious inconsistency | no serious indirectness | serious imprecision | undetected | would not reduce effect | no | no | Very low |
| CRT | RT | IDH-wild-type gliomas | OS | Kinslow 2024 | 5 | no serious risk | no serious inconsistency | no serious indirectness | serious imprecision | strongly suspected | would not reduce effect | no | no | Low |
| Alkylating CT | RT | IDH-wild-type gliomas | PFS | Kinslow 2024 | 2 | no serious risk | serious inconsistency | no serious indirectness | serious imprecision | undetected | would not reduce effect | no | no | Low |
| Neutron beam therapy | Photon therapy | High-grade gliomas | 12-month overall mortality | Maucort-Boulch 2010 | 4 | serious risk | no serious inconsistency | no serious indirectness | serious imprecision | undetected | would not reduce effect | no | no | Low |
| Neutron beam therapy | Photon therapy | High-grade gliomas | 24-month overall mortality | Maucort-Boulch 2010 | 4 | serious risk | no serious inconsistency | no serious indirectness | serious imprecision | undetected | would not reduce effect | no | no | Low |
| Reduced-dose BV | Standard -dose BV | Recurrent high-grade glioma or glioblastoma | OS | Chen 2020 | 5 | serious risk | serious inconsistency | no serious indirectness | serious imprecision | undetected | would not reduce effect | no | no | Very low |
| Reduced-dose BV | Standard -dose BV | Recurrent high-grade glioma or glioblastoma | PFS | Chen 2020 | 4 | serious risk | serious inconsistency | no serious indirectness | serious imprecision | undetected | would not reduce effect | no | no | Very low |
| Resection | Biopsy | Butterfly glioblastoma | Rates of postoperative defcit development | Soliman 2022 | 13 | no serious risk | no serious inconsistency | no serious indirectness | serious imprecision | NA | would not reduce effect | no | no | Very low |
| Resection | Biopsy | Elderly patients (≥60 y) with high-grade gliomas | Morbidity | Almenawer 2015 | 9 | no serious risk | no serious inconsistency | no serious indirectness | serious imprecision | strongly suspected | would not reduce effect | no | no | Very low |
| BV plus RT/TMZ | RT/TMZ | High-grade gliomas | OS | Fu 2016 | 3 | no serious risk | serious inconsistency | no serious indirectness | serious imprecision | NA | would not reduce effect | no | no | Low |
| CT | RT | IDH-mutant and 1p19q-codeleted gliomas | OS | Kinslow 2024 | 2 | no serious risk | no serious inconsistency | no serious indirectness | serious imprecision | undetected | would not reduce effect | no | no | Moderate |
| CT | RT | IDH-mutant and 1p19q-codeleted gliomas | PFS | Kinslow 2024 | 3 | no serious risk | no serious inconsistency | no serious indirectness | serious imprecision | undetected | would not reduce effect | no | no | Moderate |
| RT plus alkylating CT | RT | IDH-wild-type gliomas | OS | Kinslow 2024 | 4 | no serious risk | no serious inconsistency | no serious indirectness | serious imprecision | undetected | would not reduce effect | no | no | Moderate |
| GTR, gross total resection; STR, subtotal resection; RT, radiotherapy; CT, chemotherapy; CRT, chemoradiotherapy; HFSRT, hypofractionated stereotactic radiotherapy; SOC, standard of care; BV, bevacizumab; TMZ, temozolomide; AED, anti-epileptic drug; EGFR, epidermal growth factor receptor; HFRT, hypofractionated radiotherapy; CFRT, conventional fractionated radiotherapy; DIPG, diffuse intrinsic pontine gliomas; pHGGs, infratentorial pediatric high-grade gliomas; iMRI, intraoperative magnetic resonance imaging.. | | | | | | | | | | | | | | |
