# Supplementary material for: Comparative efficacy of glioma treatment strategies: an umbrella review of meta-analyses
Source: Ann Med. 2025 Jul 1;57(1):2525394. doi: 10.1080/07853890.2025.2525394 (PMC12224736; doi:10.1080/07853890.2025.2525394)
Supplement: TableS2.docx [file IANN_A_2525394_SM3279.docx]

TableS2: Data extraction

| Treatment | Control | Population | Outcome | Total eligible MA | Included MA | Sample size intervention/control | MA metric | Estimates [95% CI] | No. of studies | Effects model | I2; Q test P value | Egger test P value | Funnel plot | AMSTAR | Evidence class | | GRADE |
| --- | --- | --- | --- | --- | --- | --- | --- | --- | --- | --- | --- | --- | --- | --- | --- | --- | --- |
| *Significant associations* | | | | | | | | | | | | | | | | | |
| CRT | RT alone | Anaplastic glioma | OS | 1 | Zhang 2013 | 482/481 | HR | 0.84 [0.72 to 0.98] | 4 | Random | 0; 0.882 | NA | No bias | 6 | NS | | Moderate |
| CRT | RT alone | Anaplastic glioma | PFS | 1 | Zhang 2013 | 482/481 | HR | 0.68 [0.57 to 0.82] | 4 | Random | 0; 0.939 | NA | No bias | 6 | NS | | Moderate |
| GTR | STR | Adult Pilocytic Astrocytoma | Tumor recurrence | 1 | Bond 2018 | 129/123 | OR | 3.46[1.91 to 6.25] | 7 | Fixed | 31.86;0.185 | NA | No bias | 4 | Class IV | | Low |
| Resection | Biopsy | Butterfly glioblastoma | OS | 3 | Soliman 2022 | 607/1095 | OR | 2.64[1.43 to 4.87] | 13 | Random | 63;<0.01 | NA | NA | 7 | Class IV | | Very low |
| 5-aminolevulinic acid (5-ALA)–guided surgical resection | Conventional microsurgical resection | High-grade gliomas | GTR | 1 | Gandhi 2019 | 382/326 | OR | 3.785[2.647 to 5.412] | 4 | Fixed | 0;0.57 | NA | NA | 5 | Class IV | | Low |
| 5-aminolevulinic acid (5-ALA)–guided surgical resection | Conventional microsurgical resection | High-grade gliomas | Mean difference in OS | 1 | Gandhi 2019 | NA | MD | 3.056[2.435 to 3.678] | 7 | Fixed | 0;0.47 | NA | NA | 5 | Class IV | | Very low |
| 5-aminolevulinic acid (5-ALA)–guided surgical resection | Conventional microsurgical resection | High-grade gliomas | PFS | 1 | Gandhi 2019 | NA | MD | 1.031[0.613 to 1.449] | 4 | Random | 96.3;< 0.001 | NA | NA | 5 | Class IV | | Very low |
| Resection | Biopsy | Elderly patients (≥60 y) with high-grade gliomas | OS | 1 | Almenawer 2015 | 357/236 | MD | 3.886[2.143 to 5.629] | 9 | Random | 67.6;<0 .001 | 0.41 | 0.41 | 8 | Class IV | | Very low |
| Resection | Biopsy | Elderly patients (≥60 y) with high-grade gliomas | Postoperative KPS | 1 | Almenawer 2015 | 269/165 | MD | 10.403[6.581 to 14.225] | 4 | Random | 59.9;<0.001 | 0.67 | 0.67 | 8 | Class IV | | Very low |
| Resection | Biopsy | Elderly patients (≥60 y) with high-grade gliomas | PFS | 1 | Almenawer 2015 | 256/163 | MD | 2.442[1.451 to 3.433] | 4 | Random | 41.6;<0.001 | 0.75 | 0.75 | 8 | Class IV | | Low |
| Resection | Biopsy | Elderly patients (≥60 y) with high-grade gliomas | Mortality | 1 | Almenawer 2015 | 417/420 | RR | 0.275[0.122 to 0.618] | 8 | Random | 0;0.002 | 0.63 | 0.63 | 8 | NS | | Low |
| STR | Biopsy | Elderly patients (≥60 y) with high-grade gliomas | OS | 1 | Almenawer 2015 | 252/474 | MD | 2.553[0.912 to 4.195] | 11 | Random | 68.4;0.002 | 0.67 | 0.67 | 8 | Class IV | | Very low |
| GTR | Biopsy | Elderly patients (≥60 y) with high-grade gliomas | OS | 1 | Almenawer 2015 | 188/464 | MD | 7.056[4.178 to 9.934] | 10 | Random | 82.1;<0.001 | 0.22 | 0.22 | 8 | Class IV | | Very low |
| GTR | STR | Elderly patients (≥60 y) with high-grade gliomas | OS | 2 | Almenawer 2015 | 238/314 | MD | 3.774[2.255 to 5.249] | 11 | Random | 19.6;<0.001 | 0.02 | 0.02 | 8 | Class IV | | Very low |
| Stupp regimen (6 cycles of TMZ) | Long-term therapy with TMZ (>6 cycles) | High-grade gliomas | OS | 1 | Xu 2017 | 193/203 | HR | 2.39[1.82 to 3.14] | 6 | Fixed | 0;0.66 | 0.358 | 0.358 | 8 | Class IV | | Moderate |
| Stupp regimen (6 cycles of TMZ) | Long-term therapy with TMZ (>6 cycles) | High-grade gliomas | PFS | 1 | Xu 2017 | 127/113 | HR | 2.12[1.56 to 2.89] | 4 | Fixed | 0;0.44 | 0.419 | 0.419 | 8 | Class IV | | Moderate |
| Stupp regimen (6 cycles of TMZ) | Long-term therapy with TMZ (>6 cycles) | High-grade gliomas | Side-effects | 1 | Xu 2017 | 170/174 | RR | 4.47[2.78 to 7.20] | 5 | Fixed | 44;0.13 | NA | NA | 8 | Class IV | | Moderate |
| BV plus RT/TMZ | RT/TMZ | High-grade gliomas | PFS | 1 | Fu 2016 | 848/890 | HR | 0.74[0.62 to 0.88] | 3 | Random | 57;0.10 | NA | NA | 6 | Class IV | | Moderate |
| DC vaccination | Standard therapy | High-grade gliomas | OS | 3 | Vatu 2019 | 105/266 | HR | 0.65[0.53 to 0.79] | 8 | Fixed | 11;0.36 | NA | No bias | 5 | Class IV | | Low |
| DC vaccination | Standard therapy | High-grade gliomas | PFS | 3 | Vatu 2019 | 37/125 | HR | 0.59[0.39 to 0.90] | 3 | Fixed | 0;0.41 | NA | No bias | 5 | Class IV | | Very low |
| Viral therapy | Standard therapy | High-grade gliomas | OS | 2 | Vatu 2019 | 276/446 | HR | 0.81[0.71 to 0.92] | 6 | Fixed | 70;0.005 | NA | No bias | 5 | Class IV | | Very low |
| DC vaccination | Control therapy | High-grade gliomas | 1-years OS | 3 | Li 2018 | 307/637 | RR | 1.22[1.05 to 1.42] | 13 | Fixed | 0;0.995 | 0.451 | 0.451 | 8 | Class IV | | very low |
| DC vaccination | Control therapy | High-grade gliomas | 2-years OS | 3 | Li 2018 | 288/616 | RR | 1.79[1.37 to 2.35] | 12 | Fixed | 0;0.764 | >0.05 | >0.05 | 8 | Class IV | | Low |
| DC vaccination | Control therapy | High-grade gliomas | 3-years OS | 3 | Li 2018 | 268/594 | RR | 2.75[1.78 to 4.24] | 10 | Fixed | 39.2;0.097 | >0.05 | >0.05 | 8 | Class IV | | Moderate |
| DC vaccination | Control therapy | High-grade gliomas | 4-years OS | 3 | Li 2018 | 229/514 | RR | 4.53[2.43 to 8.46] | 9 | Fixed | 0;0.722 | >0.05 | >0.05 | 8 | Class IV | | Moderate |
| DC vaccination | Control therapy | High-grade gliomas | 5-years OS | 3 | Li 2018 | 180/458 | RR | 4.80[2.28 to 10.11] | 6 | Fixed | 22.3;0.266 | >0.05 | >0.05 | 8 | Class IV | | Moderate |
| DC vaccination | Control therapy | High-grade gliomas | 2-years PFS | 3 | Li 2018 | 48/148 | RR | 8.59[2.94 to 25.08] | 4 | Fixed | 0;0.940 | >0.05 | >0.05 | 8 | Class IV | | Moderate |
| DC vaccination | Control therapy | High-grade gliomas | 3-years PFS | 3 | Li 2018 | 41/138 | RR | 9.30[1.92 to 44.97] | 3 | Fixed | 0;0.780 | >0.05 | >0.05 | 8 | Class IV | | Low |
| DC vaccination | Control therapy | High-grade gliomas | 4-years PFS | 3 | Li 2018 | 29/39 | RR | 8.02[1.11 to 57.95] | 2 | Fixed | 0;0.565 | >0.05 | >0.05 | 8 | Class IV | | Low |
| CRT | RT alone | High-grade gliomas | PFS | 1 | Stewart 2002 | 1136/886 | HR | 0.83[0.75 to 0.91] | 8 | Fixed | NA | NA | NA | 7 | Class IV | | Moderate |
| Gene therapy | Standard treatment | High-grade gliomas | Median survival time | 1 | Zhao 2014 | 257/251 | MD | 0.59[0.41 to 0.76] | 3 | Fixed | 0;0.34 | NA | NA | 6 | Class IV | | High |
| Carmustine Wafers | Stupp Regimen | High-grade gliomas | OS | 1 | Ricciardi 2022 | 525/753 | MD | 2.64[0.85 to 4.44] | 4 | Random | 0;0.45 | NA | NA | 6 | Class IV | | Low |
| Combination of immunotherapy and SOC | SOC alone | High-grade gliomas | OS | 2 | Guo 2023 | 515/655 | HR | 0.74[0.56 to 0.99] | 11 | Random | 65;<0.01 | NA | Bias | 9 | Class IV | | Very low |
| Combination of immunotherapy and SOC | SOC alone | High-grade gliomas | PFS | 2 | Guo 2023 | 152/245 | HR | 0.67[0.45 to 0.99] | 6 | Random | 56;0.05 | NA | No bias | 9 | Class IV | | Very low |
| Combination of immunotherapy and SOC | SOC alone | High-grade gliomas | Adverse events | 2 | Guo 2023 | 5970/6069 | RR | 1.67[1.28 to 2.19] | 75 | Random | 79;<0.01 | NA | NA | 9 | Class IV | | Very low |
| Combination of DC therapy and SOC | SOC alone | High-grade gliomas | OS | 2 | Guo 2023 | 90(total) | HR | 0.38[0.21 to 0.68] | 3 | Random | 0;0.58 | NA | No bias | 9 | Class IV | | Very low |
| Combination of viral therapy and SOC | SOC alone | High-grade gliomas | Adverse events | 2 | Guo 2023 | 4746/4782 | RR | 1.37[1.10 to 1.70] | 45 | Random | 0;0.099 | NA | NA | 9 | Class IV | | Very low |
| Combination of immunopotentiators and SOC | SOC alone | High-grade gliomas | Adverse events | 2 | Guo 2023 | 1206/1314 | RR | 2.03[1.27 to 3.24] | 29 | Random | 92;<0.01 | NA | NA | 9 | Class IV | | Very low |
| Viral therapy with optimized injection methods combined with SOC | SOC alone | High-grade gliomas | OS | 2 | Guo 2023 | 148/279 | HR | 0.58[0.41 to 0.82] | 4 | Random | 26;0.26 | NA | Bias | 9 | Class IV | | Low |
| Ventricular entry during surgery | Without ventricular entry during surgery | High-grade gliomas | Incidences of leptomeningeal dissemination | 1 | Mistry 2018 | 410/847 | OR | 3.91[1.89 to 8.10] | 9 | Random | 41;0.11 | > 0.05 | > 0.05 | 7 | Class IV | | Moderate |
| Ventricular entry during surgery | Without ventricular entry during surgery | High-grade gliomas | Incidences of hydrocephalus | 1 | Mistry 2018 | 431/565 | OR | 7.78[3.77 to 16.05] | 11 | Random | 0;0.82 | > 0.05 | > 0.05 | 7 | Class IV | | Moderate |
| TMZ and RT | RT alone | High-grade gliomas | OS | 1 | Hart 2013 | 373/372 | HR | 0.60[0.46 to 0.79] | 3 | Random | 55.33;0.11 | NA | No bias | 9 | Class IV | | Moderate |
| TMZ and RT | RT alone | High-grade gliomas | PFS | 1 | Hart 2013 | 373/372 | HR | 0.63[0.43 to 0.92] | 3 | Random | 70.68;0.03 | NA | No bias | 9 | Class IV | | Low |
| TMZ | Standard CT | High-grade gliomas | Adverse events | 1 | Hart 2013 | 1176/1177 | OR | 2.76[2.02 to 3.77] | 5 | Fixed | 85.89;<0.0001 | NA | No bias | 9 | Class IV | | High |
| TMZ | RT alone | Elderly patients (≥60 y) with high-grade gliomas | Adverse Events | 1 | Hart 2013 | 2475/3022 | OR | 3.18[1.81 to 5.58] | 17 | Random | 87;<0.0001 | NA | No bias | 9 | Class IV | | High |
| Dose‐dense TMZ | Metronomic TMZ high-grade gliomas | High-grade gliomas | Adverse events | 1 | Hart 2013 | 186/201 | OR | 2.12[1.15 to 3.91] | 6 | Fixed | 62.35;0.02 | NA | No bias | 9 | Class IV | | Moderate |
| Fluorescein-guided surgery | Standard resection without fluorescein guidance | High-grade gliomas | GTR | 2 | Smith 2021 | 449/379 | OR | 3.983[2.754 to 5.760] | 10 | Fixed | 15.83;0.297 | 0.156 | 0.156 | 8 | Class IV | | Low |
| Combination therapy | Systemic therapy | Recurrent high-grade glioma | OS | 1 | Marwah 2023 | 269/268 | HR | 0.73[0.56 to 0.95] | 7 | Random | 35;0.16 | NA | No bias | 10 | Class IV | | Very low |
| Combination therapy | Systemic therapy | Recurrent high-grade glioma | PFS | 1 | Marwah 2023 | 194/208 | HR | 0.57[0.41 to 0.79] | 5 | Random | 55;0.07 | NA | No bias | 10 | Class IV | | Very low |
| Combination therapy | Reirradiation | Recurrent high-grade glioma | OS | 1 | Marwah 2023 | 249/222 | HR | 0.69[0.52 to 0.93] | 7 | Random | 18;0.29 | NA | No bias | 10 | Class IV | | Very low |
| Combination therapy | Reirradiation | Recurrent high-grade glioma | PFS | 1 | Marwah 2023 | 127/109 | HR | 0.52[0.38 to 0.72] | 4 | Random | 0;0.96 | NA | No bias | 10 | Class IV | | Low |
| BV-based combination therapy | Reirradiation with/without non-BV-based systemic therapy | Recurrent high-grade glioma | OS | 1 | Marwah 2023 | 143/113 | HR | 0.42[0.24 to 0.72] | 5 | Random | 38;0.17 | NA | No bias | 10 | Class IV | | Very low |
| BV-based combination therapy | Reirradiation with/without non-BV-based systemic therapy | Recurrent high-grade glioma | PFS | 1 | Marwah 2023 | 44/60 | HR | 0.46[0.27 to 0.77] | 2 | Random | 0;0.95 | NA | No bias | 10 | Class IV | | Very low |
| BV-based combination therapy | Reirradiation with/without non-BV-based systemic therapy | Recurrent high-grade glioma | Toxicity-Radiation Necrosis | 1 | Marwah 2023 | 264/89 | HR | 0.17[0.06 to 0.48] | 5 | Random | 25;0.25 | NA | Bias | 10 | Class IV | | Low |
| BV-Based Combination Therapy | Systemic Therapy | Recurrent high-grade glioma | OS | 1 | Marwah 2023 | 241/237 | HR | 0.74[0.56 to 0.99] | 6 | Random | 44;0.11 | NA | No bias | 10 | Class IV | | Very low |
| BV-Based Combination Therapy | Systemic Therapy | Recurrent high-grade glioma | PFS | 1 | Marwah 2023 | 166/177 | HR | 0.57[0.38 to 0.86] | 4 | Random | 64;0.04 | NA | No bias | 10 | Class IV | | Very low |
| Reirradiation | Systemic therapy | Recurrent glioblastoma | OS | 1 | Marwah 2023 | 65/172 | HR | 0.94[0.67 to 1.31] | 3 | Random | 0;0.48 | NA | No bias | 10 | NS | | Very low |
| Reirradiation | Systemic therapy | Recurrent glioblastoma | PFS | 1 | Marwah 2023 | 50/135 | HR | 0.87[0.61 to 1.22] | 2 | Random | 0;0.64 | NA | No bias | 10 | NS | | Very low |
| Combination therapy | Systemic therapy | Recurrent glioblastoma | OS | 1 | Marwah 2023 | 114/115 | HR | 0.90[0.62 to 1.32] | 2 | Random | 11;0.29 | NA | No bias | 10 | NS | | Very low |
| Combination therapy | Systemic therapy | Recurrent glioblastoma | PFS | 1 | Marwah 2023 | 114/115 | HR | 0.66[0.49 to 0.91] | 2 | Random | 15;0.28 | NA | No bias | 10 | Class IV | | Very low |
| Combination Therapy | Reirradiation | Recurrent glioblastoma | OS | 1 | Marwah 2023 | 140/117 | HR | 0.55[0.39 to 0.76] | 5 | Random | 0;0.89 | NA | No bias | 10 | Class IV | | Low |
| Combination Therapy | Reirradiation | Recurrent glioblastoma | PFS | 1 | Marwah 2023 | 127/109 | HR | 0.52[0.38 to 0.72] | 4 | Random | 0;0.96 | NA | No bias | 10 | Class IV | | Low |
| BV-based combination therapy | Reirradiation with/without non-BV-based systemic therapy | Recurrent glioblastoma | OS | 1 | Marwah 2023 | 82/107 | HR | 0.34[0.21 to 0.55] | 4 | Random | 0;0.47 | NA | No bias | 10 | Class IV | | Moderate |
| BV-based combination therapy | Reirradiation with/without non-BV-based systemic therapy | Recurrent glioblastoma | PFS | 1 | Marwah 2023 | 44/60 | HR | 0.46[0.27 to 0.77] | 2 | Random | 0;0.95 | NA | No bias | 10 | Class IV | | Low |
| STR | Biopsy | Low-Grade Glioma | OS | 3 | Yang 2018 | 111/181 | MD | 2.69[0.02 to 5.36] | 3 | Random | 80.4;0.04 | > 0.05 | > 0.05 | 8 | Class IV | | Very low |
| GTR | Biopsy | Low-Grade Glioma | OS | 3 | Yang 2018 | 132/159 | MD | 4.48[2.54 to 6.43] | 4 | Random | 41.3;<0.001 | > 0.05 | > 0.05 | 8 | Class IV | | Low |
| Resection of any extent | Biopsy | Low-Grade Glioma | OS | 3 | Yang 2018 | 225/508 | MD | 3.24[0.64 to 5.84] | 6 | Random | 0;0.015 | > 0.05 | > 0.05 | 8 | Class IV | | Very low |
| STR | GTR | Low-Grade Glioma | OS | 3 | Yang 2018 | 635/531 | MD | 3.87[1.76 to 5.98] | 11 | Random | 91.3;<0.001 | > 0.05 | > 0.05 | 8 | Class IV | | Very low |
| GTR | STR | Low-Grade Glioma | 5-year survival | 3 | Yang 2018 | 615/847 | RR | 1.26[1.19 to 1.34] | 14 | Random | 19;<0.0001 | > 0.05 | > 0.05 | 8 | Class IV | | low |
| GTR | STR | Low-Grade Glioma | PFS | 3 | Yang 2018 | 355/318 | MD | 2.08[0.26 to 3.89] | 5 | Random | 90.1;0.025 | > 0.05 | > 0.05 | 8 | Class IV | | Very low |
| GTR | STR | Low-Grade Glioma | Seizure control | 3 | Yang 2018 | 338/503 | RR | 1.53[1.39 to 1.68] | 6 | Random | 0;<0.0001 | > 0.05 | > 0.05 | 8 | Class IV | | Low |
| GTR | STR | Low-Grade Glioma | Malignant transformation | 3 | Yang 2018 | 76/98 | RR | 0.43[0.20 to 0.93] | 6 | Random | 30.3;0.032 | > 0.05 | > 0.05 | 8 | Class IV | | Low |
| GTR | STR | Low-Grade Glioma | Mortality at 2 years | 3 | Brown 2019 | 1207/2264 | RR | 0.29[0.17 to 0.52] | 19 | Random | 24;0.18 | NA | NA | 7 | Class IV | | Low |
| GTR | STR | Low-Grade Glioma | Mortality at 5 years | 3 | Brown 2019 | 1329/2562 | RR | 0.39[0.29 to 0.51] | 23 | Random | 40;0.03 | NA | NA | 7 | Class IV | | Low |
| GTR | STR | Low-Grade Glioma | Mortality at 10 years | 3 | Brown 2019 | 994/1997 | RR | 0.50[0.35 to 0.70] | 15 | Random | 91;<0.00001 | NA | NA | 7 | Class IV | | Very low |
| GTR | STR | Low-Grade Glioma | Progression at 2 years | 3 | Brown 2019 | 899/931 | RR | 0.37[0.24 to 0.57] | 10 | Random | 60;0.008 | NA | NA | 7 | Class IV | | Very low |
| GTR | STR | Low-Grade Glioma | Progression at 5 years | 3 | Brown 2019 | 1008/1130 | RR | 0.50[0.39 to 0.64] | 13 | Random | 79;<0.00001 | NA | NA | 7 | Class IV | | Very low |
| GTR | STR | Low-Grade Glioma | Progression at 10 years | 3 | Brown 2019 | 729/829 | RR | 0.67[0.53 to 0.84] | 8 | Random | 93;<0.00001 | NA | NA | 7 | Class IV | | Very low |
| STR | Biopsy | Low-Grade Glioma | Mortality at 2 years | 3 | Brown 2019 | 374/338 | RR | 0.53[0.33 to 0.84] | 7 | Random | 36;0.15 | NA | NA | 7 | Class IV | | Very low |
| Resection | Biopsy | Low-Grade Glioma | Mortality at 2 years | 3 | Brown 2019 | 724/487 | RR | 0.46[0.29 to 0.75] | 7 | Random | 51;0.04 | NA | NA | 7 | Class IV | | Very low |
| Resection | Biopsy | Low-Grade Glioma | Mortality at 5 years | 3 | Brown 2019 | 796/505 | RR | 0.60[0.43 to 0.84] | 8 | Random | 79;<0.00001 | NA | NA | 7 | Class IV | | Very low |
| Resection | Biopsy | Low-Grade Glioma | Mortality at 10 years | 3 | Brown 2019 | 214/176 | RR | 0.67[0.52 to 0.86] | 3 | Random | 29;0.24 | NA | NA | 7 | Class IV | | Very low |
| Early Radiation | Late/No Radiation | Low-Grade Glioma | Progression at 2 years | 3 | Brown 2019 | 831/642 | RR | 0.66[0.51 to 0.86] | 6 | Random | 51;0.07 | NA | NA | 7 | Class IV | | Very low |
| Early Radiation | Late/No Radiation | Low-Grade Glioma | Progression at 5 years | 3 | Brown 2019 | 831/642 | RR | 0.73[0.61 to 0.88] | 6 | Random | 68;0.008 | NA | NA | 7 | Class IV | | Very low |
| Early Radiation | Late/No Radiation | Low-Grade Glioma | Progression at 10 years | 3 | Brown 2019 | 661/453 | RR | 0.74[0.60 to 0.91] | 4 | Random | 89;<0.0001 | NA | NA | 7 | Class IV | | Very low |
| CT | RT | Low-Grade Glioma | Progression at 5 years | 3 | Brown 2019 | 176/255 | RR | 0.69[0.55 to 0.87] | 3 | Random | 0;0.43 | NA | NA | 7 | Class IV | | Very low |
| CT | RT | Low-Grade Glioma | Progression at 10 years | 3 | Brown 2019 | 176/255 | RR | 0.58[0.39 to 0.87] | 3 | Random | 75;0.02 | NA | NA | 7 | Class IV | | Very low |
| GTR | STR | pHGGs | 1-Year Mortality | 1 | Hatoum 2022 | 337/447 | RR | 0.69[0.56 to 0.83] | 29 | Random | 0;0.88 | 0.14 | 0.14 | 8 | Class IV | | Low |
| GTR | STR | pHGGs | 2-Year Mortality | 1 | Hatoum 2022 | 332/443 | RR | 0.74[0.67 to 0.83] | 29 | Random | 0;0.49 | > 0.05 | > 0.05 | 8 | Class IV | | Low |
| STR | Biopsy | pHGGs | 2-Year Mortality | 1 | Hatoum 2022 | 419/258 | RR | 0.89[0.82 to 0.97] | 23 | Random | 6;0.39 | > 0.05 | > 0.05 | 8 | Class IV | | Very low |
| GTR | STR | pHGGs | 6-Month Progression | 1 | Hatoum 2022 | 174/236 | RR | 0.62[0.46 to 0.82] | 17 | Random | 0;1.00 | > 0.05 | > 0.05 | 8 | Class IV | | Very low |
| GTR | STR | pHGGs | 1-Year Progression | 1 | Hatoum 2022 | 174/235 | RR | 0.74[0.60 to 0.90] | 17 | Random | 19;0.23 | > 0.05 | > 0.05 | 8 | Class IV | | Low |
| SMR | GTR | Glioblastoma | PFS | 1 | Mier-García 2023 | 191/374 | HR | 0.67[0.53 to 0.84] | 8 | Random | 0;1.00 | NA | NA | 10 | Class IV | | Very low |
| SMR | GTR | Glioblastoma | OS | 1 | Mier-García 2023 | 433/1015 | HR | 0.72[0.62 to 0.85] | 13 | Fixed | 0;0.75 | NA | NA | 10 | Class IV | | Very low |
| SMR | GTR | Glioblastoma, IDH wild-type | PFS | 1 | Mier-García 2023 | 68/189 | HR | 0.65[0.45 to 0.94] | 3 | Random | 0;0.99 | NA | NA | 10 | Class IV | | Very low |
| GTR | STR | Glioma | 1-year OS | 2 | Shi 2023 | 103/109 | RR | 1.37[1.16 to 1.62] | 13 | Random | 63.6;0.001 | 0.127 | 0.127 | 8 | Class IV | | Very low |
| GTR | STR | Glioma | 2-year OS | 2 | Shi 2023 | 49/79 | RR | 1.44[1.16 to 1.79] | 14 | Random | 71.9;0.000 | 0.381 | 0.381 | 8 | Class IV | | Very low |
| GTR | STR | Glioma | 3-year OS | 2 | Shi 2023 | 66/80 | RR | 1.33[1.19 to 1.47] | 10 | Random | 0;0.827 | 0.21 | 0.21 | 8 | Class IV | | Low |
| GTR | STR | Glioma | 5-year OS | 2 | Shi 2023 | 120/133 | RR | 1.27[1.15 to 1.40] | 35 | Random | 88.7;0.000 | 0.109 | 0.109 | 8 | Class IV | | Very low |
| GTR | STR | Glioma | 10-year OS | 2 | Shi 2023 | 173/104 | RR | 1.42[1.28 to 1.57] | 15 | Random | 43.2;0.049 | 0.044 | 0.044 | 8 | Class IV | | Low |
| GTR | STR | Glioma | 15-year OS | 2 | Shi 2023 | 108/169 | RR | 1.69[1.33 to 2.16] | 3 | Random | 41.4;0.192 | 0.077 | 0.077 | 8 | Class IV | | Low |
| GTR | STR | Glioma | OS | 2 | Shi 2023 | 66/43 | WMD | 10.15[1.25 to 19.04] | 3 | Random | 79.3;0.008 | 0.11 | 0.11 | 8 | Class IV | | Very low |
| GTR | STR | Glioma | 1-year PFS | 2 | Shi 2023 | 126/158 | RR | 1.70[1.12 to 2.59] | 3 | Random | 51.4;0.127 | 0.858 | 0.858 | 8 | Class IV | | Very low |
| GTR | STR | Glioma | 3-year PFS | 2 | Shi 2023 | 83/94 | RR | 3.27[1.57 to 6.82] | 3 | Random | 12.9;0.317 | > 0.05 | > 0.05 | 8 | Class IV | | Very low |
| GTR | STR | Glioma | 5-year PFS | 2 | Shi 2023 | 69/76 | RR | 1.42[1.22 to 1.66] | 13 | Random | 50.5;0.019 | > 0.05 | > 0.05 | 8 | Class IV | | Very low |
| GTR | STR | Glioma | Recurrence | 2 | Shi 2023 | 46/53 | RR | 0.31[0.17 to 0.58] | 13 | Random | 65.2;0.001 | > 0.05 | > 0.05 | 8 | Class IV | | Very low |
| GTR | STR | Glioma | 5-year local control | 2 | Shi 2023 | 38/55 | RR | 2.04[1.33 to 3.14] | 2 | Random | 22.5;0.256 | > 0.05 | > 0.05 | 8 | Class IV | | Low |
| GTR | STR | Glioma | Seizure control | 2 | Shi 2023 | 112/93 | RR | 1.41[1.12 to 1.78] | 4 | Random | 70.2;0.018 | > 0.05 | > 0.05 | 8 | Class IV | | Very low |
| GTR | STR | High-grade gliomas | 1-year OS | 2 | Shi 2023 | 182/192 | RR | 1.39[1.17 to 1.67] | 12 | Random | 66.8;0.001 | > 0.05 | > 0.05 | 8 | Class IV | | Very low |
| GTR | STR | Adults with low grade glioma | 1-year OS | 2 | Shi 2023 | 373/397 | RR | 1.39[1.12 to 1.73] | 9 | Random | 70.9;0.001 | > 0.05 | > 0.05 | 8 | Class IV | | Very low |
| GTR | STR | High-grade gliomas | 2-year OS | 2 | Shi 2023 | 882/625 | RR | 1.61[1.36 to 1.90] | 11 | Random | 48.5;0.035 | > 0.05 | > 0.05 | 8 | Class IV | | Low |
| GTR | STR | Adults with low grade glioma | 2-year OS | 2 | Shi 2023 | 142/110 | RR | 1.12[1.00 to 1.25] | 2 | Random | 0;0.719 | > 0.05 | > 0.05 | 8 | Class IV | | Very low |
| GTR | STR | High-grade gliomas | 3-year OS | 2 | Shi 2023 | 287/159 | RR | 1.43[1.06 to 1.92] | 5 | Random | 0;0.558 | > 0.05 | > 0.05 | 8 | Class IV | | Very low |
| GTR | STR | Adults with low grade glioma | 3-year OS | 2 | Shi 2023 | 266/137 | RR | 1.36[1.01 to 1.84] | 4 | Random | 0;0.692 | > 0.05 | > 0.05 | 8 | Class IV | | Very low |
| GTR | STR | Low-grade glioma | 5-year OS | 2 | Shi 2023 | 1192/749 | RR | 1.13[1.02 to 1.25] | 13 | Random | 87.3;0 | > 0.05 | > 0.05 | 8 | Class IV | | Very low |
| GTR | STR | High-grade gliomas | OS | 2 | Shi 2023 | 148/33 | WMD | 8.62[0.36 to 16.89] | 2 | Random | 85.7;0.008 | > 0.05 | > 0.05 | 8 | Class IV | | Very low |
| iMRI | Conventional surgery | Glioma | GTR | 2 | Lo 2021 | 795/699 | RR | 1.65[1.43 to 1.90] | 11 | Random | 4;0.40 | 0.59 | 0.59 | 8 | NS | | Very low |
| iMRI | Conventional surgery | Glioma | Mean extent of resection | 2 | Lo 2021 | 129/94 | MD | 6.12[4.23 to 8.00] | 9 | Random | 44；0.43 | 0.92 | 0.92 | 8 | Class IV | | Very low |
| Conventional surgery | iMRI | Glioma | GTR | 2 | Lo 2021 | 795/699 | RR | 1.42[1.17 to 1.73] | 3 | Random | 7;0.34 | 0.59 | 0.59 | 8 | NS | | Moderate |
| Hormone replacement therapy | RT and TMZ | Glioma | Cumulative risk | 1 | Lan 2018 | 4490/12119 | OR | 0.91[0.84 to 0.99] | 10 | Fixed | 12.4;0.322 | 0.174 | 0.174 | 9 | NS | | Very low |
| Oral contraceptives | RT and TMZ | Glioma | Cumulative risk | 1 | Lan 2018 | 2899131(total) | RR | 0.75[0.66 to 0.84] | 4 | Fixed | 0;0.899 | 0.785 | 0.785 | 9 | NS | | Very low |
| Immunotherapy | Standard care treatments(included surgical resection, RT, or CT) | Glioma | 1-year OS | 1 | Hanaei 2018 | 425/269 | HR | 0.69[0.52 to 0.92] | 7 | Random | 0;0.43 | NA | No bias | 7 | Class IV | | Very low |
| Immunotherapy | Standard care treatments(included surgical resection, RT, or CT) | Glioma | 2-year OS | 1 | Hanaei 2018 | 425/330 | HR | 0.83[0.69 to 0.99] | 8 | Random | 0;0.61 | NA | No bias | 7 | Class IV | | Very low |
| Active immunotherapy | Standard care treatments(included surgical resection, RT, or CT) | Glioma | 3-year OS | 1 | Hanaei 2018 | 73/159 | HR | 0.67[0.48 to 0.92] | 3 | Fixed | 0;0.78 | NA | No bias | 7 | Class IV | | Very low |
| Immunotherapy | Standard care treatments(included surgical resection, RT, or CT) | Glioma | PFS | 1 | Hanaei 2018 | 929/1013 | MD | 0.323[0.110 to 0.536] | 9 | Random | NA | NA | No bias | 7 | Class IV | | Very low |
| Awake craniotomy | General anesthetic resection | Glioma | Late neurological deficit | 1 | Bu 2021 | 479/314 | RR | 0.53[0.33 to 0.87] | 9 | Random | 32;0.17 | NA | No bias | 8 | Class IV | | Very low |
| Integrated Traditional Chinese and Western medicine | Western medical treatment | Glioma | Efficacy | 1 | Yu 2021 | 265/263 | RR | 1.32[1.18 to 1.49] | 8 | Fixed | 0;0.89 | > 0.05 | > 0.05 | 8 | Class IV | | High |
| Integrated Traditional Chinese and Western medicine | Western medical treatment | Glioma | Complete remission/partial remission/stable disease/progress disease | 1 | Yu 2021 | 136/134 | OR | 2.011[1.28 to 3.15] | 5 | Fixed | 0;0.911 | > 0.05 | > 0.05 | 8 | Class IV | | Moderate |
| Integrated Traditional Chinese and Western medicine | Western medical treatment | Glioma | Bone marrow suppression | 1 | Yu 2021 | 217/217 | RR | 0.44[0.28 to 0.68] | 7 | Fixed | 7;0.37 | > 0.05 | > 0.05 | 8 | Class IV | | High |
| Integrated Traditional Chinese and Western medicine | Western medical treatment | Glioma | Gastrointestinal adverse reactions | 1 | Yu 2021 | 217/217 | RR | 0.42[0.30 to 0.60] | 7 | Fixed | 0;0.45 | > 0.05 | > 0.05 | 8 | Class IV | | High |
| Integrated Traditional Chinese and Western medicine | Western medical treatment | Glioma | Two-year survival time | 1 | Yu 2021 | 172/169 | RR | 1.74[1.47 to 2.05] | 4 | Fixed | 47;0.13 | > 0.05 | > 0.05 | 8 | Class IV | | High |
| Integrated Traditional Chinese and Western medicine | Western medical treatment | Glioma | Three-year survival time | 1 | Yu 2021 | 90/90 | RR | 20.33[4.03 to 102.59] | 3 | Fixed | 43;0.19 | > 0.05 | > 0.05 | 8 | Class IV | | High |
| Targeted therapy(including Regorafenib or Galunisertib or Depatux-M or Enzastaurin or Cediranib) plus Lomustine | Lomustine | Glioblastoma | PFS | 1 | Ippen 2024 | 566/362 | RR | 0.65[0.53 to 0.80] | 4 | Random | 23.38;0 | 0.4551 | 0.4551 | 7 | Class IV | | High |
| Targeted therapy | TMZ | Glioblastoma | PFS | 1 | Scherm 2023 | 2024/1917 | RR | 0.83[0.74 to 0.94] | 12 | Random | 60.02;0.0024 | 0.5068 | 0.5068 | 7 | Class IV | | Low |
| Molecularly targeted drugs | TMZ plus RT | Glioblastoma | PFS | 1 | Wang 2019 | 1734/1629 | OR | 0.86[0.75 to 0.98] | 13 | Random | 64;0.0009 | > 0.05 | > 0.05 | 8 | Class IV | | Low |
| Molecularly targeted drugs | TMZ plus RT | Glioblastoma | Adverse events | 1 | Wang 2019 | 1073/1357 | OR | 1.68[1.44 to 1.97] | 7 | Fixed | 37;0.15 | > 0.05 | > 0.05 | 8 | Class IV | | High |
| Antiangiogenic drugs | Cytotoxic drug alone | Glioblastoma | PFS | 1 | Lombardi 2017 | 4113(total) | HR | 0.76[0.65 to 0.89] | 15 | Random | 76.04;<0.001 | NA | No bias | 8 | Class IV | | Moderate |
| Carmustine implantation | Non-carmustine-treated | Glioblastoma | OS | 1 | Xiao 2020 | 1871/3429 | HR | 0.85[0.79 to 0.92] | 21 | Fixed | 45;0.02 | NA | No bias | 8 | Class IV | | Low |
| Carmustine implantation | Non-carmustine-treated | Glioblastoma | PFS | 1 | Xiao 2020 | 942/1648 | HR | 0.85[0.77 to 0.94] | 8 | Fixed | 67;0.004 | NA | No bias | 8 | Class IV | | Very low |
| Carmustine implantation | Non-carmustine-treated | Different pathological grades of glioma | OS | 1 | Xiao 2020 | 1856/3422 | HR | 0.85[0.79 to 0.91] | 20 | Fixed | 24;0.16 | NA | No bias | 8 | Class IV | | Low |
| Carmustine implantation | TMZ CT and TMZ plus carmustine CT | Glioblastoma | OS | 1 | Xiao 2020 | 226/377 | HR | 0.78[0.63 to 0.97] | 2 | Fixed | 0;0.83 | NA | No bias | 8 | Class IV | | Very low |
| Standard therapy + Anti-vascular endothelial growth factor | Standard therapy | Glioblastoma | PFS | 1 | Xiao 2018 | 1034/867 | OR | 0.76[0.65 to 0.89] | 5 | Random | 50;0.09 | NA | NA | 7 | Class IV | | High |
| Standard therapy + BV | Standard therapy | Glioblastoma | PFS | 1 | Xiao 2018 | 487/385 | OR | 0.75[0.65 to 0.87] | 3 | Random | 0;0.54 | NA | NA | 7 | Class IV | | High |
| Molecularly targeted drugs combined with TMZ plus RT | TMZ plus RT | Glioblastoma | PFS | 1 | Su 2016 | 1361/1276 | HR | 0.796[0.701 to 0.903] | 7 | Random | 46.513;<0.001 | NA | No bias | 8 | Class IV | | High |
| Molecularly targeted drugs combined with TMZ plus RT | TMZ plus RT | MGMT-nonmethylated patients | PFS | 1 | Su 2016 | 866/869 | HR | 0.604[0.511 to 0.715] | 3 | Fixed | 13.451;0 | NA | No bias | 8 | Class IV | | High |
| BV combined with TMZ plus RT | TMZ plus RT | Glioblastoma | OS | 1 | Su 2016 | 778/780 | HR | 0.722[0.612 to 0.853] | 2 | Random | 75.369;0 | NA | No bias | 8 | Class IV | | Moderate |
| Cilengitide | TMZ plus RT | Glioblastoma | PFS | 1 | Su 2016 | 251/163 | HR | 0.792[0.642 to 0.977] | 3 | Fixed | 0;0.030 | NA | No bias | 8 | NS | | Moderate |
| Molecularly targeted drugs combined with TMZ plus RT | TMZ plus RT | Glioblastoma | Adverse effects | 1 | Su 2016 | 1361/1276 | OR | 1.679[1.434 to 1.967] | 7 | Fixed | 36.615;<0.001 | NA | No bias | 8 | Class IV | | High |
| BV combined with TMZ | TMZ alone | Glioma or glioblastoma | PFS | 1 | Wei 2024 | 1412/1480 | HR | 0.64[0.60 to 0.68] | 7 | Fixed | 26;0.23 | NA | Bias | 8 | Class IV | | Moderate |
| BV combined with TMZ | TMZ alone | Glioma or glioblastoma | Overall complete remission rate | 1 | Wei 2024 | 99/96 | OR | 3.78[2.00 to 7.15] | 2 | Fixed | 0;0.49 | NA | Bias | 8 | Class IV | | High |
| BV combined with TMZ | TMZ alone | Glioma or glioblastoma | OS | 1 | Wei 2024 | 1412/1480 | HR | 0.92[0.86 to 0.98] | 7 | Fixed | 87;<0.0001 | NA | Bias | 8 | Class IV | | Very low |
| BV combined with TMZ | TMZ alone | Glioma or glioblastoma | Adverse events | 1 | Wei 2024 | 1408/1395 | OR | 2.52[1.85 to 3.43] | 6 | Random | 64;0.02 | NA | Bias | 8 | Class IV | | Moderate |
| Levetiracetam plus SOC | SOC | Glioblastoma | OS | 1 | Chen 2022 | 1199/1540 | MD | 6.21[2.75 to 9.66] | 6 | Random | 100;0 | NA | Bias | 8 | Class IV | | Very low |
| Supratotal resection | GTR | Glioblastoma | OS | 3 | Aziz 2023 | 3088/2762 | HR | 0.78[0.72 to 0.85] | 11 | Random | 96;<0.00001 | > 0.05 | > 0.05 | 9 | Class IV | | Very low |
| Supratotal resection | GTR | Glioblastoma | PFS | 3 | Aziz 2023 | 142/198 | HR | 0.20[0.07 to 0.56] | 4 | Random | 88;<0.00001 | > 0.05 | > 0.05 | 9 | Class IV | | Very low |
| Supratotal resection | STR or GTR | Glioblastoma | PFS | 3 | Aziz 2023 | 333/323 | MD | 1.14[0.97 to 1.31] | 6 | Fixed | 93;<0.00001 | >0.05 | >0.05 | 9 | Class IV | | Very low |
| Tumor Treating Fields therapy plus SOC | SOC alone | Newly diagnosed glioblastoma | OS | 2 | Ballo 2023 | 748/682 | HR | 0.63[0.53 to 0.75] | 7 | Random | 21;0.27 | NA | NA | 7 | Class IV | | Low |
| ≥ 75% device usage rate threshold | <75% device usage rate threshold | Newly diagnosed glioblastoma | OS | 2 | Ballo 2023 | 514/501 | HR | 0.60[0.48 to 0.73] | 5 | Random | 15;<0.001 | NA | NA | 7 | Class IV | | Low |
| BV in combination with CRT(including TMZ or Lomustine) | CRT alone(including TMZ or Lomustine) | Glioblastoma | PFS | 1 | Lan 2022 | 2360/1261 | HR | 0.64[0.58 to 0.70] | 7 | Fixed | 62;0.02 | NA | No bias | 7 | Class IV | | Moderate |
| Combination therapy of BV plus CT | BV or CT alone | Glioblastoma | PFS | 1 | Yang 2017 | 607(total) | HR | 0.66[0.56 to 0.78] | 4 | Fixed | 48;0.12 | NA | NA | 8 | Class IV | | Moderate |
| Combination therapy of BV plus CT | BV or CT alone | Glioblastoma | Object response rate | 1 | Yang 2017 | 189/236 | OR | 1.85[1.17 to 2.93] | 3 | Fixed | 0;0.74 | NA | NA | 8 | Class IV | | Low |
| Combination therapy of BV plus CT | BV or CT alone | Glioblastoma | Adverse events | 1 | Yang 2017 | 137/146 | OR | 1.75[1.09 to 2.83] | 2 | Fixed | 21;0.26 | NA | NA | 8 | Class IV | | Low |
| CRT | RT | Glioblastoma | OS | 1 | Wang 2017 | 572/566 | HR | 0.70[0.56 to 0.88] | 5 | Random | 53;0.07 | NA | NA | 7 | Class IV | | Low |
| RT plus TMZ | RT alone | Glioblastoma | Survival rate | 4 | Zhao 2021 | 625/443 | HR | 0.63[0.52 to 0.76] | 3 | Random | 37;0.20 | NA | NA | 7 | Class IV | | High |
| RT plus TMZ | RT alone | Glioblastoma | PFS | 4 | Zhao 2021 | 315/316 | RR | 3.25[2.13 to 4.94] | 2 | Random | 0;0.99 | NA | NA | 7 | Class IV | | High |
| RT plus TMZ | RT alone | Glioblastoma | Adverse events | 4 | Zhao 2021 | 315/315 | RR | 3.58[1.10 to 11.59] | 2 | Random | 0;0.94 | NA | NA | 7 | Class IV | | High |
| Anti-EGFR therapies with CT | SOC with CT | Recurrent glioblastoma | PFS | 1 | Lee 2020 | 275(total) | HR | 0.75 [0.58 to 0.96] | 3 | Random | 0;0.97 | NA | No bias | 9 | Class IV | | Moderate |
| GTR | STR | Glioblastoma | 1-Year Mortality | 2 | Brown 2016 | 8297/12472 | RR | 0.62[0.56 to 0.69] | 25 | Random | 67;<0.001 | NA | Bias | 8 | Class III | | Very low |
| GTR | STR | Glioblastoma | 2-Year Mortality | 2 | Brown 2016 | 8284/12415 | RR | 0.84[0.79 to 0.89] | 23 | Random | 86;<0.001 | NA | Bias | 8 | Class III | | Very low |
| STR | Biopsy | Glioblastoma | 1-Year Mortality | 2 | Brown 2016 | 10451/3685 | RR | 0.85[0.80 to 0.91] | 20 | Random | 58;<0.001 | NA | Bias | 8 | Class IV | | Very low |
| Resection | Biopsy | Glioblastoma | 1-Year Mortality | 2 | Brown 2016 | 16036/3724 | RR | 0.77[0.71 to 0.84] | 21 | Random | 76;<0.0001 | NA | Bias | 8 | Class IV | | Very low |
| GTR | STR | Glioblastoma | Progression at one year | 2 | Brown 2016 | 218/270 | RR | 0.66[0.43 to 0.99] | 5 | Random | 83;<0.0001 | NA | Bias | 8 | Class IV | | Very low |
| Resection | Biopsy | Glioblastoma | Progression at one year | 2 | Brown 2016 | 751/299 | RR | 0.61[0.44 to 0.84] | 7 | Random | 83;<0.0001 | NA | Bias | 8 | Class IV | | Very low |
| BV plus other CRT | Other CRT | Recurrent glioblastoma | Median PFS | 1 | LI 2016 | 1016/996 | HR | 0.73[0.62 to 0.86] | 5 | Random | 52;0.08 | > 0.05 | > 0.05 | 10 | Class IV | | Moderate |
| BV plus other CRT | Other CRT | Recurrent glioblastoma | PFS rate | 1 | LI 2016 | 3300/3269 | OR | 2.01[1.54 to 2.63] | 22 | Random | 70;<0.00001 | > 0.05 | > 0.05 | 10 | Class IV | | High |
| BV plus other CRT | Other CRT | Recurrent glioblastoma | OS rate | 1 | LI 2016 | 3851/3762 | OR | 1.17[1.04 to 1.32] | 23 | Fixed | 21;0.18 | > 0.05 | > 0.05 | 10 | Class IV | | High |
| BV plus some specific cytotoxic treatments | A certain cytotoxic treatment | Recurrent glioblastoma | Objective response rate | 1 | Zhang 2021 | 292/257 | RR | 3.46[2.15 to 5.58] | 3 | Random | 0;0.49 | NA | NA | 7 | Class IV | | High |
| BV plus some specific cytotoxic treatments | A certain cytotoxic treatment | Recurrent glioblastoma | Median PFS | 1 | Zhang 2021 | 248/217 | MD | 0.71[0.28 to 1.15] | 4 | Random | 0;0.40 | NA | NA | 7 | Class IV | | Moderate |
| BV plus some specific cytotoxic treatments | A certain cytotoxic treatment | Recurrent glioblastoma | Hypertension | 1 | Zhang 2021 | 413/394 | RR | 2.68[1.26 to 5.67] | 5 | Random | 74;0.004 | NA | NA | 7 | Class IV | | Moderate |
| TMZ with hypofractionated radiation therapy | TMZ with standard radiation therapy | Elderly glioblastoma | Median OS | 1 | Lu 2019 | 592/325 | MD | -3.47[-6.33 to -0.60] | 7 | Random | 98.9;0.02 | 0.35 | 0.35 | 9 | Class IV | | Very low |
| TMZ alone | RT alone | Elderly glioblastoma | Methylated tumors | 1 | Yin 2014 | 330/462 | HR | 0.66[0.47 to 0.93] | 3 | Fixed | 0;0.94 | > 0.05 | > 0.05 | 8 | Class IV | | Very low |
| TMZ alone | RT alone | Elderly glioblastoma | Unmethylated tumors | 1 | Yin 2014 | 314/401 | HR | 1.32[1.00 to 1.76] | 2 | Fixed | 0;0.35 | > 0.05 | > 0.05 | 8 | NS | | Very low |
| GTR | STR | Glioblastoma, IDH–wild-type | OS | 1 | Jusue-Torres 2023 | 1321(total) | MD | 7.58[4.29 to 10.88] | 5 | Random | 45.5;0.13 | NA | No bias | 9 | Class IV | | Very low |
| GTR | Biopsy | Glioblastoma, IDH–wild-type | OS | 1 | Jusue-Torres 2023 | 779(total) | MD | 12.42[7.02 to 17.83] | 2 | Random | 76.5;0.04 | NA | No bias | 9 | NS | | Very low |
| STR | Biopsy | Glioblastoma, IDH–wild-type | OS | 1 | Jusue-Torres 2023 | 779(total) | MD | 5.12[3.33 to 6.92] | 2 | Random | 0;0.97 | NA | No bias | 9 | NS | | Very low |
| GTR | STR | Glioblastoma, IDH–wild-type | PFS | 1 | Jusue-Torres 2023 | 1093(total) | MD | 3.88[2.13 to 5.64] | 4 | Random | 74.7;<0.01 | NA | No bias | 9 | Class IV | | Very low |
| GTR | STR | Glioblastoma, IDH–wild-type | OS | 1 | Jusue-Torres 2023 | 864(total) | HR | 0.49[0.36 to 0.65] | 4 | Random | 0;0.46 | NA | No bias | 9 | NS | | Very low |
| GTR | Biopsy | Glioblastoma, IDH–wild-type | OS | 1 | Jusue-Torres 2023 | 1159(total) | HR | 0.39[0.29 to 0.51] | 5 | Random | 10.3;0.55 | NA | No bias | 9 | Class IV | | Low |
| GTR | STR or biopsy | Glioblastoma, IDH–wild-type | OS | 1 | Jusue-Torres 2023 | 2023(total) | HR | 0.43[0.35 to 0.53] | 9 | Random | 14.1;0.52 | NA | No bias | 9 | Class IV | | Low |
| GTR | STR | Glioblastoma, IDH–wild-type | PFS | 1 | Jusue-Torres 2023 | 497(total) | HR | 0.58[0.39 to 0.87] | 2 | Random | 0;0.72 | NA | No bias | 9 | Class IV | | Very low |
| GTR | Biopsy | Glioblastoma, IDH–wild-type | PFS | 1 | Jusue-Torres 2023 | 945(total) | HR | 0.41[0.30 to 0.57] | 3 | Random | 0;0.45 | NA | No bias | 9 | NS | | Very low |
| GTR | STR/biopsy | Glioblastoma, IDH–wild-type | PFS | 1 | Jusue-Torres 2023 | 1442(total) | HR | 0.47[0.37 to 0.60] | 5 | Random | 0;0.48 | NA | No bias | 9 | Class IV | | Low |
| Intra-arterial CT | Intravenous CT | Malignant glioma | Leukocytopenia | 1 | Chen 2013 | 69/68 | RR | 0.31[0.15 to 0.64] | 3 | Fixed | 0;0.87 | NA | NA | 8 | Class IV | | Moderate |
| Vaccines | Conventional treatments | Malignant glioma | OS rate for 2 years | 1 | Jajin 2024 | 270/5906 | RR | 2.28[1.67 to 3.12] | 13 | Fixed | 52.3;<0.0001 | > 0.05 | > 0.05 | 8 | Class IV | | Low |
| Vaccines | Conventional treatments | Malignant glioma | PFS rate for 2 years | 1 | Jajin 2024 | 159/5758 | RR | 2.87[1.63 to 5.03] | 9 | Fixed | 83.3;<0.001 | > 0.05 | > 0.05 | 8 | Class IV | | Low |
| Vaccines | Conventional treatments | Malignant glioma | Survival duration | 1 | Jajin 2024 | 270/5906 | SMD | 4.04[3.21 to 11.28] | 13 | Fixed | 100;<0.001 | > 0.05 | > 0.05 | 8 | Class IV | | Very low |
| Vaccines | Conventional treatments | Malignant glioma | Skin reaction | 1 | Jajin 2024 | 199/3579 | RR | 3.654[1.711 to 7.801] | 7 | Random | 0;0.65 | > 0.05 | > 0.05 | 8 | Class IV | | Low |
| Vaccines | Conventional treatments | Malignant glioma | Flu-like syndrome | 1 | Jajin 2024 | 212/3667 | RR | 5.21[2.691 to 10.086] | 7 | Random | 0;0.83 | > 0.05 | > 0.05 | 8 | Class IV | | Moderate |
| Vaccines | Conventional treatments | Primary malignant glioma | OS duration | 1 | Jajin 2024 | 52/1069 | SMD | 8.18[0.33 to 10.02] | 6 | Random | NA(看不清 | > 0.05 | > 0.05 | 8 | Class IV | | Very low |
| Vaccines | Conventional treatments | High-grade gliomas | OS duration | 1 | Jajin 2024 | 66/1333 | SMD | 14.09[3.18 to 25.01] | 4 | Random | NA(看不清 | > 0.05 | > 0.05 | 8 | Class IV | | Very low |
| Cytoreductive resection | Biopsy | Supratentorial high-grade glioma | OS | 1 | Tsitlakidis 2010 | 338(total) | HR | 0.72[0.58 to 0.90] | 3 | Fixed | 31.2;0.23 | 0.49 | 0.49 | 8 | Class IV | | Very low |
| Cytoreductive resection | Biopsy | Grade IV/4 glioma | OS | 1 | Tsitlakidis 2010 | 1001(total) | HR | 0.61[0.45 to 0.82] | 3 | Random | 67.5;0.046 | 0.49 | 0.49 | 8 | Class IV | | Very low |
| Cytoreductive resection | Biopsy | Grade III or IV glioma | OS | 1 | Tsitlakidis 2010 | 110(total) | HR | 0.57[0.39 to 0.85] | 2 | Fixed | 6.9;0.3 | 0.49 | 0.49 | 8 | Class IV | | Very low |
| Cytoreductive resection | Biopsy | Elderly participants (≥ 65 years) with supratentorial high-grade glioma | OS | 1 | Tsitlakidis 2010 | 158(total) | HR | 0.50[0.35 to 0.71] | 2 | Fixed | 0;0.48 | 0.49 | 0.49 | 8 | Class IV | | Low |
| Cytoreductive resection | Biopsy | Supratentorial high-grade glioma with a wide age range | OS | 1 | Tsitlakidis 2010 | 953(total) | HR | 0.64[0.48 to 0.85] | 3 | Random | 62.6;0.07 | 0.49 | 0.49 | 8 | Class IV | | Very low |
| HFSRT + CT | Sole HFSRT management | Recurrent malignant glioma | OS | 1 | Hu 2019 | 122/84 | HR | 0.44[0.30 to 0.65] | 5 | Fixed | 14.8;0.32 | > 0.05 | > 0.05 | 7 | Class IV | | Low |
| HFSRT + CT | Sole HFSRT management | Recurrent malignant glioma | OS | 1 | Hu 2019 | 158/167 | MD | 0.80[0.41 to 1.18] | 6 | Random | 50.8;0.071 | > 0.05 | > 0.05 | 7 | Class IV | | Very low |
| Awake craniotomy | Asleep craniotomy | Eloquent glioma | Extent of resection | 3 | Sattari 2024 | 680/1123 | MD | 8.52[4.28 to 12.76] | 11 | Random | 94;<0.00001 | NA | No bias | 8 | Class IV | | Very low |
| Awake craniotomy | Asleep craniotomy | Eloquent glioblastoma multiforme | Extent of resection | 3 | Sattari 2024 | 299/656 | MD | 8.45[2.36 to 14.53] | 6 | Random | 90;<0.00001 | NA | No bias | 8 | Class IV | | Very low |
| Awake craniotomy | Asleep craniotomy | Eloquent glioma | OS | 3 | Sattari 2024 | 233/749 | MD | 2.86[1.35 to 4.37] | 4 | Random | 0;0.83 | NA | No bias | 8 | Class IV | | Low |
| Awake craniotomy | Asleep craniotomy | Eloquent glioma | PFS | 3 | Sattari 2024 | 229/511 | MD | 5.69[0.75 to 10.64] | 4 | Random | 86;<0.0001 | NA | No bias | 8 | Class IV | | Very low |
| Awake craniotomy | Asleep craniotomy | Eloquent glioma | 3-month postoperative neurological deficits | 3 | Sattari 2024 | 522/953 | OR | 0.47[0.28 to 0.78] | 10 | Random | 24;0.22 | NA | No bias | 8 | Class IV | | Low |
| Awake craniotomy | Asleep craniotomy | Eloquent glioma | 3-month postoperative Karnofsky performance score | 3 | Sattari 2024 | 125/291 | MD | 13.59[11.08 to 16.09] | 4 | Random | 18;0.30 | NA | No bias | 8 | Class IV | | Low |
| Awake craniotomy | Asleep craniotomy | Eloquent glioma | 3-month postoperative seizure freedom | 3 | Sattari 2024 | 72/61 | OR | 8.72[3.39 to 22.39] | 2 | Random | 0;0.96 | NA | No bias | 8 | Class IV | | Moderate |
| Awake craniotomy | Asleep craniotomy | Eloquent glioma | Length of hospital stay | 3 | Sattari 2024 | 108/101 | MD | -2.99[-5.09 to -0.88] | 3 | Random | 58;0.09 | NA | No bias | 8 | Class IV | | Very low |
| Adjuvant RT | Received either salvage RT or no RT at all | Oligodendroglioma | OS | 1 | Ng 2024 | 11812(total) | HR | 0.72[0.56 to 0.93] | 15 | Random | 86;<0.00001 | 0.1381 | 0.1381 | 9 | Class IV | | Very low |
| Adjuvant RT | Received either salvage RT or no RT at all | Oligodendroglioma | PFS | 1 | Ng 2024 | 1803(total) | HR | 0.52[0.40 to 0.66] | 12 | Random | 48;0.03 | 0.1381 | 0.1381 | 9 | Class IV | | Low |
| Adjuvant RT | Received either salvage RT or no RT at all | Oligodendroglioma defined by IDH mutation and 1p/19q co-deletion | OS | 1 | Ng 2024 | 1081/22 | HR | 0.72[0.56 to 0.92] | 6 | Random | 86;<0.00001 | 0.1381 | 0.1381 | 9 | Class IV | | Very low |
| Adjuvant RT | Received either salvage RT or no RT at all | Oligodendroglioma defined by IDH mutation and 1p/19q co-deletion | PFS | 1 | Ng 2024 | 1081/22 | HR | 0.49[0.34 to 0.72] | 7 | Random | 48;0.03 | 0.1381 | 0.1381 | 9 | Class IV | | Moderate |
| Adjuvant RT | Salvage RT | Oligodendroglioma | OS | 1 | Ng 2024 | 172/138 | HR | 0.60[0.41 to 0.89] | 10 | Random | 84;<0.00001 | 0.1381 | 0.1381 | 9 | Class IV | | Very low |
| Adjuvant CRT | RT alone | Oligodendroglioma | OS | 1 | Ng 2024 | 2340/4795 | HR | 0.66[0.57 to 0.77] | 5 | Random | 0;0.41 | 0.1381 | 0.1381 | 9 | Class IV | | Low |
| Adjuvant RT | Received either salvage RT or no RT at all | Grade 3 oligodendroglioma | OS | 1 | Ng 2024 | 157/344 | HR | 0.63[0.47 to 0.85] | 6 | Random | 55;0.05 | 0.1381 | 0.1381 | 9 | Class IV | | Very low |
| Adjuvant RT | Salvage RT | Oligodendroglioma | PFS | 1 | Ng 2024 | 7029(total) | HR | 0.42[0.28 to 0.61] | 8 | Random | 47;0.07 | 0.1381 | 0.1381 | 9 | Class IV | | Moderate |
| Adjuvant CRT | CT alone | Oligodendroglioma | PFS | 1 | Ng 2024 | 828(total) | HR | 0.47[0.36 to 0.60] | 4 | Fixed | 67;0.03 | 0.1381 | 0.1381 | 9 | Class IV | | Low |
| Adjuvant CRT | Adjuvant RT | Oligodendroglioma | PFS | 1 | Ng 2024 | 1055(total) | HR | 0.61[0.39 to 0.96] | 5 | Random | 78;0.001 | 0.1381 | 0.1381 | 9 | Class IV | | Very low |
| Adjuvant RT | Received either salvage RT or no RT at all | Grade 2 oligodendroglioma | PFS | 1 | Ng 2024 | 85/354 | HR | 0.46[0.33 to 0.66] | 4 | Random | 41;0.16 | 0.1381 | 0.1381 | 9 | Class IV | | Low |
| Adjuvant RT | Received either salvage RT or no RT at all | Grade 3 oligodendroglioma | PFS | 1 | Ng 2024 | 73/118 | HR | 0.51[0.36 to 0.73] | 6 | Random | 48;0.09 | 0.1381 | 0.1381 | 9 | Class IV | | Very low |
| Adjuvant RT | Adjuvant CT alone | Grade 3 to 5 oligodendroglioma | Adverse events | 1 | Ng 2024 | 240/247 | RR | 0.50[0.40 to 0.63] | 2 | Random | 0;0.53 | 0.1381 | 0.1381 | 9 | Class IV | | Low |
| Adjuvant CRT | Adjuvant RT | Grade 3 to 5 oligodendroglioma | Adverse events | 1 | Ng 2024 | 73/118 | RR | 3.24[1.19 to 8.86] | 2 | Random | 60;0.11 | 0.1381 | 0.1381 | 9 | Class IV | | Very low |
| CRT | RT | IDH-wild-type gliomas | PFS | 1 | Kinslow 2024 | 430(total) | HR | 0.77[0.62 to 0.97] | 5 | Random | 0;0.47 | NA | Bias | 6 | Class IV | | Moderate |
| CRT | RT | IDH-mutant gliomas | OS | 1 | Kinslow 2024 | 774(total) | HR | 0.52[0.42 to 0.64] | 5 | Random | 0;0.85 | NA | Bias | 6 | Class IV | | High |
| CRT | RT | IDH-mutant gliomas | PFS | 1 | Kinslow 2024 | 774(total) | HR | 0.47[0.39 to 0.57] | 6 | Random | 0;0.82 | NA | Bias | 6 | Class IV | | High |
| Alkylating CT | RT | IDH-mutant gliomas | PFS | 1 | Kinslow 2024 | 412(total) | HR | 1.57[1.14 to 2.16] | 4 | Random | 0;0.4 | NA | Bias | 6 | Class IV | | Moderate |
| CRT | RT | IDH-mutant and 1p19q-codeleted gliomas | OS | 1 | Kinslow 2024 | NA | HR | 0.59[0.43 to 0.81] | 4 | Random | 0;0.57 | NA | Bias | 6 | Class IV | | Moderate |
| CRT | RT | IDH-mutant and 1p19q-codeleted gliomas | PFS | 1 | Kinslow 2024 | NA | HR | 0.40[0.26 to 0.62] | 4 | Random | 29;0.24 | NA | Bias | 6 | Class IV | | High |
| CRT | RT | IDH-mutant and 1p19q-intact gliomas | OS | 1 | Kinslow 2024 | NA | HR | 0.51[0.40 to 0.65] | 4 | Random | 0;0.78 | NA | Bias | 6 | Class IV | | High |
| CRT | RT | IDH-mutant and 1p19q-intact gliomas | PFS | 1 | Kinslow 2024 | NA | HR | 0.49[0.39 to 0.61] | 4 | Random | 0;0.63 | NA | Bias | 6 | Class IV | | High |
| Anti-PD-1/PD-L1 treatment | RT and TMZ with or without placebo with/ BV | Glioma | OS | 1 | Zeng 2023 | 822/823 | HR | 1.15[1.03 to 1.29] | 3 | Fixed | 14;0.31 | 0.09 | 0.09 | 9 | Class IV | | Moderate |
| Anti-PD-1/PD-L1 treatment | Placebo with RT and TMZ | Glioma | PFS | 1 | Zeng 2023 | 822/823 | HR | 1.43[1.03 to 1.99] | 3 | Random | 87;<0.01 | 0.09 | 0.09 | 9 | Class IV | | Low |
| *Not Significant associations* | | | | | | | | | | | | | | | |  | |
| HFRT | CFRT | DIPG | OS | 1 | Park 2020 | 88/96 | HR | 1.07[0.77 to 1.47] | 4 | Fixed | 0;0.97 | >0.05 | >0.05 | 7 | NS | | Very low |
| HFRT | CFRT | DIPG | PFS | 1 | Park 2020 | 88/96 | HR | 1.04[0.75 to 1.45] | 4 | Fixed | 0;0.56 | >0.05 | >0.05 | 7 | NS | | Very low |
| BV plus RT/TMZ | RT/TMZ | High-grade gliomas | 6-month survival rate | 1 | Fu 2016 | 840/882 | OR | 0.65[0.37 to 1.13] | 3 | Random | 58;0.09 | NA | NA | 6 | NS | | Low |
| Viral therapy | Standard therapy | High-grade gliomas | PFS | 2 | Vatu 2019 | 203/279 | HR | 1.06[0.93 to 1.21] | 4 | Fixed | 88;<0.0001 | NA | Bias | 5 | NS | | Very low |
| DC vaccination | Control therapy | High-grade gliomas | 0.5-years OS | 3 | Li 2018 | 249/536 | RR | 1.06[0.93 to 1.20] | 11 | Fixed | 0;0.999 | >0.05 | >0.05 | 8 | NS | | Very low |
| DC vaccination | Control therapy | High-grade gliomas | 0.5-years PFS | 3 | Li 2018 | 61/160 | RR | 1.05[0.81 to 1.36] | 5 | Fixed | 0;0.975 | >0.05 | >0.05 | 8 | NS | | Very low |
| DC vaccination | Control therapy | High-grade gliomas | 1-years PFS | 3 | Li 2018 | 48/148 | RR | 1.55[0.75 to 3.23] | 4 | Random | 61.9;0.049 | >0.05 | >0.05 | 8 | NS | | Very low |
| CRT | RT alone | High-grade gliomas | Survival | 1 | Stewart 2002 | 1698/1306 | HR | 0.85[0.78 to 0.92] | 12 | Fixed | 0;0.28 | NA | NA | 7 | Class IV | | High |
| Gliadel | Placebo | High-grade gliomas | Survival | 1 | Hart 2008 | 272(total) | HR | 0.49 [ 0.19 to 1.23 ] | 2 | Random | 73;0.05 | NA | NA | 8 | NS | | Low |
| Gene therapy | Standard treatment | High-grade gliomas | Tumor progression | 1 | Zhao 2014 | 387/390 | OR | 1.31[0.96 to 1.79] | 4 | Fixed | 0;0.65 | NA | NA | 6 | NS | | Moderate |
| Gene therapy | Standard treatment | High-grade gliomas | OS | 1 | Zhao 2014 | 265/269 | HR | 0.91[0.74 to 1.13] | 3 | Fixed | 0;0.48 | NA | NA | 6 | NS | | Moderate |
| Gene therapy | Standard treatment | Glioblastoma | OS | 1 | Zhao 2014 | 134/134 | HR | 1.06[0.80 to 1.41] | 2 | Fixed | 0;0.71 | NA | NA | 6 | NS | | Moderate |
| Carmustine Wafers | Stupp Regimen | High-grade gliomas | PFS | 1 | Ricciardi 2022 | 506/695 | MD | 1.18[-2.69 to 5.04] | 4 | Random | 87;0.0004 | NA | NA | 6 | NS | | Very low |
| Combination of viral therapy and SOC | SOC alone | High-grade gliomas | OS | 2 | Guo 2023 | 913(total) | HR | 0.76[0.54 to 1.07] | 6 | Random | 70;<0.01 | NA | Bias | 9 | NS | | Very low |
| Combination of viral therapy and SOC | SOC alone | High-grade gliomas | PFS | 2 | Guo 2023 | 226(total) | HR | 0.52[0.22 to 1.22] | 2 | Random | 78;0.03 | NA | No bias | 9 | NS | | Very low |
| Combination of multiple courses of treatment/multi-point injection/ small injection volume viral therapy and SOC compared | SOC alone | High-grade gliomas | PFS | 2 | Guo 2023 | 226(total) | HR | 0.52[0.22 to 1.22] | 2 | Random | 78;0.03 | NA | No bias | 9 | NS | | Very low |
| Combination of DC therapy and SOC | SOC alone | High-grade gliomas | PFS | 2 | Guo 2023 | 90(total) | HR | 0.60[0.35 to 1.03] | 3 | Random | 0;0.39 | NA | Bias | 9 | Class IV | | Low |
| Combination of immunopotentiators and SOC | SOC alone | High-grade gliomas | OS | 2 | Guo 2023 | 79/87 | HR | 1.23[0.84 to 1.82] | 2 | Random | 0;0.77 | NA | Bias | 9 | NS | | Very low |
| TMZ | HFRT | Elderly patients (≥60 y) with high-grade gliomas | Survival | 1 | Hart 2013 | 119/123 | HR | 0.82[0.63 to 1.07] | 1 | Random | NA | NA | No bias | 9 | NS | | Low |
| TMZ | Standard RT | Elderly patients (≥60 y) with high-grade gliomas | Survival | 1 | Hart 2013 | 288/278 | HR | 0.88[0.57 to 1.63] | 2 | Random | 79.11;0.03 | NA | No bias | 9 | NS | | Low |
| TMZ | Standard RT | Elderly patients (≥60 y) with high-grade gliomas | PFS | 1 | Hart 2013 | 195/178 | HR | 1.15[0.92 to 1.44] | 1 | Random | NA | NA | No bias | 9 | NS | | Low |
| Dose‐dense TMZ | Metronomic TMZ high-grade gliomas | High-grade gliomas | Survival | 1 | Hart 2013 | 42/43 | HR | 0.84[0.51 to 1.40] | 1 | Random | NA | NA | No bias | 9 | Class IV | | Low |
| TMZ | Nitrosourea CT | Recurrent glioblastoma | Survival | 1 | Hart 2013 | 336/336 | OR | 0.9[0.76 to 1.06] | 2 | Random | 0;0.80 | NA | No bias | 9 | NS | | Moderate |
| TMZ | Nitrosourea CT | Recurrent glioblastoma | PFS | 1 | Hart 2013 | 336/336 | HR | 0.79[0.61 to 1.03] | 2 | Random | 56.47;0.13 | NA | No bias | 9 | NS | | Low |
| TMZ | Nitrosourea CT | Recurrent glioblastoma | Adverse events | 1 | Hart 2013 | 443/444 | OR | 1.19[0.81 to 1.75] | 3 | Fixed | 0;0.58 | NA | No bias | 9 | NS | | Moderate |
| Reirradiation | Systemic therapy | Recurrent high-grade glioma | OS | 1 | Marwah 2023 | 65/172 | HR | 0.94[0.67 to 1.31] | 3 | Random | 0;0.48 | NA | No bias | 10 | NS | | Very low |
| Reirradiation | Systemic therapy | Recurrent high-grade glioma | PFS | 1 | Marwah 2023 | 50/135 | HR | 0.87[0.61 to 1.22] | 2 | Random | 0;0.64 | NA | No bias | 10 | NS | | Very low |
| Combination therapy | Systemic therapy | Recurrent high-grade glioma | Toxicity-CTCAE Grade 3+ | 1 | Marwah 2023 | 208/190 | RR | 1.03[0.57 to 1.68] | 5 | Random | 21;0.28 | NA | No bias | 10 | NS | | Very low |
| Combination therapy | Systemic therapy | Recurrent high-grade glioma(RCT Only) | OS | 1 | Marwah 2023 | 104/101 | HR | 0.90[0.65 to 1.26] | 2 | Random | 7;0.30 | NA | No bias | 10 | NS | | Very low |
| Combination therapy | Systemic therapy | Recurrent high-grade glioma(RCT Only) | PFS | 1 | Marwah 2023 | 104/101 | HR | 0.51[0.22 to 1.19] | 2 | Random | 75;0.04 | NA | No bias | 10 | NS | | Very low |
| Combination therapy | Systemic therapy | Recurrent high-grade glioma(RCT Only) | Toxicity-CTCAE Grade 3+ | 1 | Marwah 2023 | 104/101 | HR | 1.13[0.71 to 1.82] | 2 | Random | 0;0.65 | NA | No bias | 10 | NS | | Very low |
| STR | Biopsy | Low-Grade Glioma | Mortality at 5 years | 3 | Brown 2019 | 433/356 | RR | 0.76[0.54 to 1.05] | 8 | Random | 76;0.0001 | NA | NA | 7 | NS | | Very low |
| STR | Biopsy | Low-Grade Glioma | Mortality at 10 years | 3 | Brown 2019 | 91/53 | RR | 0.95[0.73 to 1.23] | 3 | Random | 20;0.28 | NA | NA | 7 | NS | | Very low |
| Early Radiation | Late/No Radiation | Low-Grade Glioma | Mortality at 2 years | 3 | Brown 2019 | 1047/763 | RR | 0.92[0.53 to 1.58] | 9 | Random | 65;0.01 | NA | NA | 7 | NS | | Very low |
| Early Radiation | Late/No Radiation | Low-Grade Glioma | Mortality at 5 years | 3 | Brown 2019 | 1118/800 | RR | 0.93[0.60 to 1.43] | 10 | Random | 88;<0.00001 | NA | NA | 7 | NS | | Very low |
| Early Radiation | Late/No Radiation | Low-Grade Glioma | Mortality at 10 years | 3 | Brown 2019 | 856/484 | RR | 0.99[0.69 to 1.41] | 6 | Random | 94;<0.00001 | NA | NA | 7 | NS | | Very low |
| CT | RT | Low-Grade Glioma | Mortality at 2 years | 3 | Brown 2019 | 251/316 | RR | 1.34[0.85 to 2.12] | 5 | Random | 0;0.94 | NA | NA | 7 | NS | | Very low |
| CT | RT | Low-Grade Glioma | Mortality at 5 years | 3 | Brown 2019 | 251/316 | RR | 0.83[0.64 to 1.09] | 5 | Random | 0;0.62 | NA | NA | 7 | NS | | Very low |
| CT | RT | Low-Grade Glioma | Mortality at 10 years | 3 | Brown 2019 | 176/156 | RR | 0.77[0.58 to 1.03] | 3 | Random | 42;0.18 | NA | NA | 7 | NS | | Very low |
| CT | RT | Low-Grade Glioma | Progression at 2 years | 3 | Brown 2019 | 176/255 | RR | 0.92[0.64 to 1.33] | 3 | Random | 0;0.40 | NA | NA | 7 | NS | | Very low |
| STR | Biopsy | pHGGs | 1-Year Mortality | 1 | Hatoum 2022 | 423/262 | RR | 0.82[0.66 to 1.01] | 23 | Random | 18;0.22 | > 0.05 | > 0.05 | 8 | Class IV | | Very low |
| STR | Biopsy | pHGGs | 6-Month Progression | 1 | Hatoum 2022 | 224/169 | RR | 0.91[0.65 to 1.28] | 15 | Random | 31;0.12 | > 0.05 | > 0.05 | 8 | NS | | Very low |
| STR | Biopsy | pHGGs | 1-Year Progression | 1 | Hatoum 2022 | 221/169 | RR | 1.00[0.88 to 1.13] | 15 | Random | 3;0.42 | > 0.05 | > 0.05 | 8 | NS | | Very low |
| SMR | GTR | Glioblastoma, IDH wild-type | OS | 1 | Mier-García 2023 | 101/440 | HR | 0.74[0.46 to 1.19] | 4 | Random | 55;0.08 | NA | NA | 10 | Class IV | | Very low |
| GTR | STR | Glioma | 2-year PFS | 2 | Shi 2023 | 38/51 | RR | 1.82[0.98 to 3.37] | 2 | Random | 0;0.421 | > 0.05 | > 0.05 | 8 | NS | | Very low |
| GTR | STR | Glioma | 10-year PFS | 2 | Shi 2023 | 38/62 | RR | 1.33[0.89 to 1.99] | 5 | Random | 57.7;0.051 | > 0.05 | > 0.05 | 8 | NS | | Very low |
| GTR | STR | Glioma | PFS | 2 | Shi 2023 | 73/92 | WMD | 21.05[-14.05 to 56.16] | 2 | Random | 78.7;0.030 | > 0.05 | > 0.05 | 8 | NS | | Very low |
| GTR | STR | Glioma | Tumor progression | 2 | Shi 2023 | 54/64 | RR | 0.12[0.01 to 2.86] | 3 | Random | 79.8;0.007 | > 0.05 | > 0.05 | 8 | NS | | Very low |
| Conventional surgery | iMRI | Glioma | Mean extent of resection | 2 | Lo 2021 | 150/150 | MD | 1.17[-2.83 to 5.17] | 2 | Random | 85;0.01 | 0.92 | 0.92 | 8 | NS | | Low |
| Conventional surgery | iMRI | Glioma | PFS | 2 | Lo 2021 | 118/118 | HR | 0.78[0.58 to 1.06] | 2 | Random | 0;0.72 | NA | No bias | 8 | NS | | Moderate |
| Conventional surgery | iMRI | Glioma | OS | 2 | Lo 2021 | 200/166 | HR | 0.86[0.61 to 1.21] | 3 | Random | 33.7;0.22 | NA | No bias | 8 | NS | | Moderate |
| Conventional surgery | iMRI | Glioma | LOS | 2 | Lo 2021 | 200/166 | MD | 56.8[-4.7 to 118.4] | 4 | Random | 91;<0.00001 | NA | No bias | 8 | NS | | Low |
| Targeted combined CRT | CRT alone | Glioma | OS | 1 | Ma 2023 | 715/569 | RR | 0.92[0.79 to 1.08] | 12 | Random | 57.61;0.01 | 0.352 | 0.352 | 9 | NS | | Low |
| Targeted combined CRT | CRT alone | Glioma | PFS | 1 | Ma 2023 | 715/569 | HR | 0.90[0.63 to 1.27] | 11 | Random | 95.09;<0.0001 | 0.352 | 0.352 | 9 | NS | | Low |
| Targeted combined CRT | CRT alone | Glioma | Adverse events | 1 | Ma 2023 | 684/528 | OR | 0.50 [0.09 to 2.81] | 2 | Random | 69.14;NA | 0.352 | 0.352 | 9 | NS | | Low |
| Oral contraceptives | RT and TMZ | Glioma | Cumulative risk | 1 | Lan 2018 | 3436/6901 | OR | 0.99 [0.91 to 1.07] | 9 | Fixed | 37.6;0.13 | 0.779 | 0.779 | 9 | NS | | Very low |
| Hormone replacement therapy | RT and TMZ | Glioma | Cumulative risk | 1 | Lan 2018 | 2899131(total) | RR | 0.95[0.83 to 1.08] | 4 | Fixed | 0;0.961 | 0.891 | 0.891 | 9 | NS | | Very low |
| Immunotherapy | Standard care treatments(included surgical resection, RT, or CT) | Glioma | Mean survival time | 1 | Hanaei 2018 | 88/168 | MD | 1.51[-0.16 to 3.17] | 7 | Random | 63;0.01 | NA | No bias | 7 | NS | | Very low |
| Immunotherapy | Standard care treatments(included surgical resection, RT, or CT) | Glioma | 1-year PFS | 1 | Hanaei 2018 | 185/186 | HR | 0.94[0.74 to 1.18] | 3 | Random | 0;1.00 | NA | No bias | 7 | NS | | Very low |
| Awake craniotomy | General anesthetic resection | Glioma | Early language deficit | 1 | Bu 2021 | 368/222 | RR | 0.97[0.50 to 1.88] | 8 | Random | 60;0.02 | NA | No bias | 8 | NS | | Very low |
| Awake craniotomy | General anesthetic resection | Glioma | Late language deficit | 1 | Bu 2021 | 245/224 | RR | 0.54[0.26 to 1.13] | 7 | Random | 6;0.38 | NA | No bias | 8 | NS | | Very low |
| Awake craniotomy | General anesthetic resection | Glioma | Early motor deficit | 1 | Bu 2021 | 368/222 | RR | 0.65[0.34 to 1.26] | 8 | Random | 65;0.006 | NA | No bias | 8 | NS | | Very low |
| Awake craniotomy | General anesthetic resection | Glioma | Late motor deficit | 1 | Bu 2021 | 245/224 | RR | 0.66[0.34 to 1.25] | 7 | Random | 0;0.70 | NA | No bias | 8 | NS | | Very low |
| Awake craniotomy | General anesthetic resection | Glioma | Early neurological deficit | 1 | Bu 2021 | 388/242 | RR | 0.81[0.47 to 1.40] | 9 | Random | 84;<0.00001 | NA | No bias | 8 | NS | | Very low |
| Awake craniotomy | General anesthetic resection | Glioma | Extend of tumor resection | 1 | Bu 2021 | 499/334 | RR | 0.86[0.73 to 1.00] | 10 | Random | 42;0.09 | NA | No bias | 8 | NS | | Very low |
| Awake craniotomy | General anesthetic resection | Glioma | Mean operation time | 1 | Bu 2021 | 73/79 | MD | 51.32[-16.99 to 119.63] | 4 | Random | 91;<0.00001 | NA | No bias | 8 | NS | | Very low |
| Awake craniotomy | General anesthetic resection | Glioma | Mean hospital stay | 1 | Bu 2021 | 79/80 | MD | -1.25[-2.98 to 0.48] | 4 | Random | 60;0.06 | NA | No bias | 8 | NS | | Very low |
| Integrated Traditional Chinese and Western medicine | Western medical treatment | Glioma | One-year survival time | 1 | Yu 2021 | 100/90 | RR | 1.08[0.97 to 1.21] | 5 | Fixed | 42;0.18 | > 0.05 | > 0.05 | 8 | NS | | Moderate |
| Targeted therapy(including Regorafenib or Galunisertib or Depatux-M or Enzastaurin or Cediranib) | Lomustine | Glioblastoma | OS | 1 | Ippen 2024 | 450/343 | RR | 0.95[0.68 to 1.35] | 5 | Random | 75.85;0.7901 | 0.6301 | 0.6301 | 7 | NS | | Low |
| Targeted therapy(including Regorafenib or Galunisertib or Depatux-M or Enzastaurin or Cediranib) plus Lomustine | Lomustine | Glioblastoma | OS | 1 | Ippen 2024 | 645/402 | RR | 0.95[0.78 to 1.16] | 5 | Random | 35.59;0.6171 | 0.5839 | 0.5839 | 7 | NS | | Moderate |
| Targeted therapy(including Regorafenib or Galunisertib or Depatux-M or Enzastaurin or Cediranib) | BV | Glioblastoma | OS | 1 | Ippen 2024 | 657/611 | RR | 1.08[0.92 to 1.26] | 8 | Random | 31.77;0.3759 | 0.9607 | 0.9607 | 7 | NS | | Moderate |
| Targeted therapy(including Regorafenib or Galunisertib or Depatux-M or Enzastaurin or Cediranib) | Lomustine | Glioblastoma | PFS | 1 | Ippen 2024 | 411/303 | RR | 0.99[0.75 to 1.31] | 4 | Random | 62.26;0.9571 | 0.0297 | 0.0297 | 7 | NS | | Low |
| Targeted therapy(including Regorafenib or Galunisertib or Depatux-M or Enzastaurin or Cediranib) | BV | Glioblastoma | PFS | 1 | Ippen 2024 | 657/611 | RR | 1.04[0.80 to 1.35] | 9 | Random | 77.81;0.771 | 0.1025 | 0.1025 | 7 | NS | | Low |
| Targeted therapy | TMZ | Glioblastoma | OS | 1 | Scherm 2023 | 2024/1917 | RR | 0.98[0.86 to 1.11] | 11 | Random | 58.36;0.0093 | 0.8901 | 0.8901 | 7 | NS | | Low |
| Molecularly targeted drugs | TMZ plus RT | Glioblastoma | OS | 1 | Wang 2019 | 1604/1748 | OR | 0.96[0.89 to 1.04] | 15 | Fixed | 46;0.03 | > 0.05 | > 0.05 | 8 | NS | | Moderate |
| Antiangiogenic drugs | Cytotoxic drug alone | Glioblastoma | OS | 1 | Lombardi 2017 | 4330(total) | HR | 1.00[0.92 to 1.10] | 17 | Random | 21.2;0.2 | NA | No bias | 8 | NS | | Moderate |
| Higher dose of carmustine | Lower dose of carmustine | Glioblastoma | OS | 1 | Xiao 2020 | 27/138 | HR | 0.65[0.38 to 1.10] | 3 | Fixed | 0;0.11 | NA | No bias | 8 | NS | | Very low |
| Standard therapy + Anti-vascular endothelial growth factor | Standard therapy | Glioblastoma | OS | 1 | Xiao 2018 | 1078/946 | OR | 0.87[0.70 to 1.90] | 6 | Random | 74;0.002 | NA | NA | 7 | NS | | Low |
| Standard therapy + BV | Standard therapy | Glioblastoma | OS | 1 | Xiao 2018 | 859/848 | OR | 0.84[0.65 to 1.08] | 4 | Random | 77;0.005 | NA | NA | 7 | NS | | Low |
| Molecularly targeted drugs combined with TMZ plus RT | TMZ plus RT | Glioblastoma | OS | 1 | Su 2016 | 1301/1216 | HR | 0.936[0.852 to 1.028] | 5 | Fixed | 13.871;0.164 | NA | No bias | 8 | NS | | Moderate |
| Molecularly targeted drugs combined with TMZ plus RT | TMZ plus RT | MGMT-methylated patients | PFS | 1 | Su 2016 | 1050/1053 | HR | 0.888[0.573 to 1.379] | 3 | Random | 77.972;0.598 | NA | No bias | 8 | NS | | Low |
| Molecularly targeted drugs combined with TMZ plus RT | TMZ plus RT | MGMT-methylated patients | OS | 1 | Su 2016 | 805/664 | HR | 1.121[0.661 to 1.902] | 3 | Random | 43.205;0.671 | NA | No bias | 8 | NS | | Moderate |
| Molecularly targeted drugs combined with TMZ plus RT | TMZ plus RT | MGMT-nonmethylated patients | OS | 1 | Su 2016 | 483/480 | HR | 0.857[0.677 to 1.085] | 3 | Fixed | 0;0.2 | NA | No bias | 8 | NS | | Moderate |
| BV combined with TMZ plus RT | TMZ plus RT | Glioblastoma | PFS | 1 | Su 2016 | 838/840 | HR | 0.989[0.774 to 1.263] | 3 | Random | 47.891;0.929 | NA | No bias | 8 | NS | | Moderate |
| Cilengitide | TMZ plus RT | Glioblastoma | OS | 1 | Su 2016 | 251/163 | HR | 0.848[0.700 to 1.027] | 2 | Fixed | 0;0.092 | NA | No bias | 8 | NS | | Moderate |
| Levetiracetam plus SOC | SOC | Glioblastoma | OS | 1 | Chen 2022 | 1768/3747 | HR | 0.89[0.78 to 1.02] | 11 | Random | 75;<0.01 | NA | Bias | 8 | NS | | Very low |
| Levetiracetam plus SOC | Other AED treatment | Glioblastoma | Adverse events | 1 | Chen 2022 | 790/1362 | OR | 0.79[0.45 to 1.38] | 8 | Random | 55;0.05 | NA | Bias | 8 | NS | | Very low |
| BV plus other CRT | Other CRT | Newly diagnosed glioblastoma | All-Cause Discontinuation | 1 | Li 2015 | 643/810 | OR | 0.98[0.30 to 3.22] | 3 | Random | 91;<0.00001 | > 0.05 | > 0.05 | 8 | NS | | Low |
| BV plus other CRT | Other CRT | Newly diagnosed glioblastoma | Thrombocytopenia | 1 | Li 2015 | 515/519 | OR | 0.71[0.10 to 5.17] | 2 | Random | 83;0.01 | > 0.05 | > 0.05 | 8 | NS | | Low |
| BV plus other CRT | Other CRT | Newly diagnosed glioblastoma | Deep Vein Thrombosis | 1 | Li 2015 | 515/519 | OR | 2.16[0.19 to 25.16] | 2 | Random | 67;0.08 | > 0.05 | > 0.05 | 8 | NS | | Low |
| BV plus other CRT | Other CRT | Newly diagnosed glioblastoma | Pulmonary Embolism | 1 | Li 2015 | 515/519 | OR | 5.12[0.89 to 29.61] | 2 | Fixed | 0;0.35 | > 0.05 | > 0.05 | 8 | NS | | Moderate |
| BV in combination with CRT(including TMZ or Lomustine) | CRT alone(including TMZ or Lomustine) | Glioblastoma | OS | 1 | Lan 2022 | 2360/1261 | HR | 0.95[0.86 to 1.04] | 7 | Fixed | 16;0.30 | NA | NA | 7 | NS | | Moderate |
| Combination therapy of BV plus CT | BV or CT alone | Glioblastoma | OS | 1 | Yang 2017 | 607(total) | HR | 0.99[0.81 to 1.21] | 4 | Fixed | 36;0.20 | NA | NA | 8 | NS | | Low |
| High cumulative dose TMZ | Normal cumulative dose TMZ | Glioblastoma | OS | 1 | Sun 2015 | 1141(total) | HR | 1.07[0.94 to 1.22] | 3 | Fixed | 36;0.21 | > 0.05 | > 0.05 | 8 | NS | | Moderate |
| Higher peak concentration TMZ | lower peak TMZ | Glioblastoma | OS | 1 | Sun 2015 | 1141(total) | HR | 1.10[0.96 to 1.25] | 3 | Fixed | 13;0.32 | > 0.05 | > 0.05 | 8 | NS | | Moderate |
| High cumulative dose TMZ | Normal cumulative dose TMZ | Glioblastoma | PFS | 1 | Sun 2015 | 1056(total) | HR | 1.08[0.69 to 1.69] | 2 | Random | 88;0.005 | > 0.05 | > 0.05 | 8 | NS | | Low |
| HFRT | Conventional fraction RT | Glioblastoma | OS | 1 | Liao 2019 | 594(total) | HR | 0.94[0.72 to 1.22] | 4 | Random | 30;0.23 | NA | No bias | 8 | NS | | Moderate |
| HFRT | Conventional fraction RT | Glioblastoma | PFS | 1 | Liao 2019 | 157(total) | HR | 1.09[0.60 to 1.95] | 2 | Random | 65;0.09 | NA | No bias | 8 | NS | | Low |
| CRT | RT | Glioblastoma | PFS | 1 | Wang 2017 | 515/513 | HR | 0.75[0.56 to 1.01] | 4 | Random | 68;0.02 | NA | NA | 7 | NS | | Very low |
| RT plus TMZ | RT alone | Glioblastoma | Hematological complications | 4 | Zhao 2021 | 368/372 | RR | 0.76[0.40 to 1.45] | 3 | Random | 67;0.05 | NA | NA | 7 | NS | | Moderate |
| RT plus TMZ | RT alone | Glioblastoma | Serious adverse events | 4 | Zhao 2021 | 57/58 | OR | 2.20[0.55 to 8.70] | 2 | Random | 19;0.27 | NA | NA | 7 | NS | | Low |
| Anti-EGFR therapies | Placebo | Glioblastoma | OS | 1 | Lee 2020 | 1000(total) | HR | 0.89[0.76 to 1.04] | 3 | Random | 0;0.98 | NA | No bias | 9 | NS | | Moderate |
| Anti-EGFR therapies with CT | SOC with CT | Recurrent Glioblastoma | OS | 1 | Lee 2020 | 489(total) | HR | 0.79 [0.51 to 1.21] | 4 | Random | 77;0.005 | NA | No bias | 9 | NS | | Low |
| Anti-EGFR therapies | Placebo | Glioblastoma | PFS | 1 | Lee 2020 | 894(total) | HR | 0.94 [0.81 to 1.10] | 2 | Random | 0;0.95 | NA | No bias | 9 | NS | | Moderate |
| Anti-EGFR therapies | SOC | Glioblastoma | Lymphopenia | 1 | Lee 2020 | 597/549 | OR | 0.97[0.19 to 4.81] | 4 | Random | 89;<0.0001 | NA | No bias | 9 | NS | | Low |
| Anti-EGFR therapies | SOC | Glioblastoma | Neutropenia | 1 | Lee 2020 | 597/549 | OR | 1.29[0.82 to 2.03] | 4 | Random | 0;0.93 | NA | No bias | 9 | NS | | Moderate |
| Anti-EGFR therapies | SOC | Glioblastoma | Thrombocytopenia | 1 | Lee 2020 | 597/549 | OR | 3.69[0.51 to 26.51] | 4 | Random | 84;0.0002 | NA | No bias | 9 | NS | | Low |
| Anti-EGFR therapies | SOC | Glioblastoma | Rash | 1 | Lee 2020 | 597/549 | OR | 1.36[0.14 to 12.87] | 4 | Random | 52;0.15 | NA | No bias | 9 | NS | | Low |
| Anti-EGFR therapies | SOC | Glioblastoma | Diarrhoea | 1 | Lee 2020 | 632/586 | OR | 0.65[0.07 to 6.35] | 5 | Random | 0;0.56 | NA | No bias | 9 | NS | | Moderate |
| Anti-EGFR therapies | SOC | Glioblastoma | Fatigue | 1 | Lee 2020 | 632/586 | OR | 0.89[0.18 to 4.52] | 5 | Random | 64;0.04 | NA | No bias | 9 | NS | | Low |
| STR | Biopsy | Glioblastoma | 2-Year Mortality | 2 | Brown 2016 | 10251/3560 | RR | 0.99[0.97 to 1.00] | 16 | Random | 34;0.09 | NA | Bias | 8 | Class IV | | Very low |
| Resection | Biopsy | Glioblastoma | 2-Year Mortality | 2 | Brown 2016 | 15810/3585 | RR | 0.94[0.89 to 1.00] | 17 | Random | 96;<0.00001 | NA | Bias | 8 | Class IV | | Very low |
| GTR | STR | Glioblastoma | Progression at six months | 2 | Brown 2016 | 266/339 | RR | 0.72[0.48 to 1.09] | 6 | Random | 63;0.02 | NA | Bias | 8 | NS | | Very low |
| Resection | Biopsy | Glioblastoma | Progression at six months | 2 | Brown 2016 | 237/134 | RR | 0.72[0.51 to 1.00] | 4 | Random | 56;0.08 | NA | Bias | 8 | NS | | Very low |
| Lobectomy | GTR | Glioblastoma | KPS scores | 1 | Zheng 2023 | 66/81 | MD | 6.37[-13.80 to 26.54] | 3 | Random | 61;0.08 | NA | NA | 6 | NS | | Very low |
| Active immunotherapy | Standard therapy（combination of surgical resection, RT or CT） | Glioblastoma | OS | 1 | Wahyuhadi 2022 | 1202(total) | HR | 0.85[0.71 to 1.01] | 6 | Random | 54;0.06 | NA | NA | 8 | NS | | Very low |
| Active immunotherapy | Standard therapy（combination of surgical resection, RT or CT） | Glioblastoma | PFS | 1 | Wahyuhadi 2022 | 1202(total) | HR | 0.83[0.66 to 1.03] | 6 | Random | 44;0.11 | NA | NA | 8 | NS | | Low |
| BV plus other CRT | Other CRT | Recurrent glioblastoma | Median OS | 1 | LI 2016 | 1016/996 | HR | 1.01[0.83 to 1.23] | 5 | Random | 66;0.02 | > 0.05 |  | 10 | NS | | Moderate |
| BV plus some specific cytotoxic treatments | A certain cytotoxic treatment | Recurrent glioblastoma | Median OS | 1 | Zhang 2021 | 433/401 | MD | -0.19[-1.37 to 0.99] | 5 | Random | 0;0.52 | NA | NA | 7 | NS | | Moderate |
| BV plus some specific cytotoxic treatments | A certain cytotoxic treatment | Recurrent glioblastoma | 6-month PFS | 1 | Zhang 2021 | 187/155 | RR | 1.23[0.82 to 1.84] | 3 | Random | 17;0.30 | NA | NA | 7 | NS | | Moderate |
| BV plus some specific cytotoxic treatments | A certain cytotoxic treatment | Recurrent glioblastoma | 12-month OS | 1 | Zhang 2021 | 435/398 | RR | 0.93[0.79 to 1.09] | 5 | Random | 0;0.60 | NA | NA | 7 | NS | | Moderate |
| TMZ alone | RT alone | Elderly glioblastoma | OS | 1 | Yin 2014 | 401/555 | HR | 0.86[0.74 to 1.00] | 5 | Fixed | 12;0.34 | > 0.05 | > 0.05 | 8 | NS | | Very low |
| TMZ alone | RT alone | Elderly glioblastoma | PFS | 1 | Yin 2014 | 211/239 | HR | 0.98[0.64 to 1.51] | 2 | Random | 58;0.12 | > 0.05 | > 0.05 | 8 | NS | | Very low |
| GTR | Biopsy | Glioblastoma, IDH–wild-type | PFS | 1 | Jusue-Torres 2023 | 779(total) | MD | 3.51[-1.55 to 8.56] | 2 | Random | 91.2;<0.01 | NA | No bias | 9 | NS | | Very low |
| STR | Biopsy | Glioblastoma, IDH–wild-type | PFS | 1 | Jusue-Torres 2023 | 779(total) | MD | 0.03[-3.64 to 3.70] | 2 | Random | 92.8;<0.01 | NA | No bias | 9 | NS | | Very low |
| Intra-arterial CT | Intravenous CT | Malignant glioma | Disease control rate | 1 | Chen 2013 | 31/31 | RR | 1.11[0.86 to 1.44] | 2 | Fixed | 66;0.09 | NA | NA | 8 | NS | | Very low |
| Intra-arterial CT | Intravenous CT | Malignant glioma | Effective rate | 1 | Chen 2013 | 31/31 | RR | 1.22[0.43 to 3.43] | 2 | Fixed | 57;0.13 | NA | NA | 8 | NS | | Very low |
| Intra-arterial CT | Intravenous CT | Malignant glioma | Thrombocytopenia | 1 | Chen 2013 | 69/68 | RR | 0.34[0.04 to 3.14] | 3 | Fixed | 0;0.97 | NA | NA | 8 | NS | | Low |
| Intra-arterial CT | Intravenous CT | Malignant glioma | Anemia | 1 | Chen 2013 | 195/221 | RR | 0.73[0.46 to 1.17] | 4 | Fixed | 55;0.11 | NA | NA | 8 | NS | | Very low |
| Hyperfractionated RT | Conventional fractionation RT | Malignant glioma | 1-Year Mortality | 1 | Laperriere 2002 | 310/303 | RR | 0.89[.0.73 to 1.09] | 4 | Random | NA | NA | NA | 4 | NS | | Very low |
| Vaccines | Conventional treatments | Malignant glioma | Lymphopenia | 1 | Jajin 2024 | 199/3630 | RR | 21.96[0.322 to 1497.67] | 5 | Fixed | 91;<0.001 | > 0.05 | > 0.05 | 8 | NS | | Very low |
| Vaccines | Conventional treatments | Recurrent malignant glioma | OS duration | 1 | Jajin 2024 | 217/4070 | SMD | 2.41[-4.33 to 9.14] | 6 | Random | NA(看不清 | > 0.05 | > 0.05 | 8 | Class IV | | Very low |
| Vaccines | Conventional treatments | Low-Grade Glioma | OS duration | 1 | Jajin 2024 | 223/4596 | SMD | 1.45[-2.47 to 5.38] | 9 | Random | NA(看不清 | > 0.05 | > 0.05 | 8 | Class IV | | Very low |
| Awake craniotomy | Asleep craniotomy | Eloquent glioma | Duration of operation | 3 | Sattari 2024 | 108/101 | MD | 37.88[-34.09 to 109.86] | 3 | Random | 94;<0.00001 | NA | NA | 8 | NS | | Very low |
| Adjuvant RT | Adjuvant CT alone | Oligodendroglioma | OS | 1 | Ng 2024 | 221/193 | HR | 0.76[0.35 to 1.66] | 4 | Random | 89;<0.0001 | 0.1381 | 0.1381 | 9 | NS | | Very low |
| Adjuvant CRT | CT alone | Oligodendroglioma | OS | 1 | Ng 2024 | 849/1647 | HR | 0.45[0.20 to 1.01] | 4 | Random | 64;0.04 | 0.1381 | 0.1381 | 9 | NS | | Very low |
| Adjuvant RT | Received either salvage RT or no RT at all | Grade 2 oligodendroglioma | OS | 1 | Ng 2024 | 464/769 | HR | 0.67[0.43 to 1.05] | 6 | Random | 88;<0.00001 | 0.1381 | 0.1381 | 9 | NS | | Very low |
| Adjuvant RT | Adjuvant CT alone | Oligodendroglioma | PFS | 1 | Ng 2024 | 2730(total) | HR | 0.76[0.48 to 1.21] | 4 | Random | 64;0.04 | 0.1381 | 0.1381 | 9 | NS | | Very low |
| CRT | RT | IDH-wild-type gliomas | OS | 1 | Kinslow 2024 | 430(total) | HR | 0.87[0.64 to 1.18] | 5 | Random | 37;0.17 | NA | Bias | 6 | NS | | Low |
| Alkylating CT | RT | IDH-wild-type gliomas | PFS | 1 | Kinslow 2024 | 157(total) | HR | 1.41[0.32 to 6.13] | 2 | Random | 88;<0.1 | NA | Bias | 6 | NS | | Low |
| Neutron beam therapy | Photon therapy | High-grade gliomas | 12-month overall mortality | 1 | Maucort-Boulch 2010 | 159/157 | RR | 1.07[0.95 to 1.20] | 4 | Fixed | 0;0.66 | NA | No bias | 7 | NS | | Low |
| Neutron beam therapy | Photon therapy | High-grade gliomas | 24-month overall mortality | 1 | Maucort-Boulch 2010 | 159/157 | RR | 1.06[0.97 to 1.15] | 4 | Fixed | 49;0.12 | NA | No bias | 7 | NS | | Low |
| Reduced-dose BV | Standard -dose BV | Recurrent high-grade glioma or glioblastoma | OS | 1 | Chen 2020 | 552(total) | HR | 0.77[0.53 to 1.10] | 5 | Random | 76.9;0.002 | >0.05 | >0.05 | 8 | NS | | Very low |
| Reduced-dose BV | Standard -dose BV | Recurrent high-grade glioma or glioblastoma | PFS | 1 | Chen 2020 | 381(total) | HR | 0.66[0.37 to 1.20] | 4 | Random | 85.2;0.001 | >0.05 | >0.05 | 8 | NS | | Very low |
| Resection | Biopsy | Butterfly glioblastoma | Rates of postoperative defcit development | 3 | Soliman 2022 | 318/264 | OR | 1.86[0.87 to 4.00] | 13 | Random | 0;0.77 | NA | NA | 7 | Class IV | | Very low |
| Resection | Biopsy | Elderly patients (≥60 y) with high-grade gliomas | Morbidity | 1 | Almenawer 2015 | 427/433 | RR | 0.827[0.469 to 1.461] | 9 | Random | 44.3;0.514 | 0.05 | 0.05 | 8 | NS | | Very low |
| BV plus RT/TMZ | RT/TMZ | High-grade gliomas | OS | 1 | Fu 2016 | 848/890 | HR | 1.04[0.84 to 1.29] | 3 | Random | 67;0.05 | NA | NA | 6 | NS | | Low |
| CT | RT | IDH-mutant and 1p19q-codeleted gliomas | OS | 1 | Kinslow 2024 | NA | HR | 1.53[0.42 to 5.53] | 2 | Random | 27;0.24 | NA | Bias | 6 | NS | | Moderate |
| CT | RT | IDH-mutant and 1p19q-codeleted gliomas | PFS | 1 | Kinslow 2024 | NA | HR | 1.26[0.84 to 1.90] | 3 | Random | 0;0.46 | NA | Bias | 6 | NS | | Moderate |
| RT plus alkylating CT | RT | IDH-wild-type gliomas | OS | 1 | Kinslow 2024 | 393(total) | HR | 0.95[0.77 to 1.17] | 4 | Random | 0;0.72 | NA | Bias | 6 | NS | | Moderate |
| GTR, gross total resection; STR, subtotal resection; RT, radiotherapy; CT, chemotherapy; CRT, chemoradiotherapy; HFSRT, hypofractionated stereotactic radiotherapy; SOC, standard of care; BV, bevacizumab; TMZ, temozolomide; AED, anti-epileptic drug; EGFR, epidermal growth factor receptor; HFRT, hypofractionated radiotherapy; CFRT, conventional fractionated radiotherapy; DIPG, diffuse intrinsic pontine gliomas; iMRI, intraoperative magnetic resonance imaging. | | | | | | | | | | | | | | | |  | |
